# CH_2_ Linkage Effects on the Reactivity of Bis(aminophosphine)–Ruthenium Complexes for Selective Hydrogenation of Esters into Alcohols

**DOI:** 10.1038/s41598-017-04362-9

**Published:** 2017-06-21

**Authors:** Xiaolong Fang, Mingjun Sun, Jianwei Zheng, Bin Li, Linmin Ye, Xiaoping Wang, Zexing Cao, Hongping Zhu, Youzhu Yuan

**Affiliations:** 1 State Key Laboratory of Physical Chemistry of Solid Surfaces, National Engineering Laboratory for Green Chemical Productions of Alcohols–Ethers–Esters, iChEM, College of Chemistry and Chemical Engineering, Xiamen University, Xiamen, 361005 China; 2 State Key Laboratory of Physical Chemistry of Solid Surfaces, Fujian Provincial Key Laboratory of Theoretical and Computational Chemistry, College of Chemistry and Chemical Engineering, Xiamen University, Xiamen, 361005 China

## Abstract

A novel ruthenium complex binding to two subtly different aminophosphine ligands, (*o*-PPh_2_C_6_H_4_CH_2_NH_2_)(*o*-PPh_2_C_6_H_4_NH_2_)RuCl_2_, was successfully isolated. This bis(aminophosphine)–ruthenium complex shows efficient activity in both dimethyl oxalate (DMO) and methyl benzoate (MB) hydrogenation. On the contrast, similar complexes (*o*-PPh_2_C_6_H_4_NH_2_)_2_RuCl_2_ and (*o*-PPh_2_C_6_H_4_CH_2_NH_2_)_2_RuCl_2_, can only effectively catalyze the hydrogenation of DMO and MB, respectively. Our experimental studies in combination of theoretical calculations reveal that the remarkable substrate selectivity in the hydrogenation of esters arises from the nonbonding interactions operated by the CH_2_ linkage of the ligand.

## Introduction

The function and importance of nonbonding interactions have been recognized in enzymatic catalysis^1, 2^, chiral organometallic catalysis^3–5^, chiral organocatalysis^6–9^, and small molecular conversions^10, 11^, and they can influence the reaction efficiency and stereoselectivity^12–15^. These weak interactions are becoming an essential component that can even govern the reaction mechanism and may facilitate the design of new catalysts^16–18^. Normally, these weak interactions keep step with the change of the steric and electronic environments. However, efficient modulation of nonbonding interactions during catalysis is still of great challenge for chemists^15^.

**Figure 1:**
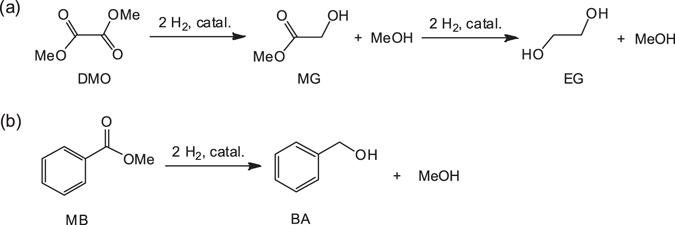
(**a**) Catalytic hydrogenation of DMO into MG (or EG). (**b**) Catalytic hydrogenation of MB into BA.

Metal–NH catalysis, a representative system in organometallic catalysis of small molecules^19–23^, has been increasingly used to improve the hydrogenation of esters into alcohols in the last decade^24–35^. Two main mechanisms, namely, Noyori-Ikariya-type out-sphere pathway^32–34^ and classical inner-sphere pathway^28, 29^, have been proposed. However, the existing mechanism can not well explain some cases of hydrogenation^28, 36, 37^. For instance, the ruthenium complexes [(PPh_3_)(*o*-PPh_2_C_6_H_4_NH_2_)RuCl_2_]_2_ (**1**) and (*o*-PPh_2_C_6_H_4_NH_2_)_2_RuCl_2_ (**2**) containing the rigid ligand *o*-PPh_2_C_6_H_4_NH_2_ can efficiently catalyze the hydrogenation of several aliphatic and cyclic esters to corresponding alcoholic compounds, but they show poor activities in the conversion of aromatic esters^38^. For example, they can be applied in DMO hydrogenation into methyl glycolate (MG, Fig. 1a), but are not suitable for MB hydrogenation into benzyl alcohol (BA, Fig. 1b). Unpredictably, the complex (*o*-PPh_2_C_6_H_4_CH_2_NH_2_)_2_RuCl_2_ (**3**), which exhibits a structure similar to that of **2**, effectively converts aromatic esters but show only moderate activities in the hydrogenation of oxalate esters. The distinct difference between the activities of complexes **3** and **2** containing ligands with and without CH_2_ linkage is interesting.

Herein, the new ruthenium complex (*o*-PPh_2_C_6_H_4_CH_2_NH_2_)(*o*-PPh_2_C_6_H_4_NH_2_)RuCl_2_ (**4**) contains two different aminophosphine ligands (N,P-ligands) similar to those in **2** and **3**; the complex exhibits satisfactory performance both in the hydrogenation of oxalate and aromatic esters. The significant effect of the CH_2_ linkage of the *o*-PPh_2_C_6_H_4_CH_2_NH_2_ ligand (also named as soft ligand), whcih is demonstrated ascribable to the nonbonding interactions between the ruthenium catalyst and substrates, was confirmed by comprehensive experimental and theoretical methods.

## Results and Discussion

**Figure 2:**
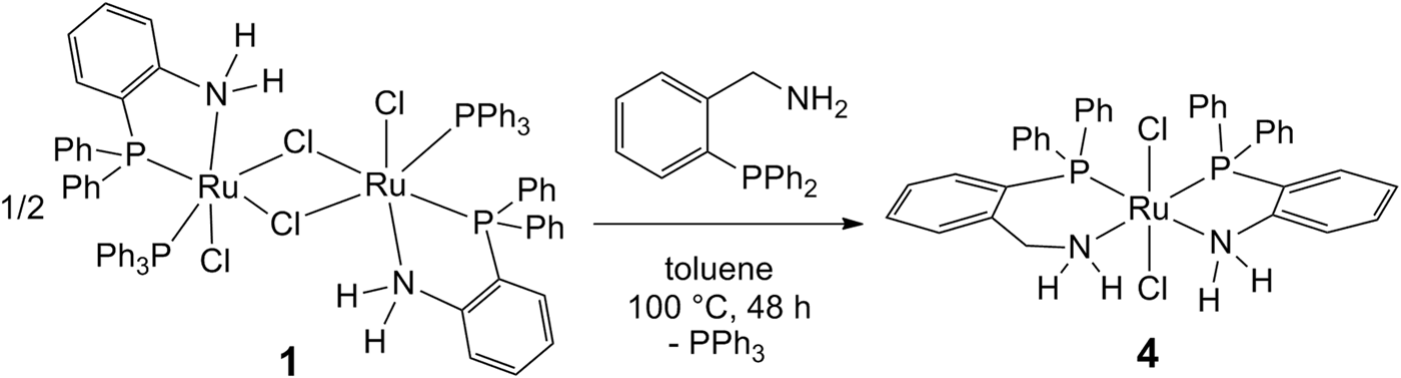
Synthesis of ruthenium complex **4**.

**Figure 3:**
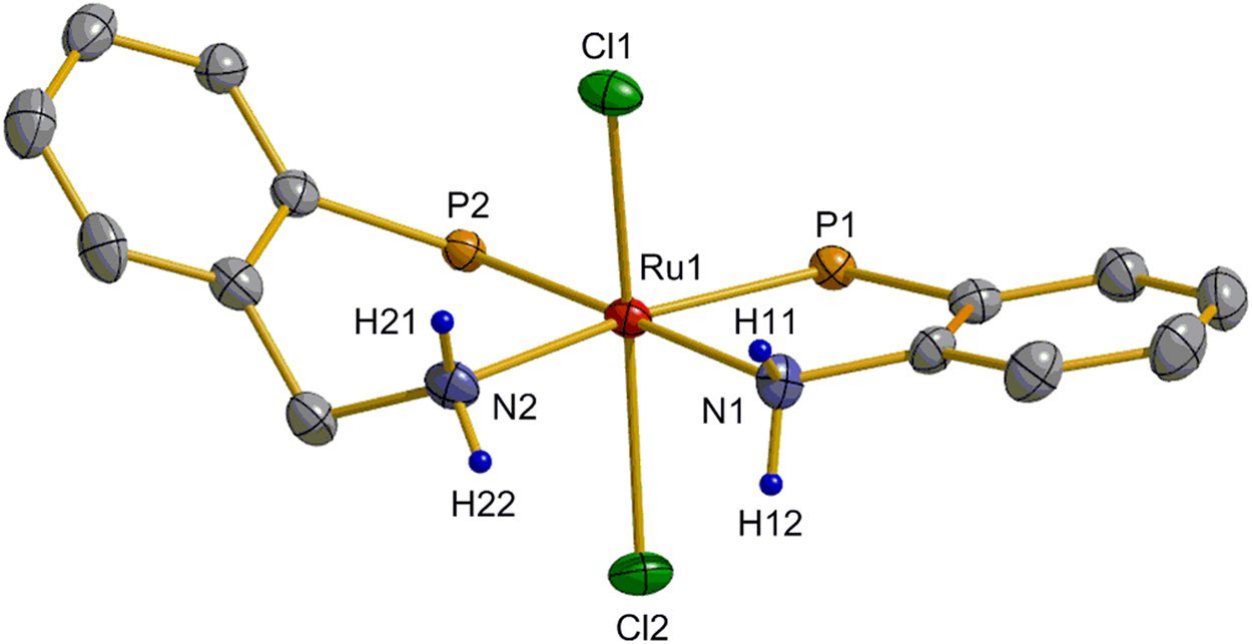
X-ray molecular structure of 4 with thermal ellipsoids at 50% probability level. The C_6_H_5_ groups at the P atom and the C_6_H_4_ and CH_2_ H atoms are omitted for clarity.

The reaction of **1** with two equivalents of *o*-PPh_2_C_6_H_4_CH_2_NH_2_ successfully produced **4** with 86% yield (Fig. 2). Complex **4** was characterized by spectroscopic methods (^1^H, ^13^C{^1^H}, and ^31^P{^1^H} NMR and IR) and X-ray single crystal diffraction. The ^31^P{^1^H} NMR spectrum indicates the incorporation of the two inequivalent N,P-ligands at the ruthenium center of complex **4**, which exhibits two doublets with significant P-P coupling [^2^ *J*(P,P) = 30.3 Hz; Supplementary Information (SI), section I]. The X-ray diffraction pattern of complex **4** reveals that the ruthenium atom adopts a distorted octahedral geometry (Fig. 3), where the two N,P-ligands bind to the ruthenium center in *cis*-arrangement and form an equatorial RuN_2_P_2_ coordination plane. The two Cl atoms are located *trans* to each other at the axial position. Apparently, the introduction of the CH_2_ linkage reduces the steric hindrance but increases the electronic density around the NH_2_ group.

**Figure 4:**
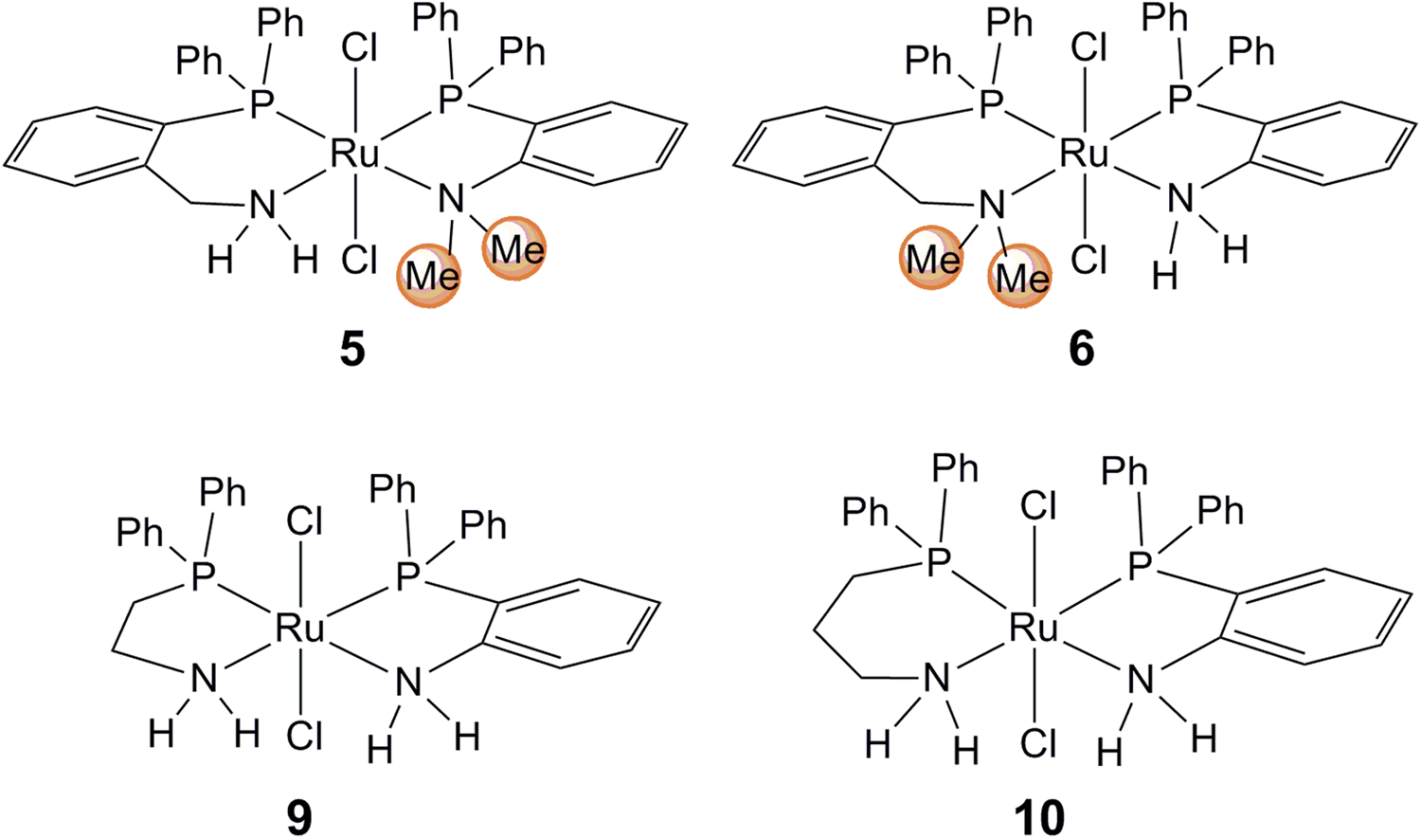
Structures of ruthenium complexes **5**, **6**, **9**, and **10**.

As shown in Fig. 4, the four complexes that contain two inequivalent N,P-ligands at the ruthenium center, (*o*-PPh_2_C_6_H_4_CH_2_NH_2_)(*o*-PPh_2_C_6_H_4_NMe_2_)RuCl_2_ (**5**), (*o*-PPh_2_C_6_H_4_CH_2_NMe_2_)(*o*-PPh_2_C_6_H_4_NH_2_)RuCl_2_ (**6**), (PPh_2_CH_2_CH_2_NH_2_)(*o*-PPh_2_C_6_H_4_NH_2_)RuCl_2_ (**9**), and [PPh_2_(CH_2_)_3_NH_2_](*o*-PPh_2_C_6_H_4_NH_2_)RuCl_2_ (**10**) were also successfully synthesized and spectroscopically characterized. The X-ray single crystal structures of complexes **5** and **6** are shown in Fig. S2 and S3, respectively, which feature similar skeletal structures with that of complex **4**, but one of the NH_2_ groups in *o*-PPh_2_C_6_H_4_NH_2_ and *o*-PPh_2_C_6_H_4_CH_2_NH_2_ is replaced with the NMe_2_ group. Complexes **9** and **10** also contain rigid ligand *o*-PPh_2_C_6_H_4_NH_2_ like **4**, while the soft ligand *o*-PPh_2_C_6_H_4_CH_2_NH_2_ is changed to PPh_2_CH_2_CH_2_NH_2_ and PPh_2_(CH_2_)_3_NH_2_, respectively.

**Figure 5:**
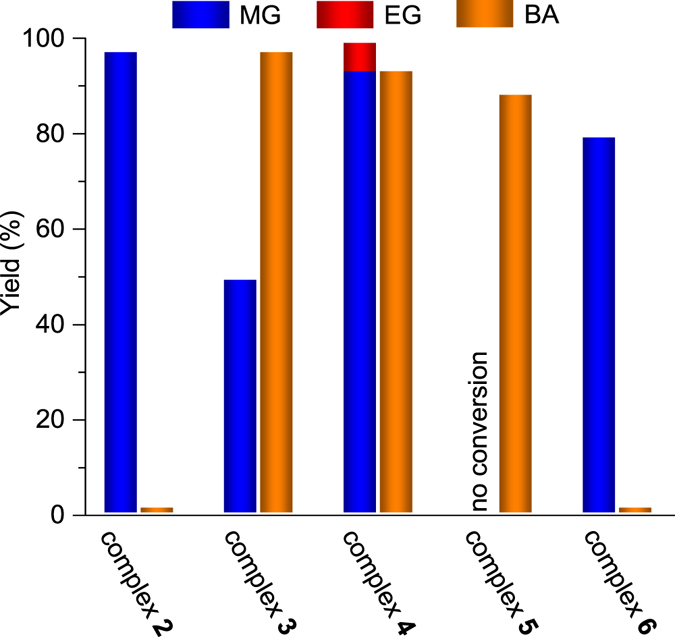
Catalytic performance of complexes **2–6** for hydrogenation of DMO into MG (and/or EG) and that of MB into BA. Reaction conditions: 7.57 mmol ester, 0.5 mol% ruthenium, 5 mol% (for DMO) or 10 mol% (for MB) NaOMe, 10 mL THF, 50 bar H_2_, 100 °C, 4 h. Decarbonylation occurred in DMO hydrogenation when using 0.5 mol% **3** and 10 mol% NaOMe.

Complex **4** was used to catalyze the hydrogenation of DMO and MB at 100 °C and 50 bar H_2_ for 4 h. As shown in Fig. 5, complex **4** exhibited satisfactory activities in the conversions, and the corresponding alcohols were obtained in excellent yields [93% MG and 6% ethylene glycol (EG) for DMO, 93% BA for MB]. In the absence of complex **4** or NaOMe, no hydrogenation product was obtained (Table S1). Similar to our previous report^38^, complex **2** promoted DMO hydrogenation into MG but was inactive in MB transformation into BA; the yields of MG and BA were 97% and 1%, respectively. The hydrogenation rate of MB increased as a result of introducing the CH_2_ linkage into one of the *o*-PPh_2_C_6_H_4_NH_2_ ligands. Under the same conditions, the corresponding yields of MG and BA for complex **3** were 49% and 93%, respectively.

**Figure 6:**
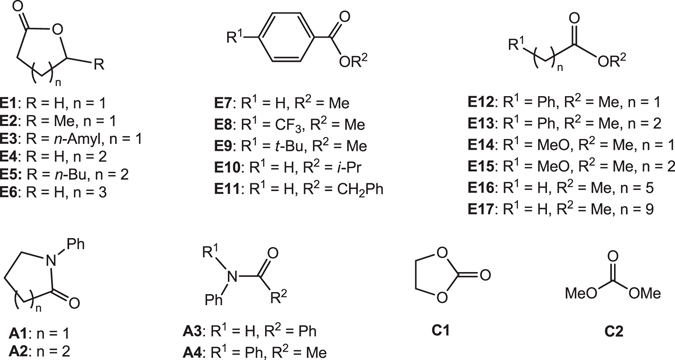
Substrates tested in this work.

Encouraged by the success of complex **4** in DMO and MB hydrogenation, we further investigated hydrogenation of various substrates shown in Fig. 6, under different reaction conditions (Table 1). As listed in entries 1-6, 7–11 and 12–17, respectively, lactones (**E1**–**E6**), aromatic esters (**E7**–**E11**), and aliphatic esters (**E12**–**E17**) all were converted smoothly and the corresponding alcohols were obtained in moderate to good yields (49–100%). Actually, complex **4** also exhibited good activity in ester hydrogenation under lower H_2_ pressure (Table S2). Under the conditions at 100 °C and 50 bar H_2_ for 10 h, complex **4** showed good activity in the hydrogenation of carboxamides **A1**–**A4**, and desired alcohols were obtained in yields between 64–100% (entries 18–21). Surprisingly, after increasing the reaction temperature from 100 °C to 120 °C, complex **4** could also catalyze the hydrogenation of ethylene carbonate (**C1**) and dimethyl carbonate (**C2**), and the methanol yield attained 91% and 44% (entries 22 and 23), respectively. Thus, the combination of the rigid *o*-PPh_2_C_6_H_4_NH_2_ and soft *o*-PPh_2_C_6_H_4_CH_2_NH_2_ ligands in complex **4** significantly promotes the reaction efficiency.

**Table 1:** Hydrogenation of substrates shown in Fig. 6 with **4**.

Entry	Substrate	Ru/mol%	Time/h	Conv./%^*a*^	Yield/%^*a*^
1	**E1**	0.1	6	97	97
2	**E2**	0.1	6	85	85
3	**E3**	0.1	6	69	69
4	**E4**	0.1	6	100	99
5	**E5**	0.1	6	100	99
6	**E6**	0.1	6	93	91
7	**E7**	0.2	6	68	64
8	**E8**	0.2	6	92	92
9	**E9**	0.2	6	75	75
10	**E10**	0.2	6	74	73
11	**E11**	0.2	6	100	98
12	**E12**	0.2	6	93	92
13	**E13**	0.2	6	76	73
14	**E14**	0.2	6	100	99
15	**E15**	0.2	6	99	98
16	**E16**	0.2	6	73	49^*b*^
17	**E17**	0.2	12	75	60^*c*^
18^*d*^	**A1**	1	10	87	86
19^*d*^	**A2**	1	10	100	99
20^*d*^	**A3**	1	10	70	64
21^*d*^	**A4**	1	10	100	99
22^*e*^	**C1**	1	6	93	91
23^*e*^	**C2**	1	6	54	44

Reaction conditions: 7.57 mmol substrate, the molar ratio of NaOMe to ruthenium was 20, 10 mL THF, 50 bar H_2_, 100 °C. ^*a*^ Unless otherwise noted, conversion of substrate and yield of alcohol were analyzed by gas chromatograph (GC). ^*b*^ 24% fatty-fatty esters present. ^*c*^ 14% fatty-fatty esters present. ^*d*^ Carboxamide conversion and alcohol yield were analyzed by ^1^H NMR spectroscopy. ^*e*^ 120 °C, NaOEt was used.

**Figure 7:**
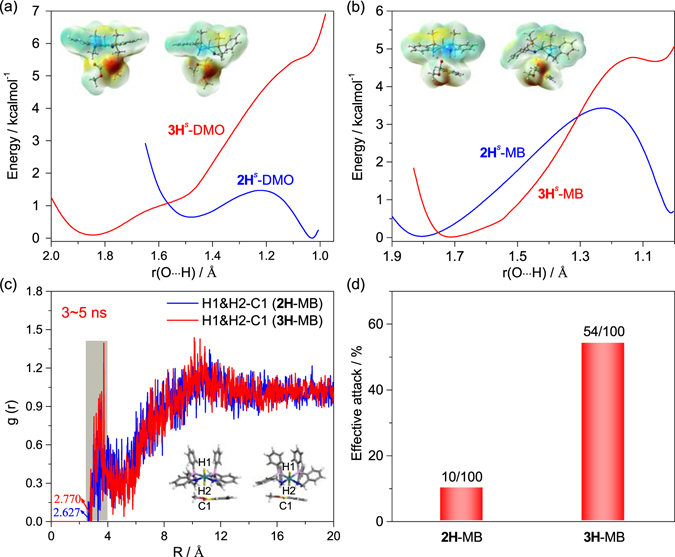
(**a**) The flexible scanning for the NH proton transfer (N–H → O^−^–CH) in DMO hydrogenation. (**b**) The flexible scanning for the NH proton transfer (N–H → O^−^–CH) in MB hydrogenation. (**c**) Calculated partial pair correlation function g(r) for the distance between the ruthenium hydride and carbonyl carbon of MB in 3–5 ns. (**d**) Percentage of effective attack to MB in **2H** and **3H**.

**Figure 8:**
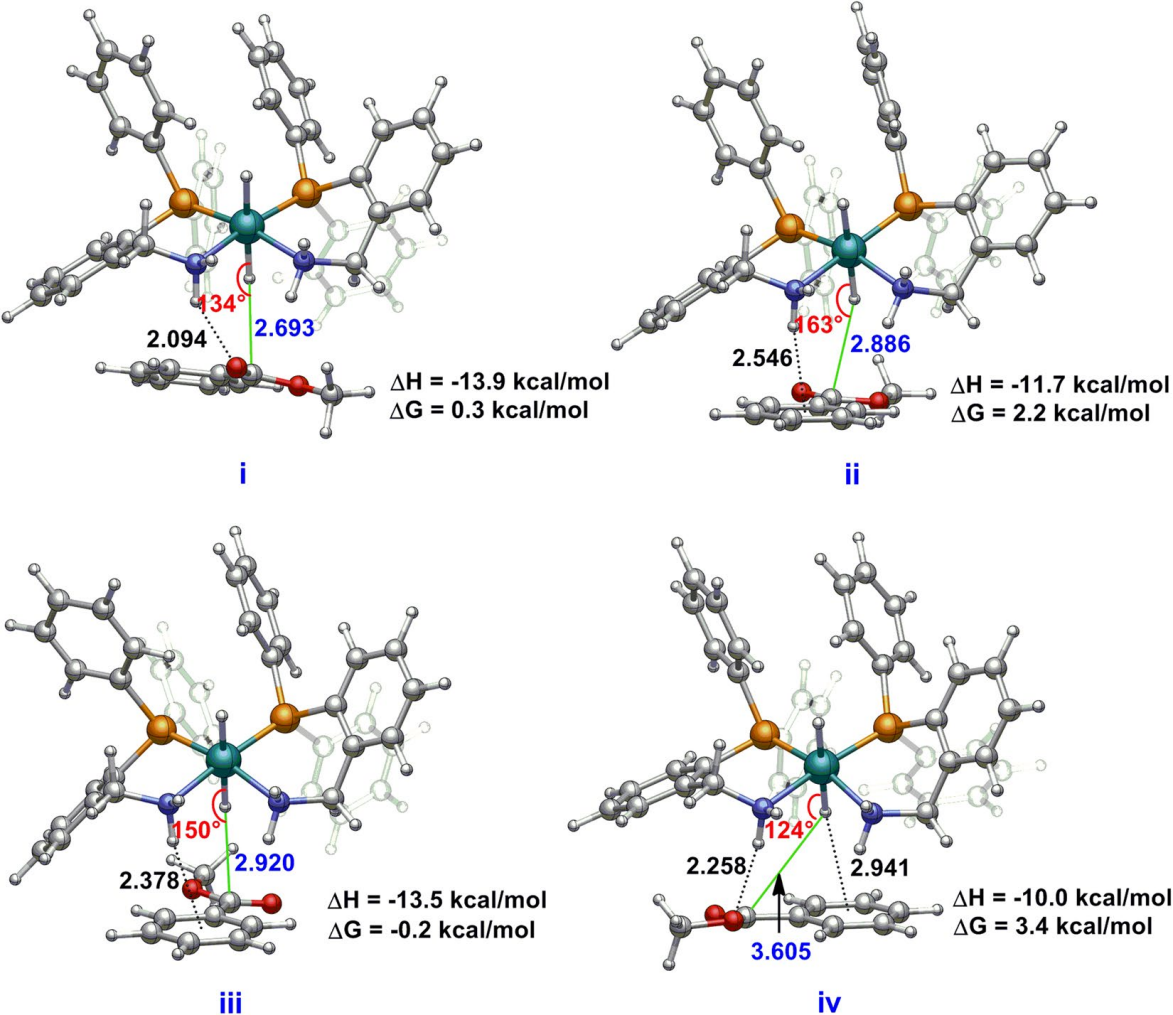
Optimized structures of the reactive conformers for the reaction system 3H-MB by DFT + D calculations.

According to the slight difference of these two ligands, we also determined the manipulation of the CH_2_ linkage in the catalytic performance, where we performed extensive density functional theory (DFT) calculations and molecular dynamics (MD) simulations on the proposed catalysts **2H** and **3H**, and their corresponding **2H** ^*s*^ and **3H** ^*s*^ models (Fig. S6). The results are displayed in Table 2, Figs 7 and 8. Details are provided in Fig. S6–20 (SI, section IV).

**Table 2:** Predicted relative Gibbs free energies for hydrogen transfers involved in the hydrogenation of esters.

Entry	Reaction system	Gas Phase					Solution	
		TS1	TS1^*s*^	IN2^*s*^	TS2^*s*^	IN3^*s*^	TS1	TS1^*s*^
1	**2H**-DMO	10.7	8.2	6.7	8.2	8.9	6.4	7.6
2	**3H**-DMO	12.4	10.7	2.9	—	—	8.2	9.4
3	**2H**-MB	19.3	15.9	2.3	2.8	0.2	13.7	14.7
4	**3H**-MB	21.6	17.3	6.0	9.1	7.9	16.8	15.0

Unit in kcal/mol. “^*s*^”refers to **2H** ^***s***^ and **3H** ^***s***^, which is the simplified model of **2H** and **3H**.

The entire catalytic hydrogenation of esters into alcohols can be assumed to proceed through two stages: hydrogenation of esters into aldehydes (stage I) and subsequent hydrogenation into alcohols (stage II)^31, 39–41^. Our calculations (Figs S11 and S12) and previous studies^36, 42, 43^ propose that stage II is more favorable than stage I both kinetically and thermodynamically. Accordingly, stage II might be insignificant for evaluation of ester hydrogenation here, and this is also supported by the experimental fact that complexes **2** and **3** exhibit identical performances in benzaldehyde hydrogenation (Table S3).

The optimized structures of transition states and intermediates for **2H** ^*s*^ catalyzing hydrogenation of MB into benzaldehyde and methanol are collected in Fig. S8, and the corresponding relative free-energy profiles are shown in Fig. S9. Here, two possible reactive configurations with the outward or inward carbonyl (refer to **2H** ^*s*^-TS1_MB_ and **2H** ^*s*^-TS1_MB_′ in Fig. S8) for MB have been considered, and the results show that the initial hydride transfer to the outward carbonyl is more favorable, which experiences a relatively low free energy barrier. The low-energy reaction channel follows a multi-step mechanism (Fig. S7)^31, 40^, where the initial hydride transfer from ruthenium to the carbonyl carbon of the ester experiences a relatively high energy barrier and it might be the rate-determining step. Further calculations reveal that the hydrogenation of DMO by the ruthenium catalysts follows a similar mechanism of the hydrogenation of MB. The optimized structures of the transition states (TS1) are depicted in Fig. S10.

Table 2 lists the predicted thermodynamic quantities of the hydrogen transfer in DMO and MB hydrogenation. The relatively low free energy barriers less than 17 kcal/mol in solution suggest that the initial hydride transfer in DMO and MB hydrogenation can occur at room temperature. This is consistent with the experimental fact that **2** and **3** can catalyze the hydrogenation of DMO and MB at room temperature, respectively. As is shown in Table 2 (entries 1 and 2) and Fig. 7a, the DFT-predicted free energy barriers indicate that complex **2** is more active toward DMO hydrogenation than complex **3** as observed experimentally (refer to Fig. 5). On the contrast, experimentally, complex **3** is much more active than complex **2** for MB hydrogenation (Fig. 5), while DFT calculations suggest that both catalysts exhibit comparable activities (Table 2 and Fig. 7b).

As mentioned above, the hydrogenation reaction is initiated by the hydride transfer from ruthenium to the carbonyl carbon of ester and this step is influenced by the carbonyl position (Fig. S7–9). Considering the inconsistency of the experimental observations and DFT calculations, we performed extensive MD simulations to investigate the steric and attractive nonbonding interaction effect on these bimolecular reactions. As shown in Fig. 7c and d, the simulations reveal that the reaction system of **3H**-MB possesses more effective reaction conformers than **2H**-MB; the former has remarkably higher conformer contributions in the reactive region of 2.5–3.5 Å between the ruthenium hydride and the carbonyl carbon of MB from MD simulations in 3–5 ns. In addition, the snapshots of the reactive systems reveal the optimal attack configuration for the initial hydride transfer, where the Ru−H group is approximately perpendicular to the carbonyl group of MB (Fig. S17). These configurations can survive in a larger reactive region for the reaction system of **3H**-MB compared with that of **2H**-MB. Further geometry optimizations of the near attack reactive configurations by DFT + D show that the Ru–H···C angles of conformers **ii** and **iii** in the reaction system of **3H**-MB (Fig. 8) is larger than the sole conformer **i** in the **2H**-MB reaction system (Fig. S18). This finding indicates that the attractive nonbonding interactions (N–H···O and N–H···π, Fig. 8) and the less steric hindrance operated by the CH_2_ linkage dominate the accessibility of the reactive conformer. Therefore, the activity difference between complexes **2** and **3** toward MB hydrogenation exclusively originates from the accessibility of the near attack reactive conformer and their relative stabilities.

Interestingly, complex **4**, bearing two kinds of N,P-ligands with or without the CH_2_ linkage appearing in complexes **3** and **2**, generally exhibits superior performance in the hydrogenation of DMO and MB. Previous studies reported that the NH proton for the metal-NH catalysis is crucial and indispensible in hydrogenation^24, 44, 45^. Accordingly, we synthesized two complexes, namely, (*o*-PPh_2_C_6_H_4_CH_2_NH_2_)(*o*-PPh_2_C_6_H_4_NMe_2_)RuCl_2_ (**5**) and (*o*-PPh_2_C_6_H_4_CH_2_NMe_2_)(*o*-PPh_2_C_6_H_4_NH_2_)RuCl_2_ (**6**), to elucidate the critical roles of the two inequivalent NH_2_ groups in hydrogenation (Fig. 4).

Activity tests show that no conversion was observed using complex **5** in DMO hydrogenation; however, the activity of complex **5** in MB hydrogenation had a BA yield of 88%, which is comparable with that of complex **4** (Fig. 5). Interestingly, complex **6** exhibited opposite performance in DMO and MB hydrogenation to that of complex **5**, and the corresponding yields were 79% and 1%, respectively. The shutdown of the activity in DMO or MB hydrogenation after NMe_2_ group substitution shows that the NH_2_ groups of *o*-PPh_2_C_6_H_4_NH_2_ and *o*-PPh_2_C_6_H_4_CH_2_NH_2_ in complex **4** are indispensable in hydrogenation; nevertheless, these groups are inclined to participate in the hydrogenation of DMO and MB, respectively, similar to those in complex **2** or **3**. These results are consistent with the computational results, indicating that the function of the CH_2_ linkage in complex **4** is similar to that in complex **3**. The CH_2_ linkage facilitates the modulation of attractive nonbonding and steric interactions between the catalyst and the ester MB. Hence, the co-coordination of the rigid *o*-PPh_2_C_6_H_4_NH_2_ and soft *o*-PPh_2_C_6_H_4_CH_2_NH_2_ ligands in complex **4** positively influences DMO and MB hydrogenation.

Furthermore, the catalytic performances of complexes **9** and **10** in ester hydrogenation were examined under the same conditions as in Fig. 5. The results in Fig. S5 indicated that both **9** and **10** could exhibit exceptional performances in DMO hydrogenation (86% yield of MG and 13% yield of EG by **9**, 100% yield of MG by **10**) and MB hydrogenation (100% yield of BA by **9**, 56% yield of BA by **10**). Previous results showed that the catalytic performances of complexes (Ph_2_P(CH_2_)_2_NH_2_)_2_RuCl_2_ (**7**) and (Ph_2_P(CH_2_)_3_NH_2_)_2_RuCl_2_ (**8**) with flexible N,P-ligand were similar to complex **3**, which was effective for the reduction of aromatic esters but low active for the reduction of oxalate esters^38^. Thus, combination of the rigid *o*-PPh_2_C_6_H_4_NH_2_ and soft PPh_2_CH_2_CH_2_NH_2_ or PPh_2_(CH_2_)_3_NH_2_ in complexes **9** and **10** significantly promotes the reaction efficiency. Further optimization of the structure of the ligands is under process.

In summary, we have demonstrated the first result of modulating nonbonding interactions for the selective hydrogenation of esters into alcohols. The newly-developed ruthenium complex **4** containing one CH_2_ linkage in the structure exhibits satisfactory performance in the hydrogenation of DMO and MB, whereas complexes **2** without CH_2_ and **3** with two CH_2_ linkage ligands show high catalytic activity only toward DMO or MB hydrogenation, respectively. The computational analyses and the corresponding comparative studies show that the CH_2_ linkage modulates the attractive nonbonding and steric interactions between the reactants, which dominate the accessibility of reactive configurations with the favorable orientation for the initial hydride transfer. These findings not only demonstrate the remarkably effects of nonbonding interactions on the selective hydrogenation of esters but also provide a new perspective on the mechanistic understanding of catalysis with uncommon reactivity and selectivity. The exceptional performance of complex **4** as well as of complexes **9** and **10** in catalytic hydrogenation may encourage further efforts to develop efficient catalyst systems through ligand combination.

## Methods

### Materials and general methods

All manipulations were carried out under a dry Ar or N_2_ atmosphere by using Schlenk line and glovebox techniques. The organic solvents toluene, *n*-hexane, tetrahydrofuran (THF) and diethyl ether were dried by refluxing with sodium/potassium benzophenone under N_2_ prior to use. CHCl_3_ and 1,4-dioxane were distilled from CaH_2_ and kept in the glovebox for use. The substrates employed in the catalytic reactions were purchased from Aldrich, J&K, or Alfa-Aesar Chemical Co. and used after purification according to the standard method. Complexes (*o*-Ph_2_PC_6_H_4_NMe_2_)RuCl_2_(PPh_3_)^46^, (Ph_2_PCH_2_CH_2_NH_2_)_2_RuCl_2_ (**7**)^47^, and (Ph_2_P(CH_2_)_3_NH_2_)_2_RuCl_2_ (**8**)^48^ were prepared according to the procedure reported in literatures. The NMR (^1^H, ^13^C{^1^H}, ^31^P{^1^H}) spectra were measured on a Bruker AVIII-500 spectrometer and the IR spectra were recorded on a Nicolet FT-IR 330 spectrometer. Elemental analysis was performed on a Thermo Quest Italia SPA EA 1110 instrument. X-ray crystal structure information is available at the Cambridge Crystallographic Data Centre (CCDC) under deposition numbers CCDC-1521753 (**4**), CCDC-1521754 (**5**), and CCDC-1521755 (**6**). See Supplementary Information for detailed experimental procedures, and crystallographic, spectroscopic and computational analyses.

### Catalytic reaction

The hydrogenation reactions were performed in a 100 mL Teflon-lined Parr stainless-steel autoclave. Generally, ruthenium complex, THF solvent, substrate, NaOMe, and *p*-xylene (50 μL, as internal standard) were charged into the lining in a glovebox. The autoclave was sealed and retrieved. Afterwards, the autoclave was purged through three successive cycles of pressurization/venting with H_2_ (10 bar) by maintaining at ca. 5 °C in an ice-water bath. The autoclave was pressurized with H_2_ (50 bar), closed, and placed in a temperature-controlled heating mantle. After the reaction was completed, the autoclave was quickly cooled to ca. 5 °C and depressurized. The solution was analyzed using GC (FULI company, 9790II) equipped with a KB-Wax column (60 m × 0.32 mm × 0.33 μm) or ^1^H NMR spectroscopy after purification by going through a short, silica-filled column. Nitrogen was used as carrier gas of GC analysis and the gas flow rate was 25 mL/min. The injector temperature was 260 °C, and the FID temperature was 250 °C. The oven temperature was started from 40 °C and kept for 5 min, then increased to 200 °C in 10 °C/min and kept for 20 min. The conversion of substrate and the yield of alcohol were calculated using *p*-xylene as an internal standard.

### Theoretical computation

All of the DFT calculations were carried out with the Gaussian 09 program. The molecular geometries of reactants, transition states, and intermediates were fully optimized by the B3LYP^49^ functional. Here the LanL2DZ basis set augmented with polarization functions (Ru(ζf) = 1.235 and P(ζd)^50^ = 0.340) was chosen to describe ruthenium and phosphorus atoms, and the basis set of 6–31 + G(d,p) was used for all other atoms. The solvent effect on the hydrogen transfer was evaluated by the SMD^51^ model with the experimentally used THF, and the total energies were calibrated by M06/6–311 ++ G(d,p)//B3LYP/6–31 + G(d,p) single-point calculations. For comparison, all the initial reactive conformers were re-optimized at the B3LYP-D3/6–31 G(d,p) level of theory^52^. The counterpoise correction scheme of Boys and Bernardi was applied to correct the basis-set superposition error. At the same time, a correction of **−**2.6 kcal/mol (temperature = 298.15 K) was applied to measure the free energy change based on the theory of free volume.

MD simulations were performed using the Forcite module encoded in the Material Studio software with the universal force field (UFF)^53^. A 35.9 × 35.9 × 35.9 Å^3^ cubic box with the periodic boundary conditions in all directions was constructed by the Amorphous Cell module (Fig. S20). In terms of the reaction conditions in experiment, the cubic box contains one catalyst and two hundred substrates with a density of 1.0 g/cm^3^. The annealing approach with 20-annealing cycle was adopted for the minimization of reaction system (temperature: 300–800 K, total time: 20 ps, Nose thermostat, NVE ensemble). Afterwards, MD simulations were carried out under the NPT ensemble at a constant temperature of 300 K using a Nose-Hoover thermostat. The equations of motion were integrated with the velocity Verlet algorithm for a total simulation time of 5 ns with a time step of 1 fs, and the cut-off value is 18.0 Å selected for the electrostatic and van der Waal summation method.

## Electronic supplementary material

Supplementary Information
