# Supplementary material for: CH2 Linkage Effects on the Reactivity of Bis(aminophosphine)–Ruthenium Complexes for Selective Hydrogenation of Esters into Alcohols
Source: Sci Rep. 2017 Jun 21;7:3961. doi: 10.1038/s41598-017-04362-9 (PMC5479805; doi:10.1038/s41598-017-04362-9)
Supplement: Supplementary file 1 — Supplementary Information [file 41598_2017_4362_MOESM1_ESM.pdf]

Supplementary Information

CH_2 Linkage Effects on the Reactivity of Bis(aminophosphine)–Ruthenium Complexes for Selective Hydrogenation of Esters into Alcohols

Xiaolong Fang,[1,+] Mingjun Sun,[2,+] Jianwei Zheng,[1] Bin Li,[1] Linmin Ye,[1] Xiaoping Wang,[1] Zexing Cao,[2,*] Hongping Zhu[1,*] and Youzhu Yuan[1,*]

[1] State Key Laboratory of Physical Chemistry of Solid Surfaces, National Engineering Laboratory for Green Chemical Productions of Alcohols–Ethers–Esters, *i*ChEM, College of Chemistry and Chemical Engineering, Xiamen University, Xiamen, 361005, China.

[2] State Key Laboratory of Physical Chemistry of Solid Surfaces, Fujian Provincial Key Laboratory of Theoretical and Computational Chemistry, College of Chemistry and Chemical Engineering, Xiamen University, Xiamen 361005, China.

[*]corresponding to: yzyuan@xmu.edu.cn

[+]these authors contributed equally to this work

Table of Contents:

**I.**	**General Experimental**	**2**
**II.**	**X-Ray Crystallographic Analysis**	**7**
**III.**	**Activity Tests**	**12**
**IV.**	**Theoretical Calculations**	**15**
**V.**	**NMR Spectra**	**30**
**VI.**	**Cartesian coordinates for the optimized structures**	**40**
**VII.**	**References**	**86**

I. General Experimental

Synthesis of complex (*o*-$PPh_2C_6H_4CH_2NH_2$)(*o*-$PPh_2C_6H_4NH_2$)$RuCl_2$ (4) A mixture of **1** (0.43 g, 0.3 mmol) and *o*-$PPh_2C_6H_4CH_2NH_2$ (0.18 g, 0.6 mmol) in toluene (40 mL) in a 100 mL Schlenk flask was stirred and allowed to heat to 100 ℃ for 48 h. The color of the mixture was gradually changed from orange to light yellow, during which **1** reacted with *o*-$PPh_2C_6H_4CH_2NH_2$ and precipitated complex **4**. After workup and by cooling to room temperature, the solid of **4** was collected by filtration and washed with *n*-hexane (2 mL). Yield: 0.38 g, 86%.

^{1}H NMR plus ^{1}H-^{13}C HSQC (500 MHz, $CDCl_3$, 298 k, ppm): δ 3.67 (br, 2 H, C*H₂*), 4.00 (br, 2 H, N*H₂*), 5.94 (br, 2 H, N*H₂*), 6.72 (t, J_{HH} = 10.0 Hz), 6.83 (t, J_{HH} = 10.0 Hz), 6.87 (s), 6.89 (s), 6.93 (t, J_{HH} = 5.0 Hz), 7.01 (t, J_{HH} = 7.5 Hz), 7.12 (t, J_{HH} = 7.5 Hz), 7.17–7.36 (m), 7.58 (t, J_{HH} = 10.0 Hz) (28 H, C_6H_4 and *Ph*).

^{13}C{^{1}H} NMR (125 MHz, $CDCl_3$, 298 k, ppm): δ 48.84 (d, J_{PC} = 10.0 Hz, 1 C, *C*H_2), 126.81 (d, J_{PC} = 5.0 Hz), 126.91 (d, J_{PC} = 2.5 Hz), 127.38 (dd, J_{PC} = 10.0, 15.0Hz), 128.46 (d, J_{PC} = 7.5 Hz), 129.17 (d, J_{PC} = 67.5 Hz), 129.78 (d, J_{PC} = 32.5 Hz), 130.66 (d, J_{PC} = 8.8 Hz), 131.91 (s), 133.27 (s), 133.78 (d, J_{PC} = 42.5 Hz), 134.14 (d, J_{PC} = 10.0 Hz), 134.93 (d, J_{PC} = 10.0 Hz), 135.72 (d, J_{PC} = 37.5 Hz), 136.60 (d, J_{PC} = 42.5 Hz), 139.29 (d, J_{PC} = 41.3 Hz), 140.48 (d, J_{PC} = 13.8 Hz), 147.88 (d, J_{PC} = 18.8 Hz) (36 C, C_6H_4 and *Ph*).

^{31}P{^{1}H} NMR (202 MHz, $CDCl_3$, 298 k, ppm): δ 49.90 (d, $^2J_{PP}$ = 30.3 Hz), 57.32 (d, $^2J_{PP}$ = 30.3 Hz).

IR (Nujol, KBr, cm^{-1}): 3206, 3226, 3285 (N–H stretching).

Analysis (calcd., found for $RuCl_2C_{37}H_{34}N_2P_2$): C (60.00, 59.63), N (3.78, 3.61), H (4.63, 4.75).

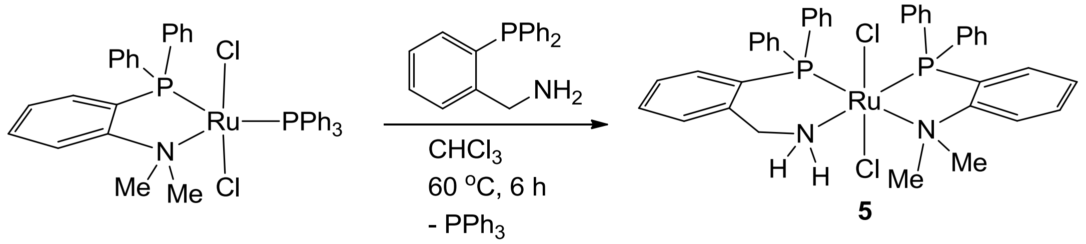

Synthesis of complex (*o*-$PPh_2C_6H_4CH_2NH_2$)(*o*-$PPh_2C_6H_4NMe_2$)$RuCl_2$ (5) A solution of (*o*-$PPh_2C_6H_4NMe_2$)$RuCl_2(PPh_3)$ (0.22 g, 0.3 mmol) and *o*-$PPh_2C_6H_4CH_2NH_2$ (87.0 mg, 0.3 mmol) in $CHCl_3$ (20 mL) in a 50 mL Schlenk flask was stirred and allowed to heat to 60 ℃ for 6 h, during which an orange-red solution was obtained. After workup and by cooling to room temperature, the solution was concentrated to ca. 1 mL, and to it *n*-hexane (5 mL) was added. A light brick-red precipitate of **5** was quickly formed, which was collected and washed with *n*-hexane (2 mL). Yield: 0.20 g, 85%.

^{1}H NMR plus ^{1}H-^{13}C HSQC (500 MHz, $CDCl_3$, 298 k, ppm): δ 3.09 (s, 6 H, N*Me₂*), 3.95 (br, 2 H, NH_2), 4.08 (br, 2 H, CH_2), 6.63 (t, J_{HH} = 7.5 Hz), 6.72 (t, J_{HH} = 7.5 Hz), 6.88 (m), 7.13–7.37 (m), 7.44 (t, J_{HH} = 10.0 Hz), 7.58 (m) (28 H, C_6H_4 and *Ph*).

^{13}C{^{1}H} NMR (125 MHz, $CDCl_3$, 298 k, ppm): δ 48.78 (d, J_{PC} = 10.0 Hz, 2 C, CH_3), 53.76 (s, 1 C, CH_2), 119.15 (d, J_{PC} = 8.8 Hz), 126.07 (d, J_{PC} = 5.0 Hz), 127.33 (dd, J_{PC} = 6.3, 8.8 Hz), 128.32 (d, J_{PC} = 7.5 Hz), 128.63 (d, J_{PC} = 6.3 Hz), 128.85 (s), 129.36 (dd, J_{PC} = 1.3, 38.8 Hz), 129.69 (d, J_{PC} = 1.3 Hz), 130.47 (d, J_{PC} = 7.5 Hz), 131.21 (d, J_{PC} = 130.0 Hz), 133.16 (d, J_{PC} = 46.3 Hz), 133.31 (s), 133.91 (d, J_{PC} = 18.8 Hz), 134.92 (d, J_{PC} = 10.0 Hz), 135.15 (d, J_{PC} = 8.8 Hz), 136.49 (d, J_{PC} = 41.3 Hz), 137.88 (d, J_{PC} = 42.5 Hz), 138.31 (d, J_{PC} = 38.8 Hz), 13 9.61 (d, J_{PC} = 13.8 Hz), 161.68 (d, J_{PC} = 16.3 Hz) (36 C, C_6H_4 and *Ph*).

^{31}P{^{1}H} NMR (202 MHz, $CDCl_3$, 298 k, ppm): δ 50.57 (d, $^{2}J_{PP}$ = 34.3 Hz), 51.95 (d, $^{2}J_{PP}$ = 34.3 Hz).

IR (Nujol, KBr, cm^{-1}): 3223, 3296 (N–H stretching).

Analysis (calcd., found for $RuCl_2C_{39}H_{38}N_2P_2$): C (60.93, 61.09), N (3.65, 3.76), H (4.98, 4.82).

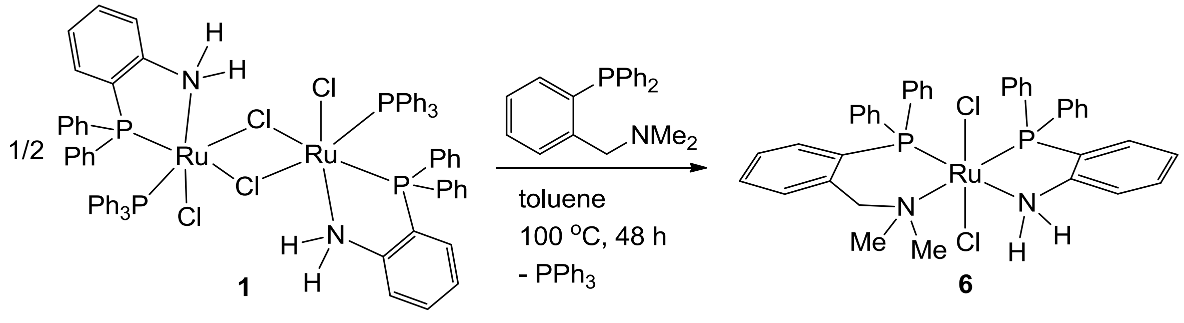

Synthesis of complex (*o*-$PPh_2C_6H_4CH_2NMe_2$)(*o*-$PPh_2C_6H_4NH_2$)$RuCl_2$ (6) A mixture of **1** (0.28 g, 0.2 mmol) and *o*-$PPh_2C_6H_4CH_2NMe_2$ (0.13 g, 0.4mmol) in toluene (30 mL) in a 100 mL Schlenk flask was stirred and allowed to heat to 100 ℃ for 48 h. The color of the mixture was gradually changed from orange to brick-red, during which **1** reacted with *o*-$PPh_2C_6H_4CH_2NMe_2$ and precipitated complex **6**. After workup and by cooling to room temperature, the solid of **6** was collected by filtration and washed with *n*-hexane (2 mL). Yield: 0.27 g, 88%.

^{1}H NMR plus ^{1}H-^{13}C HSQC (500 MHz, $CDCl_3$, 298 k, ppm): δ 2.61 (s, 6 H, N*Me₂*), 3.84 (br, 2 H, CH_2), 5.83 (br, 2 H, NH_2), 6.75 (t, J_{HH} = 7.5 Hz), 6.91 (t, J_{HH} = 7.5 Hz), 7.00–7.35 (m), 7.55 (m) (28 H, C_6H_4 and *Ph*).

$^{13}C\{^{1}H\}$ NMR (125 MHz, $CDCl_3$, 298 k, ppm): δ 52.53 (s, 2 C, CH_3), 70.25 (d, J_{PC} = 11.3 Hz, 1 C, CH_2), 126.92 (dd, J_{PC} = 2.5, 8.8 Hz), 127.11 (s), 127.21 (dd, J_{PC} = 10, 27.5 Hz), 128.58 (d, J_{PC} = 5.0 Hz), 128.75 (d, J_{PC} = 1.3 Hz), 129.41 (d, J_{PC} = 1.3 Hz), 130.44 (s), 132.00 (s), 133.07 (d, J_{PC} = 8.8 Hz), 133.94 (s), 134.81 (dd, J_{PC} = 8.8, 51.3 Hz), 136.20 (d, J_{PC} = 37.5 Hz), 138.98 (d, J_{PC} = 43.8 Hz), 139.33 (d, J_{PC} = 15.0 Hz), 147.04 (d, J_{PC} = 18.8 Hz) (36 C, C_6H_4 and *Ph*).

$^{31}P\{^{1}H\}$ NMR (202 MHz, $CDCl_3$, 298 k, ppm): δ 45.62 (d, $^{2}J_{PP}$ = 36.4 Hz), 58.65 (d, $^{2}J_{PP}$ = 36.4 Hz).

IR (Nujol, KBr, cm^{-1}): 3091, 3128, 3164, 3285 (N–H stretching).

Analysis (calcd., found for $RuCl_2C_{39}H_{38}N_2P_2$): C (60.93, 60.62), N (3.65, 3.48), H (4.98, 5.06).

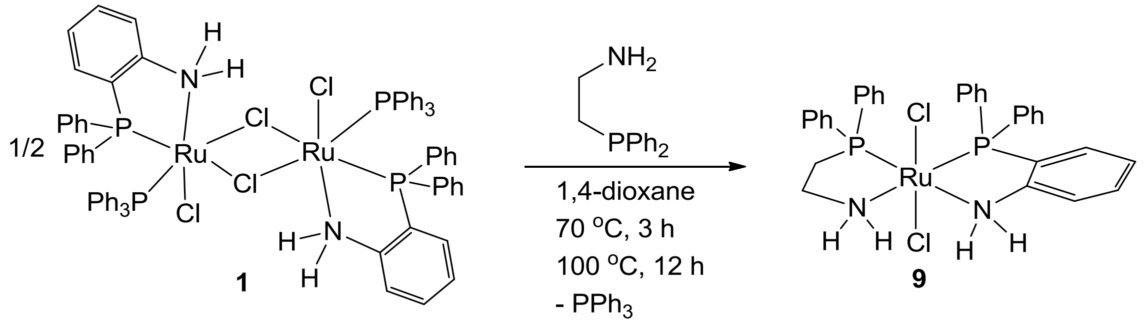

Synthesis of complex ($Ph_2PCH_2CH_2NH_2$)(*o*-$PPh_2C_6H_4NH_2$)$RuCl_2$ (9) A mixture of **1** (0.21 g, 0.15 mmol) and $Ph_2PCH_2CH_2NH_2$ (68.8 mg, 0.3 mmol) in 1,4-dioxane (30 mL) in a 100 mL Schlenk flask was stirred and allowed to heat to 70 °C for 3 h, during which a yellow solution was developed. Then, the temperature was increased to 100 °C and kept at this temperature overnight. After workup and by cooling to room temperature, the solution was filtrated and the filtrate was concentrated to ca. 3 mL, and to it *n*-hexane (6 mL) was added. A light yellow precipitate of **9** was quickly formed, which was collected and washed with *n*-hexane (2 mL). Yield: 0.16 g, 78%.

^{1}H NMR plus ^{1}H-^{13}C HSQC (500 MHz, $CDCl_3$, 298 k, ppm): δ 2.69 (m, 2 H, CH_2), 3.15 (m, 2 H, CH_2), 4.07 (s, 2 H, NH_2), 5.91 (s, 2 H, NH_2), 7.07–7.28 (m), 7.53 (t, J_{HH} = 7.5 Hz), (24 H, C_6H_4 and *Ph*).

^{13}C{^{1}H} NMR (125 MHz, $CDCl_3$, 298 k, ppm): δ 33.10 (d, J_{PC} = 23.8 Hz, 1 C, CH_2), 41.26 (d, J_{PC} = 3.8 Hz, 1 C, CH_2), 126.45 (d, J_{PC} = 3.8 Hz), 126.82 (d, J_{PC} = 7.6 Hz), 127.47 (dd, J_{PC} = 15, 8.8 Hz), 129.06 (d, J_{PC} = 17.1 Hz), 133.53 (s), 133.60 (s), 133.86 (dd, J_{PC} = 19.1, 9.5 Hz), 136.14 (dd, J_{PC} = 46.1, 41.3 Hz), 137.84 (d, J_{PC} = 36.3 Hz), 148.96 (d, J_{PC} = 19.0 Hz) (30 C, C_6H_4 and *Ph*).

^{31}P{^{1}H} NMR (202 MHz, $CDCl_3$, 298 k, ppm): δ 60.97 (d, $^2J_{PP}$ = 30.3 Hz), 63.00 (d, $^2J_{PP}$ = 30.3 Hz).

IR (Nujol, KBr, cm^{-1}): 3105, 3130, 3178, 3327 (N–H stretching).

Analysis (calcd., found for $RuCl_2C_{32}H_{32}N_2P_2$): C (56.64, 56.76), N (4.13, 4.19), H (4.75, 4.63).

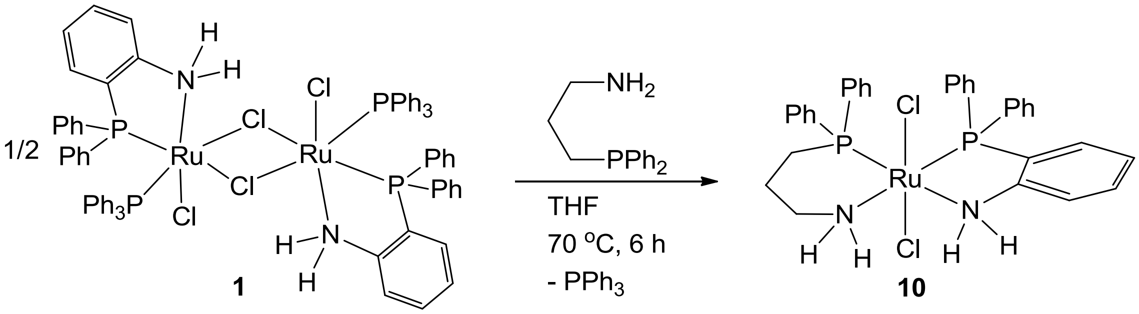

Synthesis of complex [$Ph_2P(CH_2)_3NH_2$](*o*-$PPh_2C_6H_4NH_2$)$RuCl_2$ (10) A mixture of **1** (0.28 g, 0.2 mmol) and $Ph_2P(CH_2)_3NH_2$ (97.3 mg, 0.4 mmol) in THF (30 mL) in a 100 mL Schlenk flask was stirred and allowed to heat to 70 ℃ for 6 h, during which a crimson solution was first formed then changed to light yellow. After workup and by cooling to room temperature, the solution was filtrated and the filtrate was concentrated to ca. 3 mL, and to it *n*-hexane (6 mL) was added. A light yellow precipitate of **10** was quickly formed, which was collected and washed with *n*-hexane (2 mL). Yield: 0.23 g, 82%.

^{1}H NMR plus ^{1}H-^{13}C HSQC (500 MHz, $CDCl_3$, 298 k, ppm): δ 2.03 (m, 2 H, C*H2)*, 2.75 (m, 2 H, C*H2)*, 3.18 (br, 2 H, C*H2)*, 3.40 (br, 2 H, N*H2)*, 5.80 (s, 2 H, N*H2)*, 7.04–7.28 (m) (24 H, C_6H_4 and *Ph*).

^{13}C{^{1}H} NMR (125 MHz, $CDCl_3$, 298 k, ppm): δ 24.90 (s, 1 C, *C*H_2), 25.52 (d, J_{PC} = 26.3 Hz, 1 C, *C*H_2), 41.20 (s, 1 C, *C*H_2), 126.42 (d, J_{PC} = 10.0 Hz), 126.57 (d, J_{PC} = 5.0 Hz), 127.39 (dd, J_{PC} = 20.0, 8.8 Hz), 128.62 (s), 128.77 (d, J_{PC} = 23.8 Hz), 128.95 (d, J_{PC} = 8.8 Hz), 130.22 (s), 133.32 (s), 133.59 (d, J_{PC} = 8.8 Hz), 133.91 (d, J_{PC} = 20.0 Hz), 134.14 (d, J_{PC} = 10.0 Hz), 135.73 (d, J_{PC} = 42.5 Hz), 137.47 (d, J_{PC} = 40.0 Hz), 139.34 (d, J_{PC} = 38.8 Hz), 148.22 (d, J_{PC} = 17.5 Hz) (30 C, C_6H_4 and *Ph*).

^{31}P{^{1}H} NMR (202 MHz, $CDCl_3$, 298 k, ppm): δ 34.93 (d, $^2J_{PP}$ = 32.3 Hz), 59.37 (d, $^2J_{PP}$ = 32.3 Hz).

IR (Nujol, KBr, cm^{-1}): 3125, 3226 (N–H stretching).

Analysis (calcd., found for $RuCl_2C_{33}H_{34}N_2P_2$): C (57.22, 56.92), N (4.05, 4.19), H (4.95, 5.08).

II. X-ray Crystallographic Analysis

X-ray Crystallographic Analysis of 4: The crystallographic data for **4**·$CDCl_3$ was collected at 173 K on a Rigaku R-Axis Spider IP system using graphite-monochromated Mo-Ka radiation (λ = 0.71073 Å). Intensity measurements were performed on a rapidly cooled crystal with dimensions of 0.40 × 0.17 × 0.16 mm^3 in the range 3.23 °< θ < 27.48 °. The data completeness collected was 98.4%. Absorption correction was applied using the spherical harmonic program (multi-scan type). The structure was solved by direct method (SHELXS-96)[1] and refined against F^2 using SHELXL-97 program[2]. In general, non-hydrogen atoms were located from different Fourier synthesis and refined anisotropically, and hydrogen atoms were included using a riding mode with U_{iso} tied to the U_{iso} of the parent atom unless otherwise specified. The $CHCl_3$ solvent molecule was disordered and treated in two parts that were refined into the respective occupations of 0.77850 and 0.22150. The hydrogen atoms at the nitrogen atoms (H(11) and H(12) at the N(1) and H(21) and H(22) at the N(2)) were located from different Fourier synthesis and refined isotropically. Crystal data for **4**·$CDCl_3$: $C_{38}H_{35}Cl_5N_2P_2Ru$, M_r = 859.94, triclinic, space group P-1, a = 11.106(2), b = 12.117(2), c = 14.555(3) Å, α = 91.62(3) °, β = 109.37(3) °, γ = 90.74(3) °, V = 1,846.7(6) Å^3, Z = 2, ρ_{calcd} = 1.546 g/cm^3, $\mu(Mo_{K\alpha})$ = 0.904 mm^{-1}, F(000) = 872; 18,124 measured reflections, 8,352 independent (R_{int} = 0.0288). The final refinements converged at R_1 = 0.0314 and wR_2 = 0.0605 for $I > 2\sigma(I)$ and R_1 = 0.0453 and wR_2 = 0.0805 for all data. The goodness of fit (GOF) is 1.100. Fourier synthesis gave a min/max residual electron density –0.930/0.784 e Å^3. CCDC-1521753 contains the supplementary crystallographic data. The data can be obtained free of charge from the Cambridge Crystallographic Data Centre via www.ccdc.cam.ac.uk/data_request/cif.

X-ray Crystallographic Analysis of 5: The crystallographic data for **5**·1.5 C_7H_8 was collected at 173 K on an Agilent Super Nova system using Cu-Ka radiation (λ = 1.54178 Å). Intensity measurements were performed on a rapidly cooled crystal with dimensions of 0.30 × 0.20 × 0.20 mm^3 in the range 3.94 °< θ < 64.12 °. The data completeness collected was 97.0%. Absorption correction was applied using the spherical harmonic program (multi-scan type). The structure was solved by direct method (SHELXS-96)[1] and refined against F^2 using SHELXL-97 program[2]. In general, non-hydrogen atoms were located from different Fourier synthesis and refined anisotropically, and hydrogen atoms were included using a riding mode with U_{iso} tied to the U_{iso} of the parent atom unless otherwise specified. The hydrogen atoms H(1) and H(2) at the N(2) were located from different Fourier synthesis and refined isotropically. One toluene molecule was seriously disordered in which five carbon atoms were

determined and refined isotropically with the respective occupations of 1.00, 1.00, 0.50, 0.50, and 0.50. The hydrogen atoms for this toluene were not able to be located. Crystal data for **5**·1.5 C_7H_8: $C_{49.5}H_{46}Cl_2N_2P_2Ru$, M_r = 902.79, triclinic, space group *P*-1, *a* = 11.5442(4), *b* = 13.0610(4), *c* = 16.0258(6) Å, α = 106.489(3)°, β = 92.253(3)°, γ = 114.549(3)°, *V* = 2,073.01(12) $Å^3$, *Z* = 2, ρ_{calcd} = 1.446 g/cm^3, μ(Cu$_{K\alpha}$) = 5.264 mm^{-1}, *F*(000) = 930; 12,304 measured reflections, 6,700 independent (R_{int} = 0.0109). The final refinements converged at R_1 = 0.0284 and wR_2 = 0.0677 for $I > 2\sigma(I)$ and R_1 = 0.0286 and wR_2 = 0.0678 for all data. The goodness of fit (GOF) is 1.017. Fourier synthesis gave a min/max residual electron density –0.776/1.490 e $Å^3$. CCDC-1521754 contains the supplementary crystallographic data. The data can be obtained free of charge from the Cambridge Crystallographic Data Centre via www.ccdc.cam.ac.uk/data_request/cif.

X-ray Crystallographic Analysis of 6: The crystallographic data for **6** was collected at 173 K on an Oxford Gemini S Ultra system using Mo-Ka radiation (λ = 0.71073 Å). Intensity measurements were performed on a rapidly cooled crystal with dimensions of 0.30 × 0.10 × 0.10 mm^3 in the range 2.95° < θ < 26.00°. The data completeness collected was 99.8%. Absorption correction was applied using the spherical harmonic program (multi-scan type). The structure was solved by direct method (SHELXS-96)[1] and refined against F^2 using SHELXL-97 program[2]. In general, non-hydrogen atoms were located from different Fourier synthesis and refined anisotropically, and hydrogen atoms were included using a riding mode with U_{iso} tied to the U_{iso} of the parent atom unless otherwise specified. The hydrogen atoms H(1) and H(2) at the N(1) were located from different Fourier synthesis and refined isotropically. Crystal data for **6**: $C_{39}H_{38}Cl_2N_2P_2Ru$, M_r = 768.62, monoclinic, space group *P*2(1)/*n*, *a* = 15.7293(4), *b* = 13.7041(4), *c* = 16.3028(4) Å, β = 101.097(3)°, *V* = 3,448.46(16) $Å^3$, *Z* = 4, ρ_{calcd} = 1.480 g/cm^3, μ(Mo$_{K\alpha}$) = 0.734 mm^{-1}, *F*(000) = 1576; 16,558 measured reflections, 6,769 independent (R_{int} = 0.0539). The final refinements converged at R_1 = 0.0475 and wR_2 = 0.0869 for $I > 2\sigma(I)$ and R_1 = 0.0685 and wR_2 = 0.0935 for all data. The goodness of fit (GOF) is 1.050. Fourier synthesis gave a min/max residual electron density –0.512/0.725 e $Å^3$. CCDC-1521755 contains the supplementary crystallographic data. The data can be obtained free of charge from the Cambridge Crystallographic Data Centre via www.ccdc.cam.ac.uk/data_request/cif.

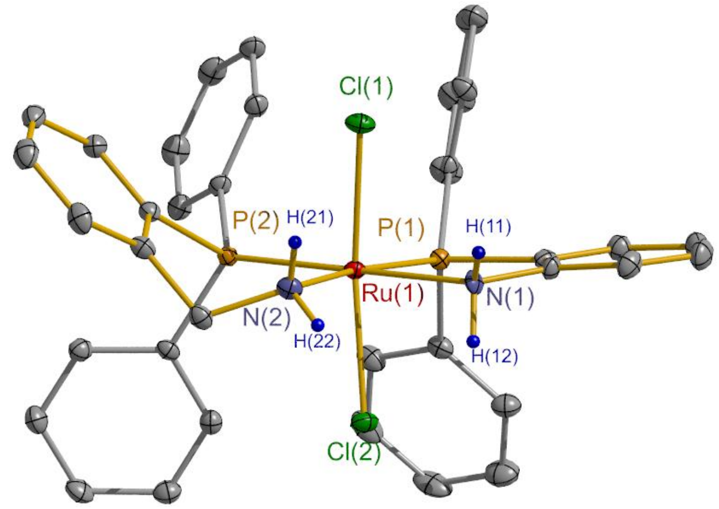

Figure S1. X-ray molecular structure of complex **4** with thermal ellipsoids at 30% probability level. Hydrogen atoms in aryl ring and methylene group have been omitted for clarity. Selected bond lengths [Å] and angles [°] for **4**: Ru(1)–P(1) 2.2650(12), Ru(1)–P(2) 2.2763(10), Ru(1)–N(1) 2.176(2), Ru(1)−N(2) 2.187(3), Ru(1)−Cl(1) 2.4271(10), Ru(1)−Cl(2) 2.4221(10); P(1)–Ru(1)–N(1) 83.06(7), P(2)–Ru(1)–N(2) 89.67(8). Estimated standard deviations are given in parenthesis.

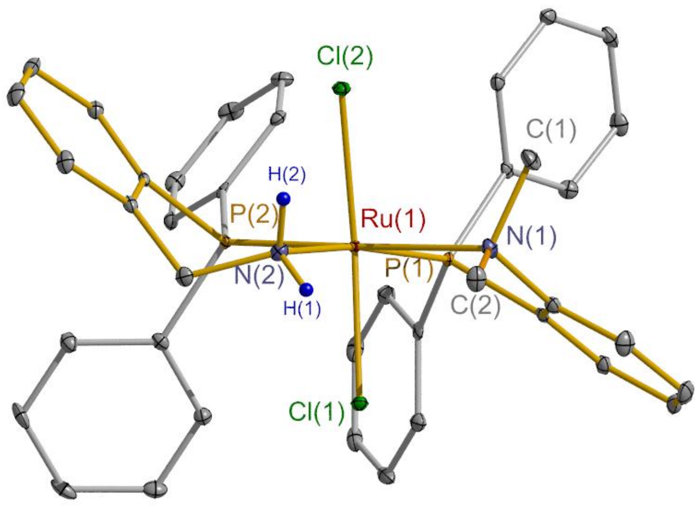

Figure S2. X-ray molecular structure of complex **5** with thermal ellipsoids at 30% probability level. In addition to amino hydrogen, other hydrogen atoms have been omitted for clarity. Selected bond lengths [Å] and angles [°] for **5**: Ru(1)–P(1) 2.2695(6), Ru(1)–P(2) 2.2656(6), Ru(1)–N(1) 2.334(2), Ru(1)−N(2) 2.194(2), Ru(1)−Cl(1) 2.4106(6), Ru(1)−Cl(2) 2.4319(6); P(1)–Ru(1)–N(1) 79.39(5), P(2)–Ru(1)–N(2) 88.38(6). Estimated standard deviations are given in parenthesis.

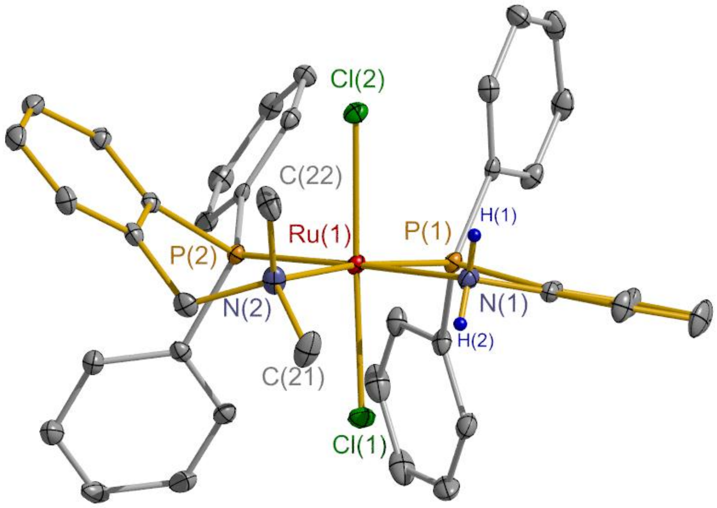

Figure S3. X-ray molecular structure of complex **6** with thermal ellipsoids at 30% probability level. In addition to amino hydrogen, other hydrogen atoms have been omitted for clarity. Selected bond lengths [Å] and angles [°] for **6**: Ru(1)–P(1) 2.2651(10), Ru(1)–P(2) 2.2828(9), Ru(1)–N(1) 2.171(3), Ru(1)−N(2) 2.373(3), Ru(1)−Cl(1) 2.4041(9), Ru(1)−Cl(2) 2.4306(9); P(1)–Ru(1)–N(1) 82.84(9), P(2)–Ru(1)–N(2) 91.18(8). Estimated standard deviations are given in parenthesis.

III. Activity Tests

Table S1. Control experiments without addition of complex **4** or NaOMe in hydrogenation of DMO to MG (or MB to BA).

Entry	Substrate	Ru/mol%	NaOMe/mol%	Conv./%	Yield/%
1	DMO	0.5	0	0	0
2[a]	DMO	0	5	3	0
3	MB	0.5	0	0	0
4	MB	0	10	0	0

Reaction conditions: 7.57 mmol ester, 10 mL THF, 50 bar H_2, 100 ℃, 4 h. [a] Decarbonylation occurred in this reaction.

Table S2. Hydrogenation of several lactones to alcohols with **4** under different H_2 pressures.

Entry	Substrate	$P_{(H2)}$/bar	Time/h	Conv./%	Yield/%
1	**E1**	20	12	93	93
2	**E1**	30	10	95	95
3	**E2**	20	12	76	76
4	**E3**	20	12	58	58
5	**E4**	20	12	95	95
6	**E5**	20	12	93	93
7	**E6**	20	12	86	73

Reaction conditions: 7.57 mmol ester, 0.1 mol% ruthenium, the molar ratio of NaOMe to ruthenium was 20, 10 mL THF, 100 ℃.

Table S3. Hydrogenation of benzaldehyde to benzyl alcohol catalyzed by ruthenium complexes **2** and **3**.

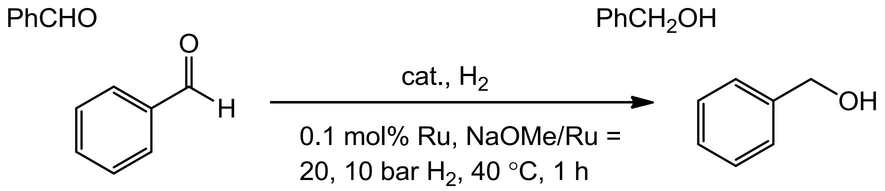

Entry	Ru complex	PhCHO Conv./%	$PhCH_2OH$ Yield/%
1	**2**	100	99
2	**3**	100	99

Reaction conditions:, 7.57 mmol PhCHO, 0.1 mol% ruthenium, 2 mol% NaOMe, 10 mL THF, 40 ℃; 10 bar H_2, 1 h.

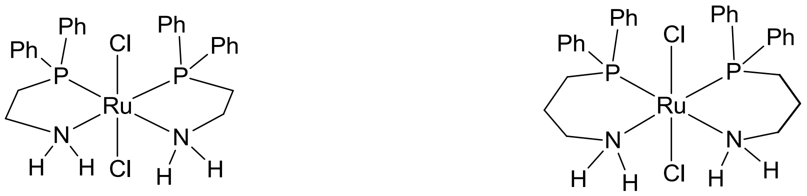

7

8

Figure S4. Structures of ruthenium complexes **7** and **8**.

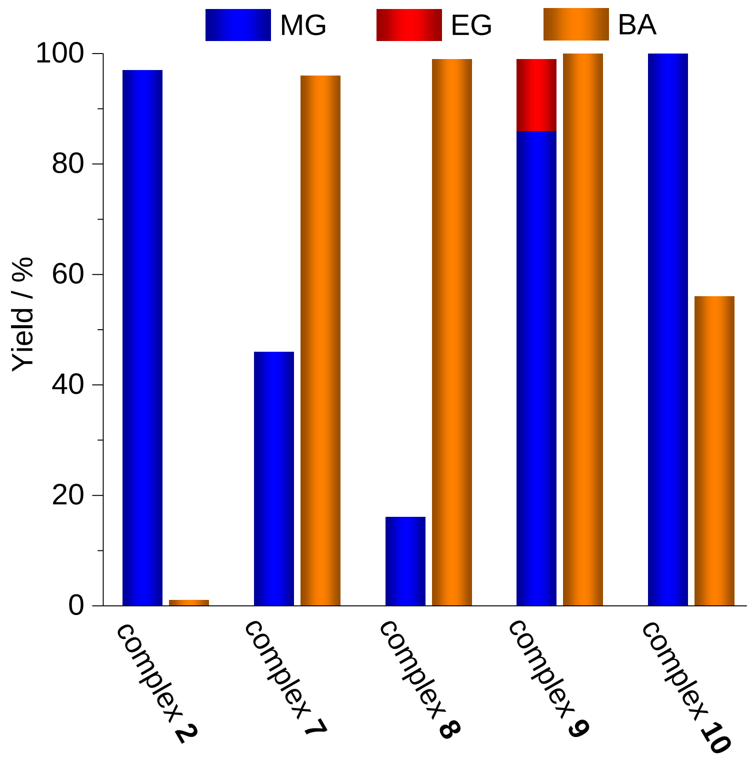

Figure S5. Catalytic performance of complexes **2**, and **7**–**10** for hydrogenation of DMO into MG (and/or EG) and that of MB into BA. Reaction conditions see Fig. 5.

IV. Theoretical Calculations

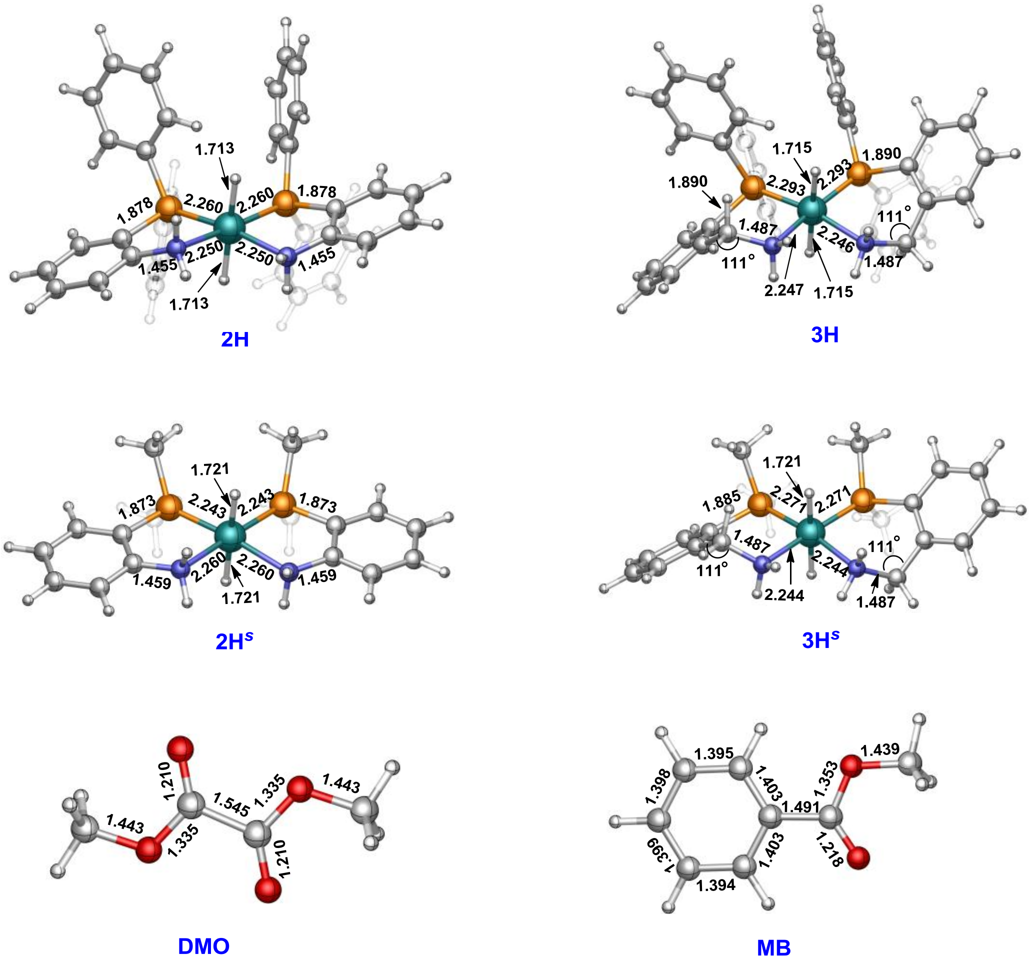

Figure S6. The optimized structures of the ruthenium catalysts and esters DMO and MB. **2H** and **3H**: real catalytically active species of **2** and **3**; **2H**^{s} and **3H**^{s}: modified models of **2** and **3** (Ph groups of **2H** and **3H** are replaced by Me groups).

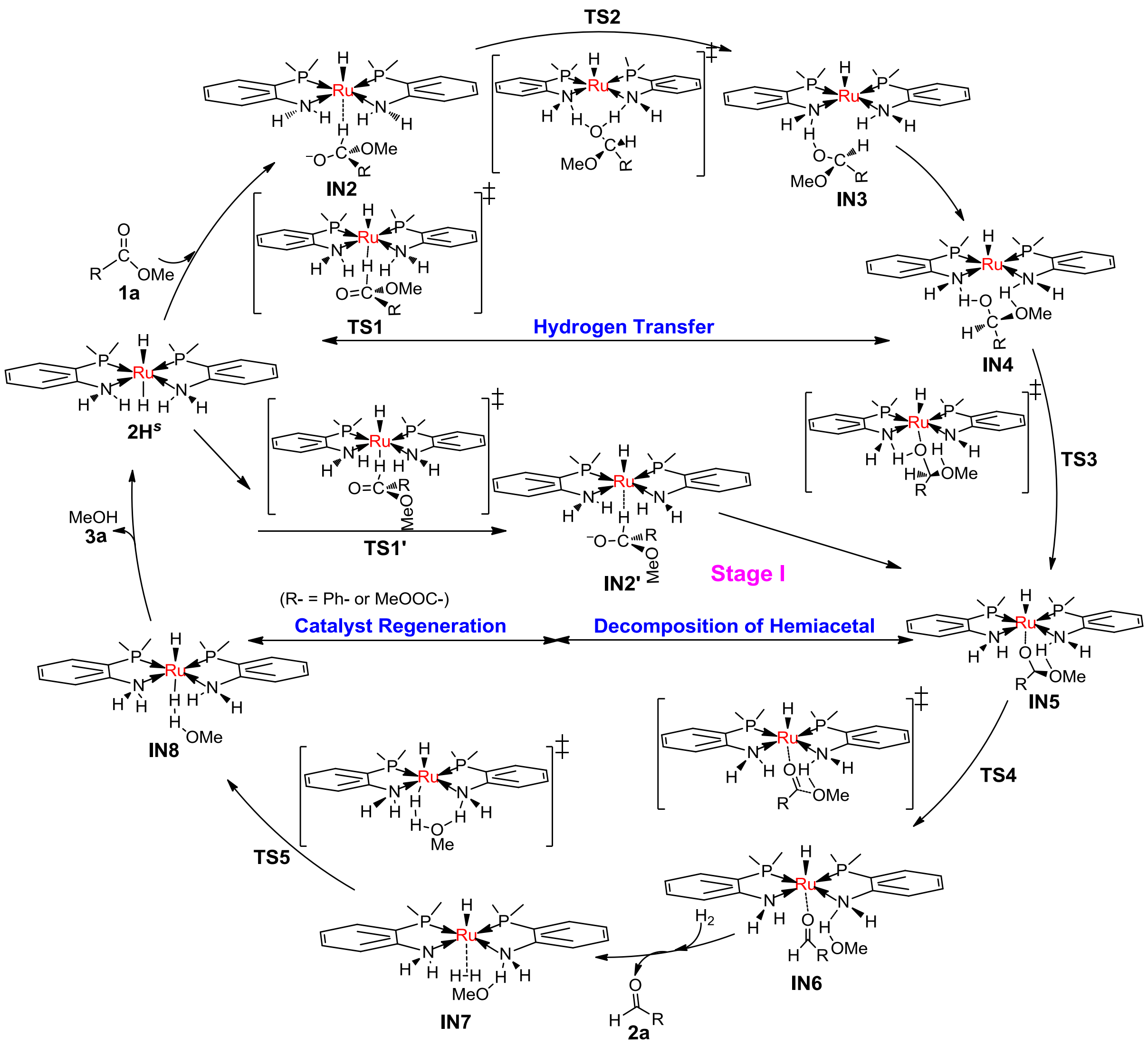

Figure S7. The detailed mechanism for stage I.

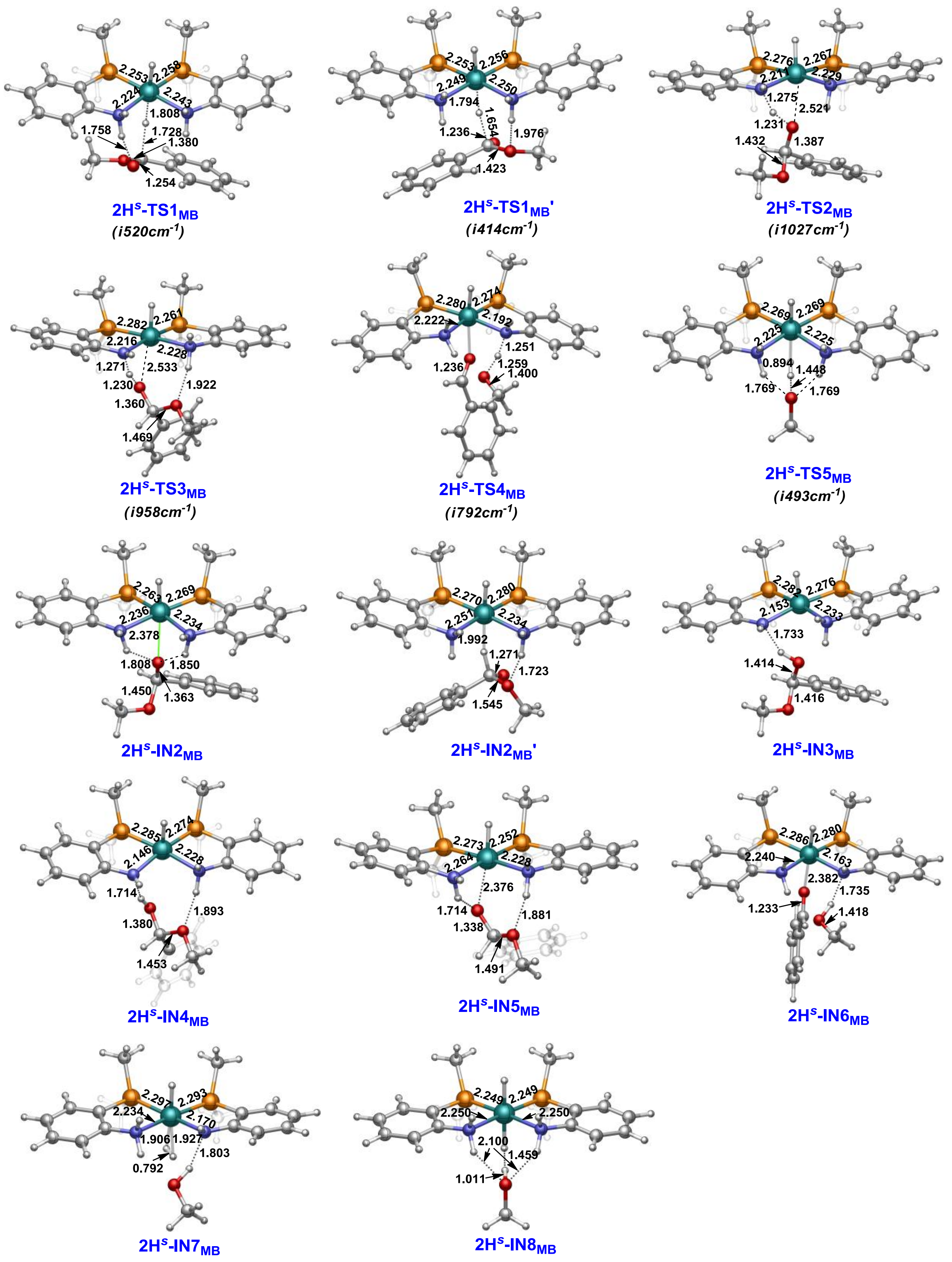

Figure S8. The optimized geometries of the transition states and intermediates for **2H**^{s} catalyzed hydrogenation of MB to benzaldehyde and methanol. Here, two possible reactive configurations with the outward or inward carbonyl (refer to **2H**^{s}-TS1$_{MB}$ and **2H**^{s}-TS1$_{MB}'$) for MB have been considered, and the results showed that the initial hydride transfer to the outward carbonyl is more favorable, which experiences a relatively low free energy barrier.

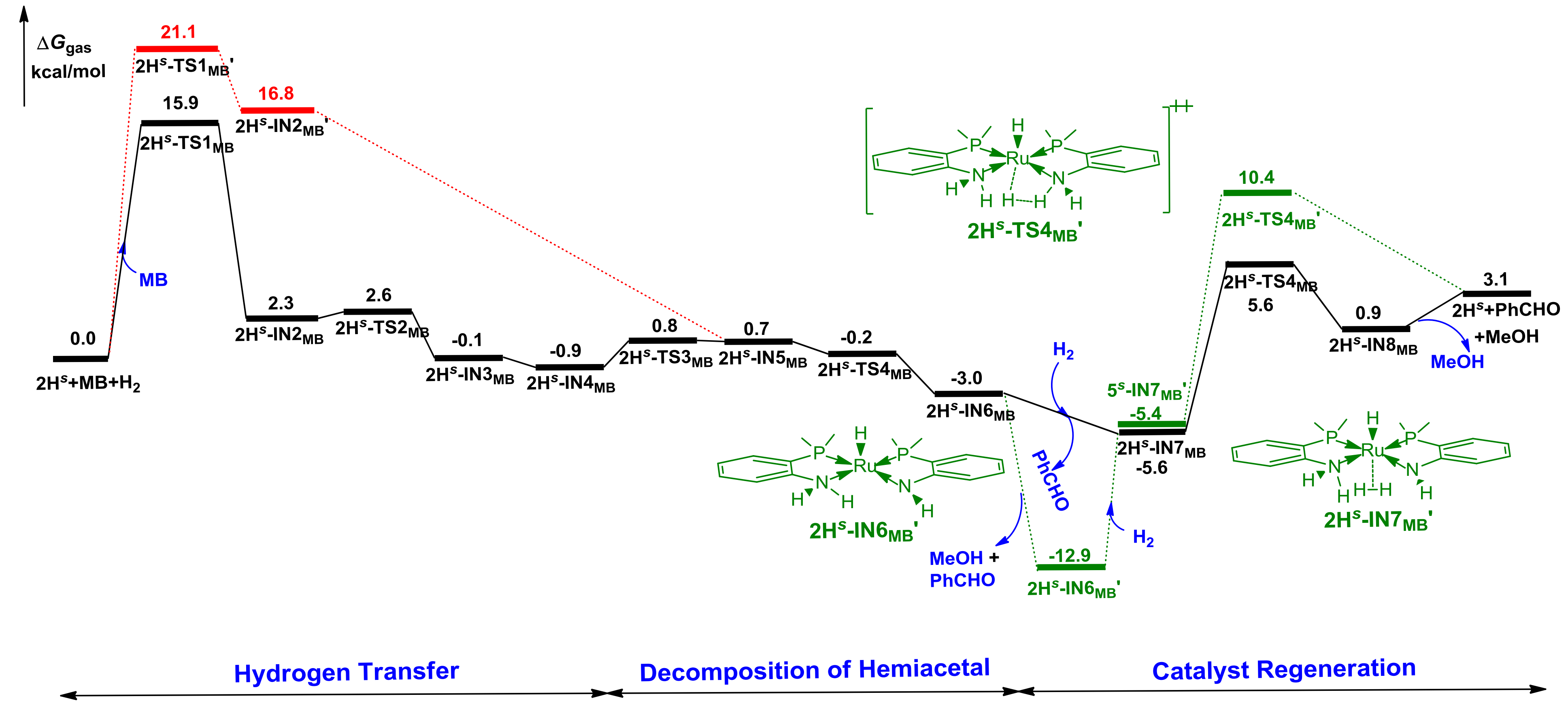

Figure S9. Free-energy profiles for **2H**^{s} catalyzed hydrogenation of MB into benzaldehyde and methanol.

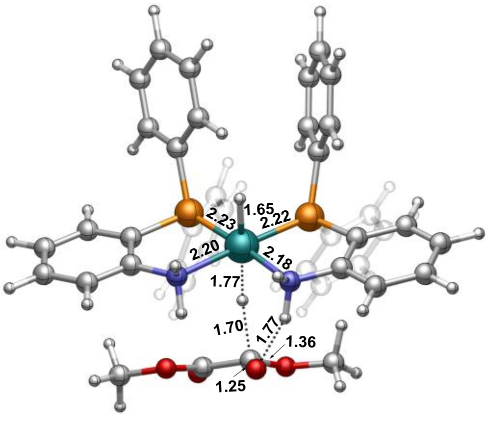

2H-TS1$_{DMO}$

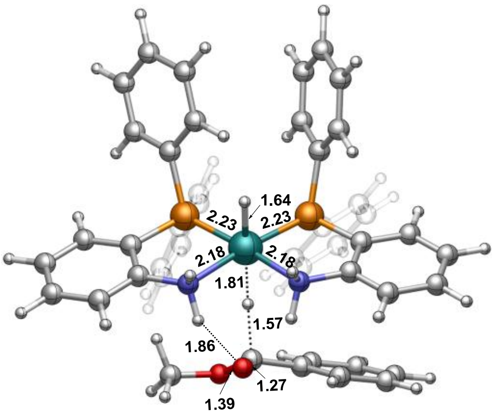

2H-TS1$_{MB}$

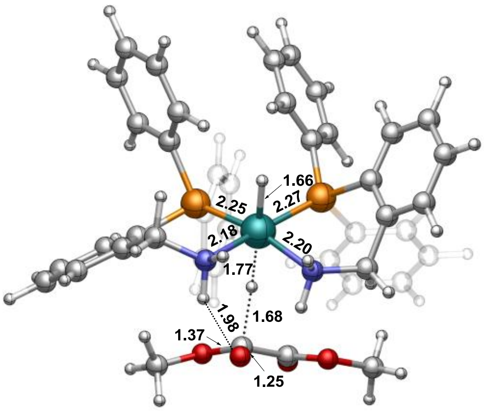

3H-TS1$_{DMO}$

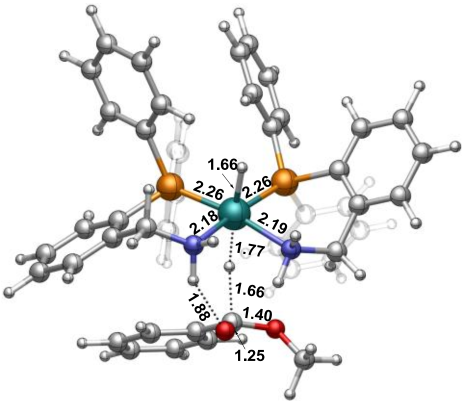

3H-TS1$_{MB}$

Figure S10. The optimized structures of TS1.

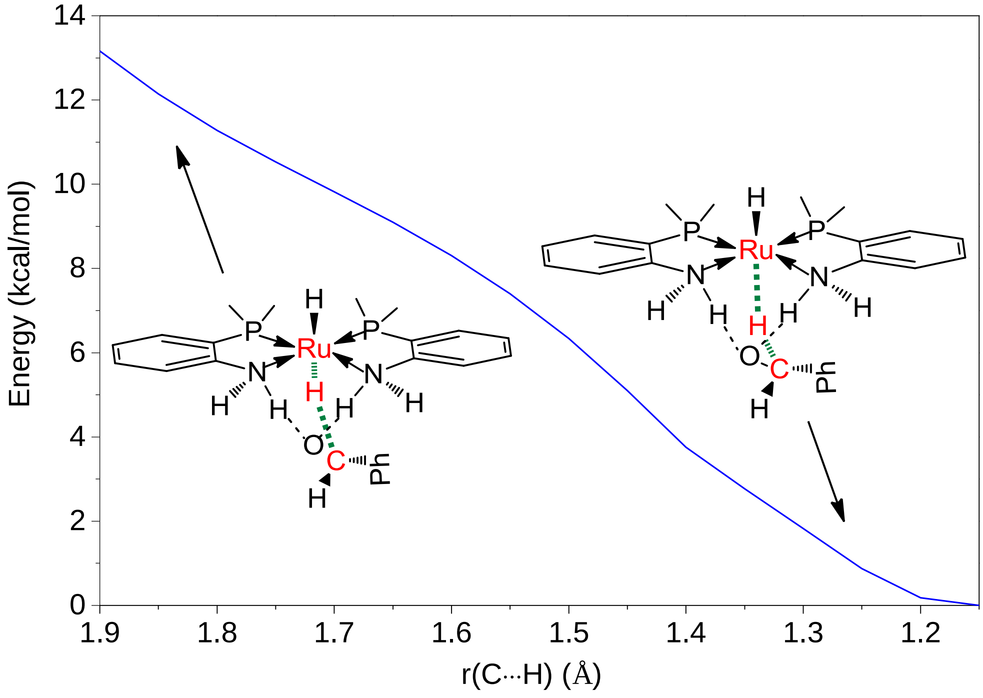

Figure S11. The flexible scanning for the ruthenium hydride transfer (Ru–H→C=O) in **2H**^{s}.

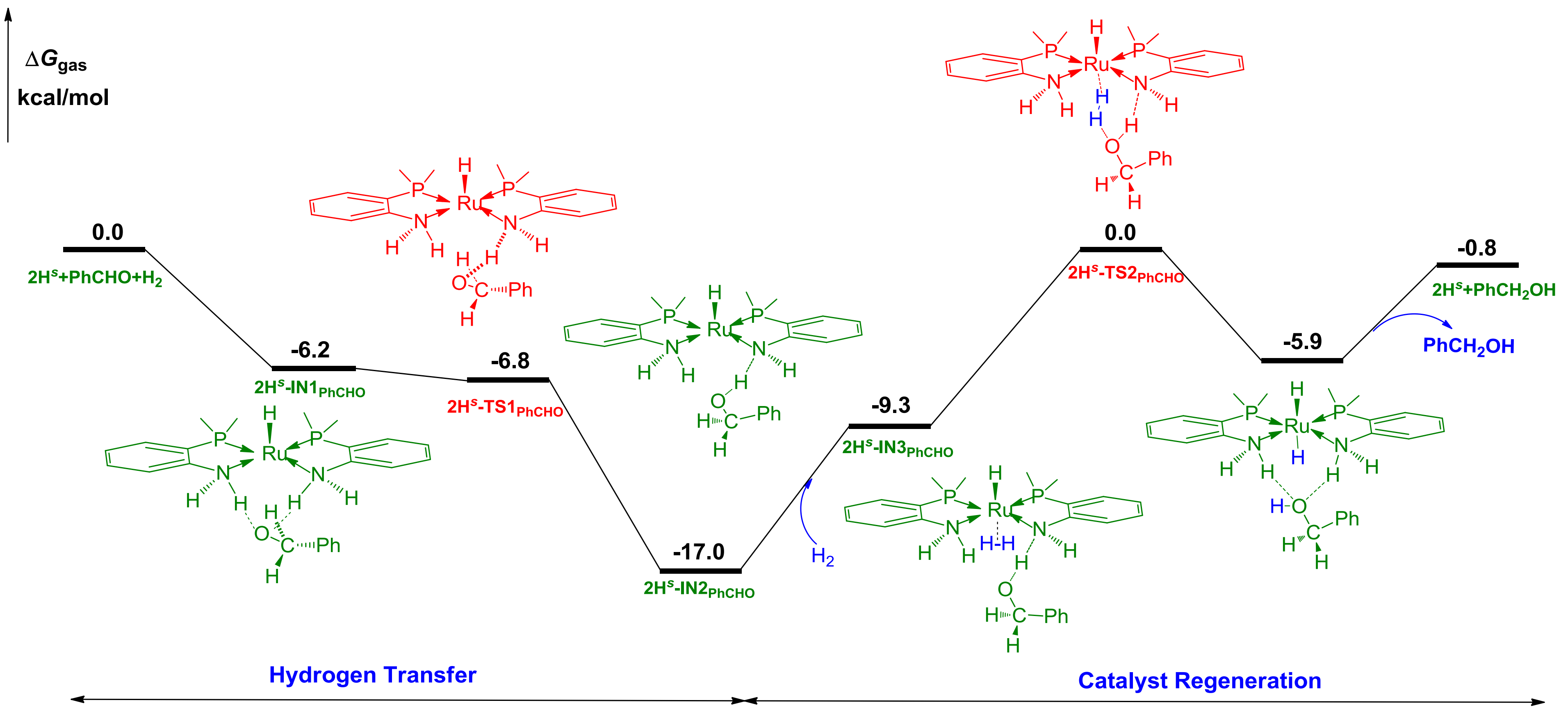

Figure S12. Free-energy profile for **2H**^{s} catalyzed hydrogenation of benzaldehyde to benzyl alcohol.

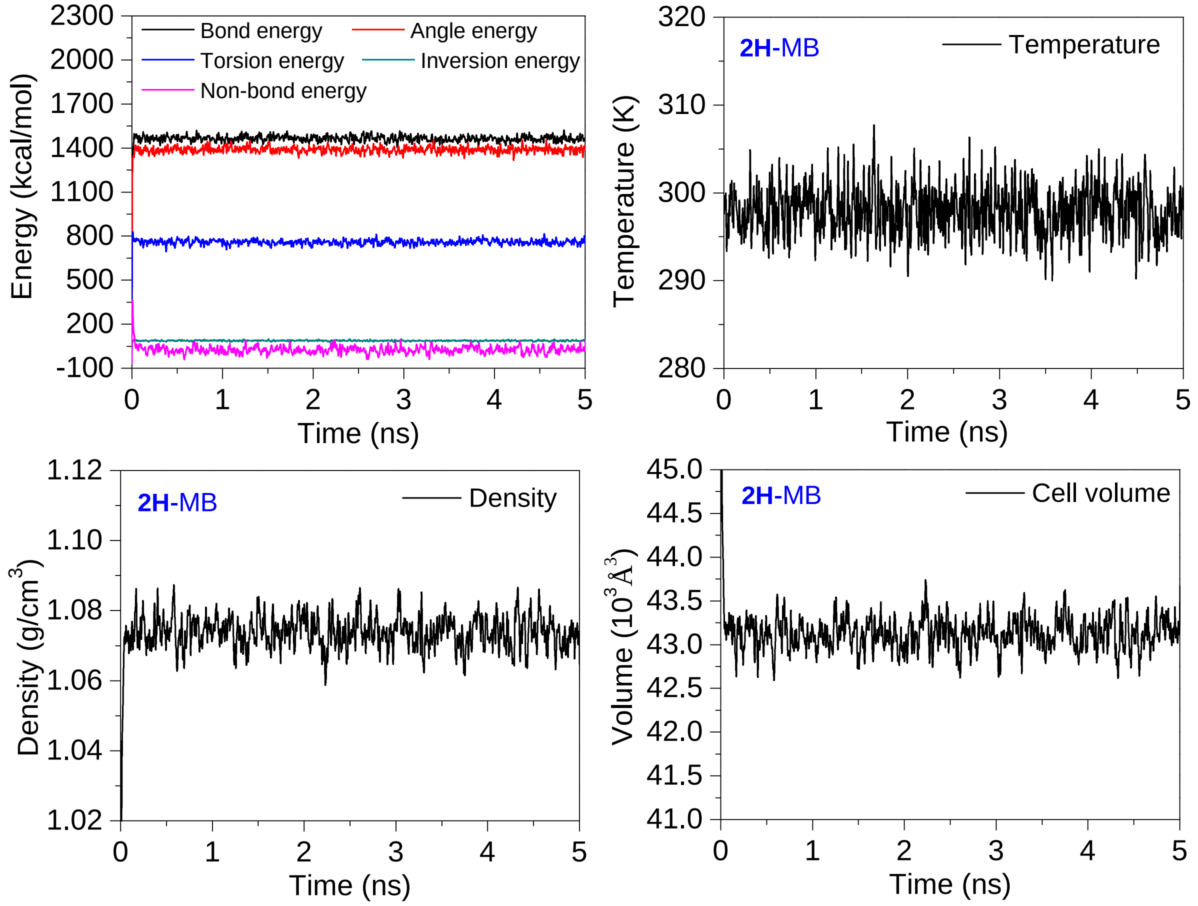

Figure S13. The time evolution of selected parameters such as energy, temperature, density and volume for the system **2H**-MB.

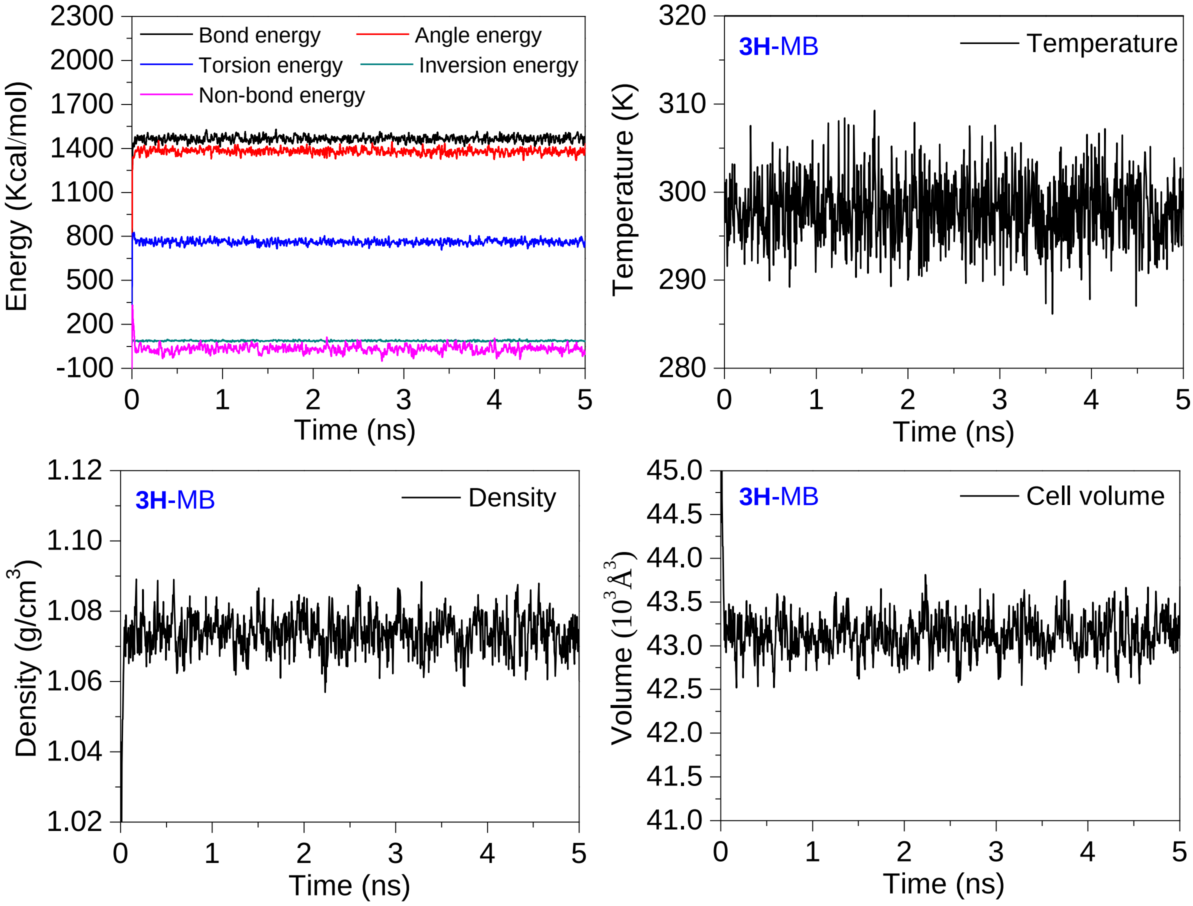

Figure S14. The time evolution of selected parameters such as energy, temperature, density and volume for the system **3H**-MB.

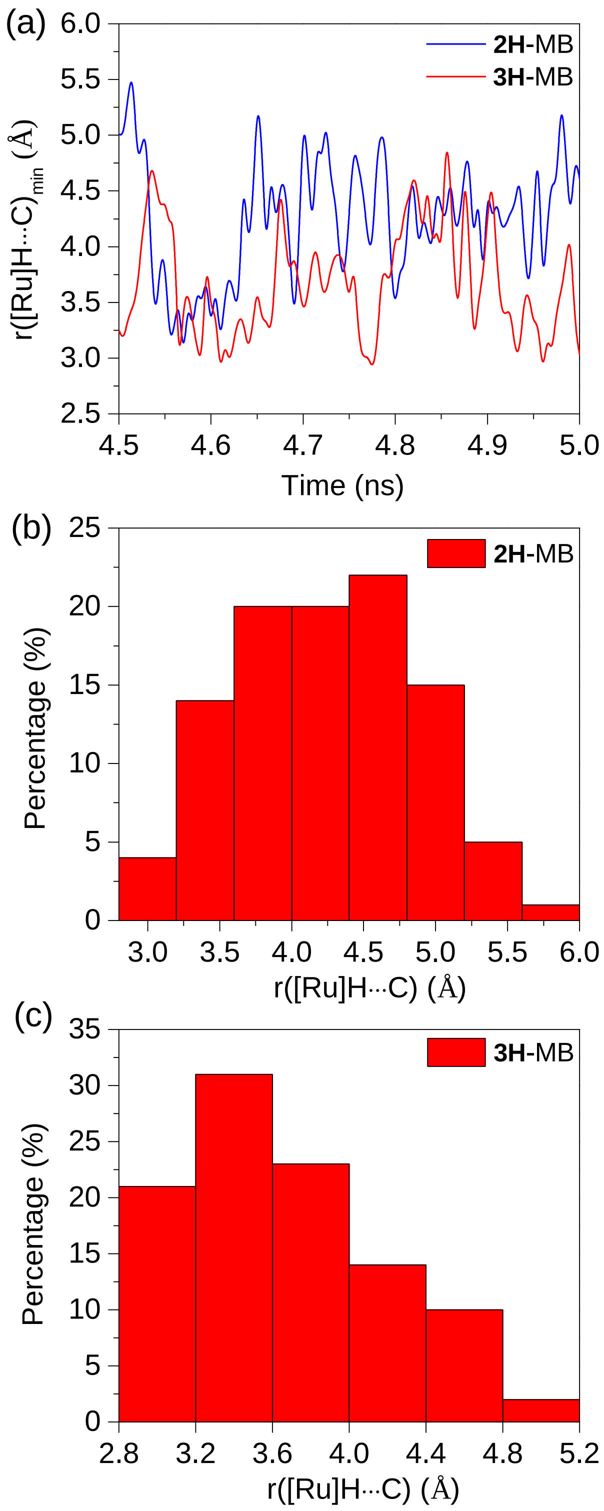

Figure S15. (a) The time evolution of the minimum distance between the ruthenium hydride and the carbonyl carbon of MB from MD simulations within 4.5~5.0 ns; **(b, c)** distribution of the r([Ru]H···C) distance for **2H**-MB and **3H**-MB.

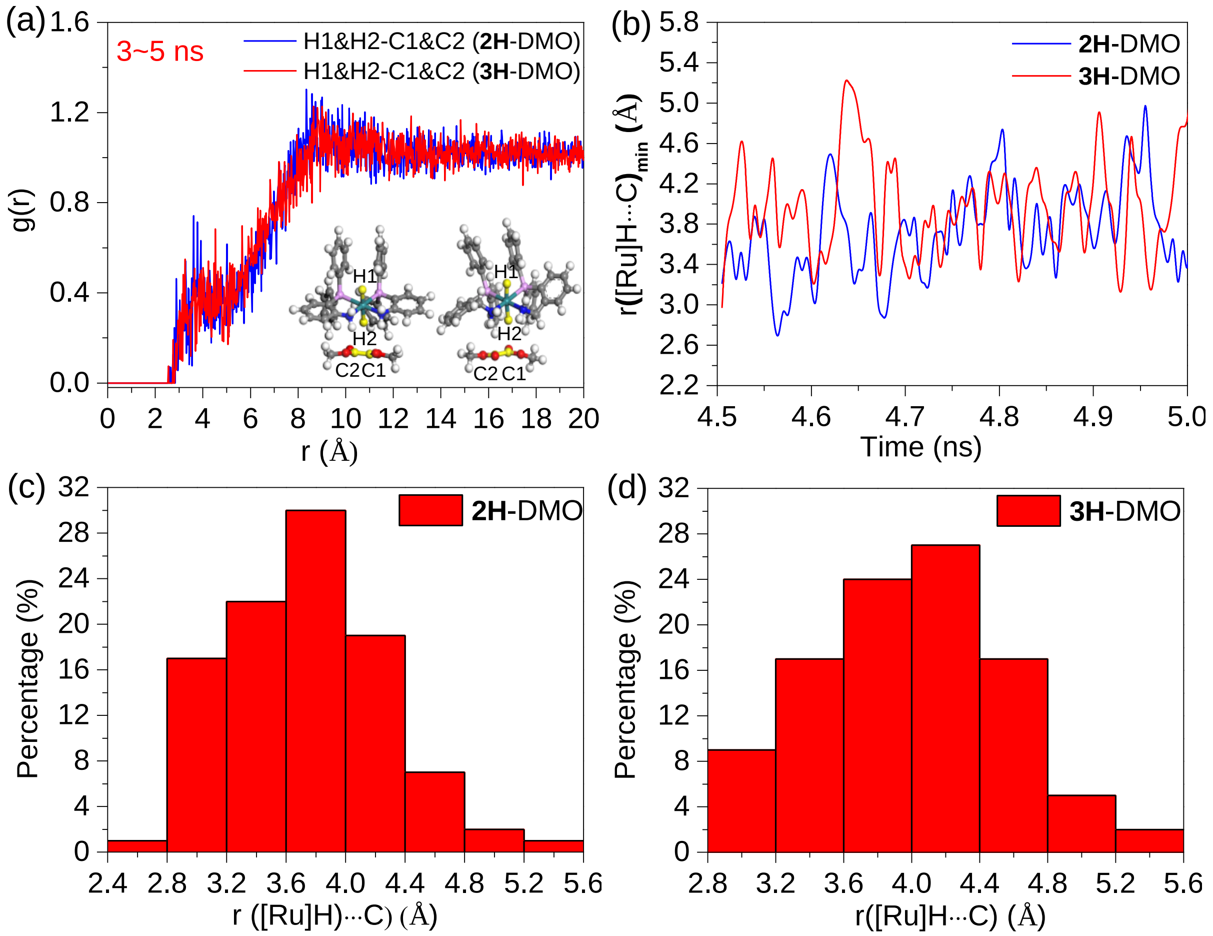

Figure S16. (a) The calculated partial pair correlation function g(r) for the distance between the ruthenium hydride and the carbonyl carbon of DMO from MD simulations within 3~5 ns; **(b)** the time evolution of the minimum distance between the ruthenium hydride and the carbonyl carbon of DMO within 4.5~5.0 ns; **(c, d)** distribution of the r([Ru]H ··C) distance for **2H**-DMO and **3H**-DMO.

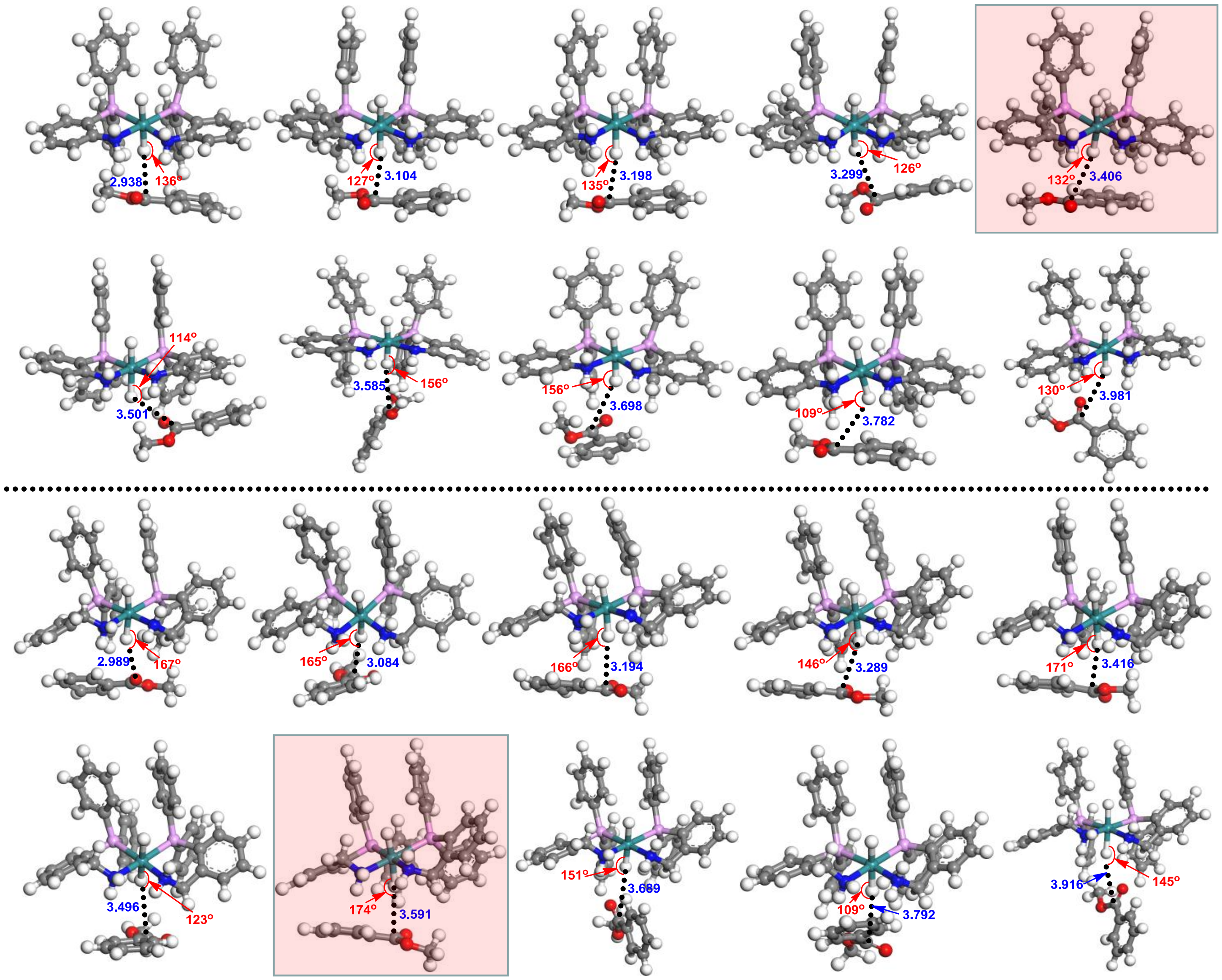

Figure S17. Snapshots of the interactions between the catalysts (**2H** & **3H**) and the substrate MB, where the distances between the ruthenium hydride and the carbonyl carbon are shown. In these interactive configurations, the Ru–H group is approximately perpendicular to the plane of the MB molecule, which is regarded as the most possible attack mode. We note that the angle (Ru–H ··C), the dihedral angle (Ru–H ··C=O), and the spatial position of the MB molecule change remarkably when the distance between the ruthenium hydride and the carbonyl carbon is larger than 3.4 Å and 3.6 Å for the system **2H**-MB and the system **3H**-MB, respectively.

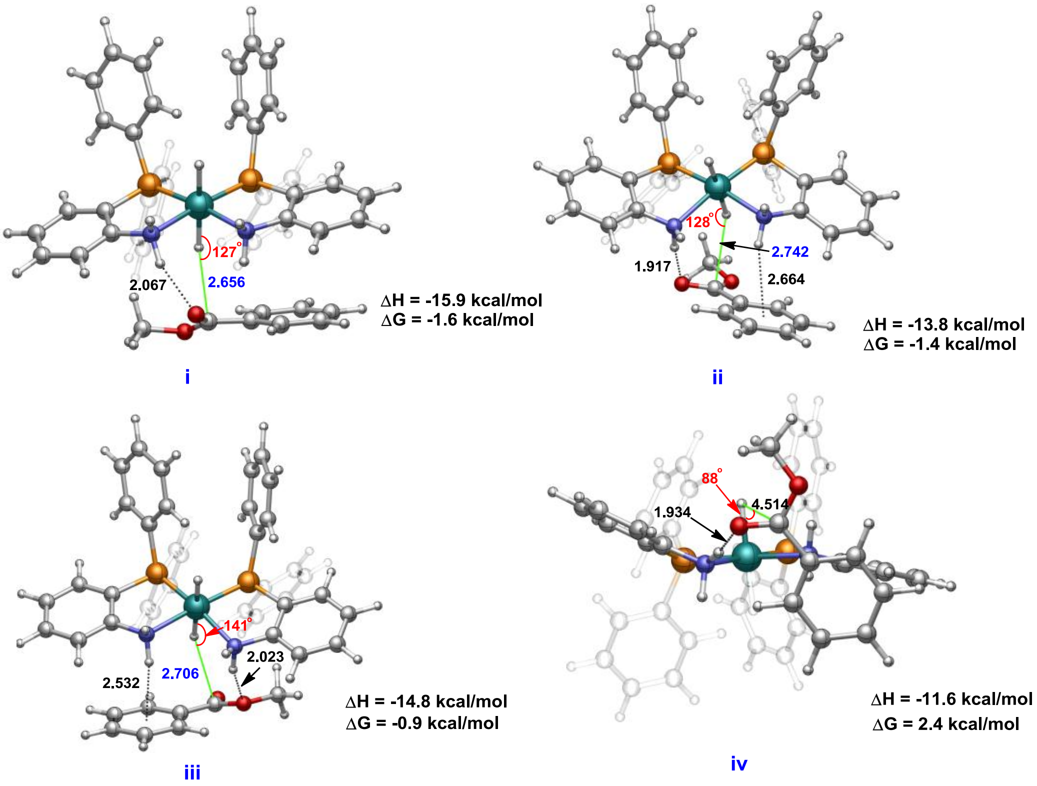

Figure S18. Optimized structures of the potential reactive conformers for the system **2H**-MB by DFT+D calculations. According to the DFT calculated results, the values of differences in thermal Enthalpies and Gibbs free energies are smaller than those for the system **3H**-MB (Fig. 8). To some extent, the data indicate that the optimized precursors of the system **2H**-MB are more stable than ones of the system **3H**-MB. Furthermore, catalysts **2H** and **3H** exhibit comparable activities in terms of calculated Gibbs free energy differences. Therefore, all the results above could not explain the catalytic performance observed in experiment and even violate the experimental phenomenon. Fortunately, the snapshots from MD simulations reasonably show that the binding model of near attack reactive configuration for **2H** hits the sole conformer **i**, while four different attack models (**i**–**iv**) are found arising from the interaction between the catalyst **3H** and MB (see Fig. 8 and Fig. S17). Apparently, the results suggest that the steric interactions manipulated by the CH_2 linkage of *o*-$PPh_2C_6H_4CH_2NH_2$ dominate the accessibility of the reactive conformer. Conceivably, we also note that the Ru–H ··C angles of the conformers **ii** and **iii** are larger than those of the conformers **i** and **iv** for **3H**-MB (Fig. 8), which determines whether the hydrogen transfer process is in favorable orientation. Meanwhile, the angle (Ru–H ··C) of 127 degree in the sole conformer of **2H**-MB is generally smaller than those in **3H**-MB, indicating the corresponding hydrogen transfer is less favorable, compared to the **3H**-MB system. Such directional features in the initial reactive conformers account for the observed differences in the catalytic performance for the Ru-based catalysts.

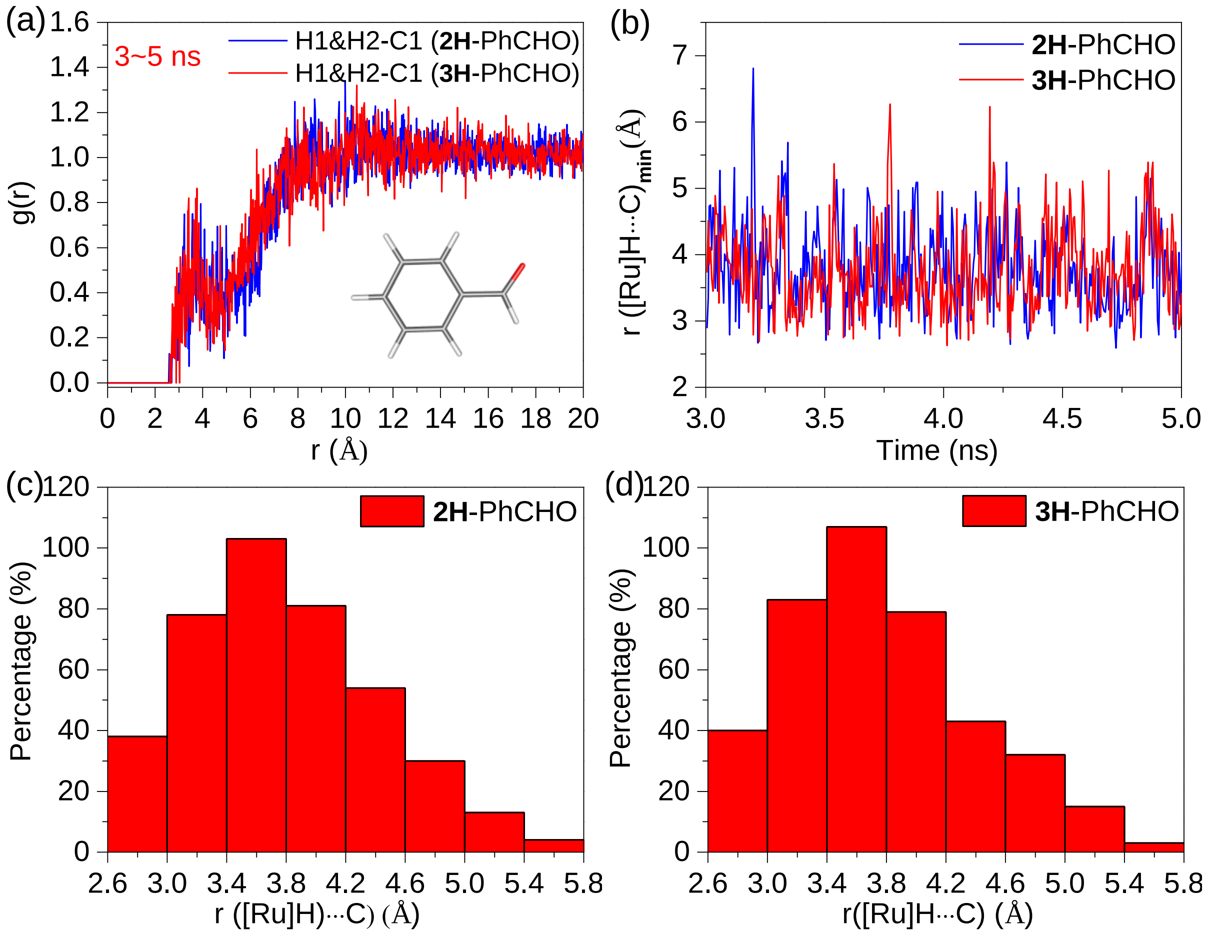

Figure S19. (a) The calculated partial pair correlation function g(r) for the distance between the ruthenium hydride and the carbonyl carbon of benzaldehyde from MD simulations within 3~5 ns; **(b)** the time evolution of the minimum distance between the ruthenium hydride and the carbonyl carbon of benzaldehyde from 3.0 to 5.0 ns; **(c, d)** distribution of the r([Ru]H ··C) distance for **2H**-PhCHO and **3H**-PhCHO. Apparently, there are similar distribution features of reactive conformers for the systems **2H**-PhCHO and **3H**-PhCHO during MD simulations. In comparison with the results in Fig. 7c, 7d and Fig. S15, the existence of the methoxyl group may make the catalyst **2H** less active toward the hydrogenation of MB.

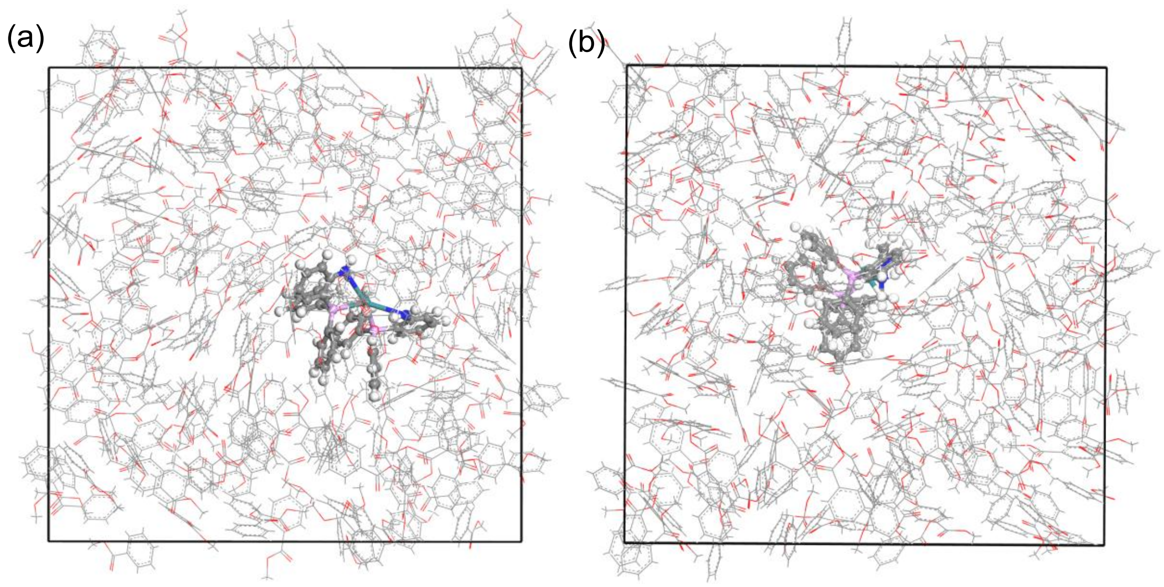

Figure S20. The condensed-phase model of the reaction system containing substrates, catalysts, and solvents for MD simulations with the periodic boundary condition.

V. NMR Spectra

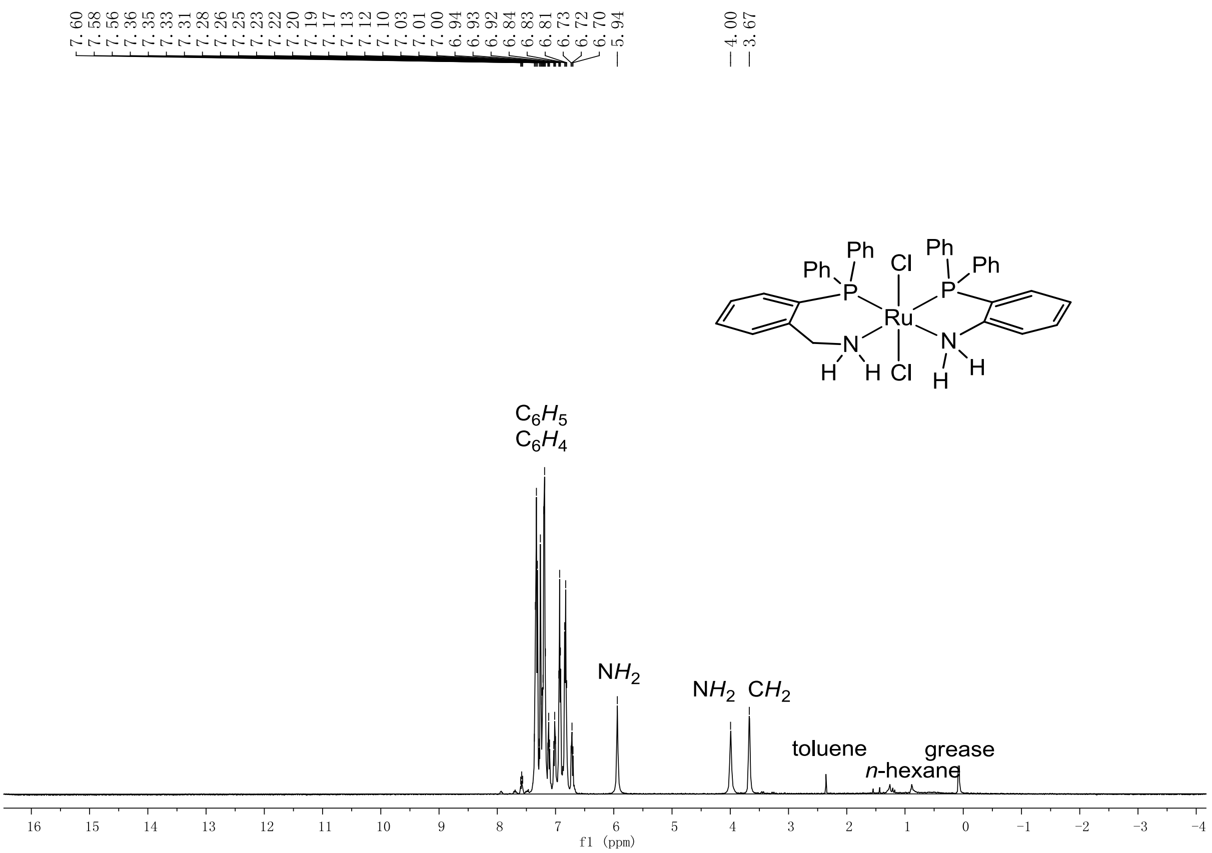

Figure S21. ^{1}H NMR spectrum of complex **4** in $CDCl_3$.

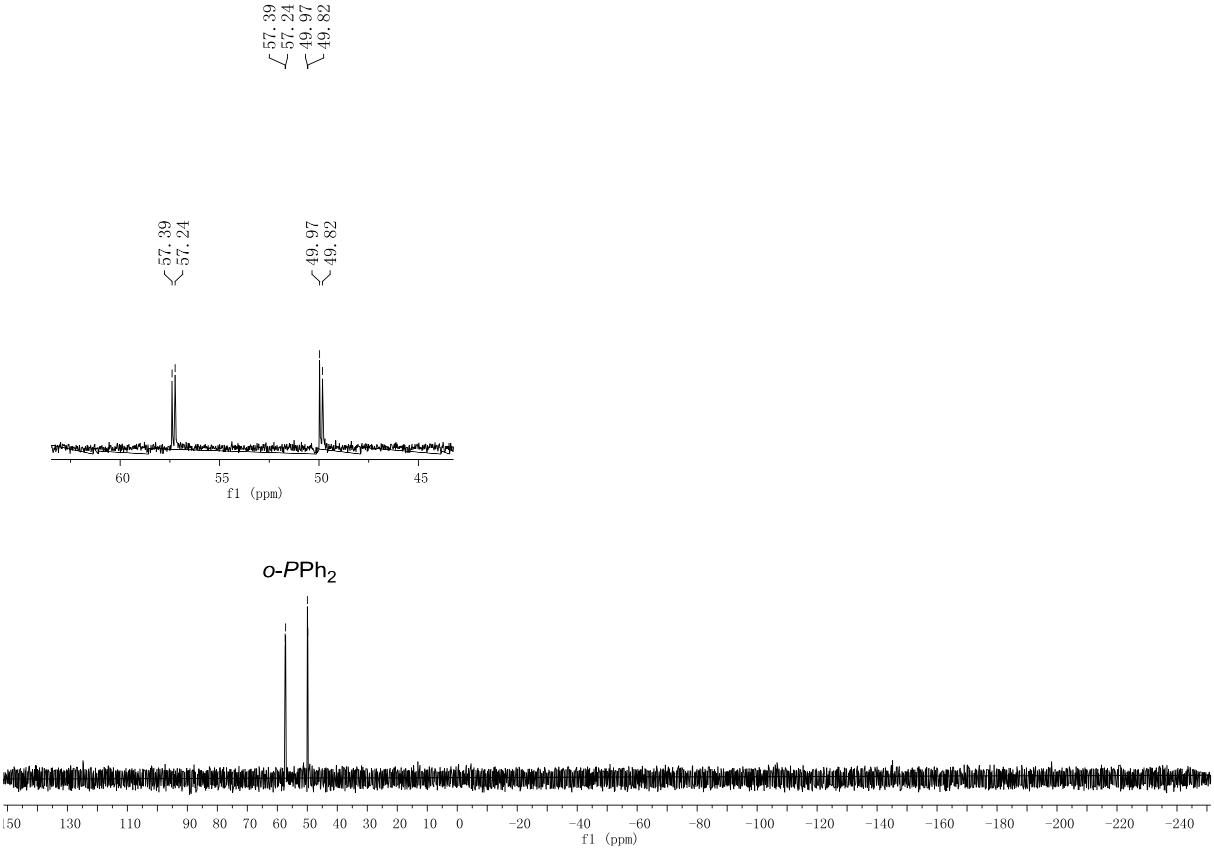

Figure S22. $^{31}P\{^{1}H\}$ NMR spectrum of complex **4** in $CDCl_3$.

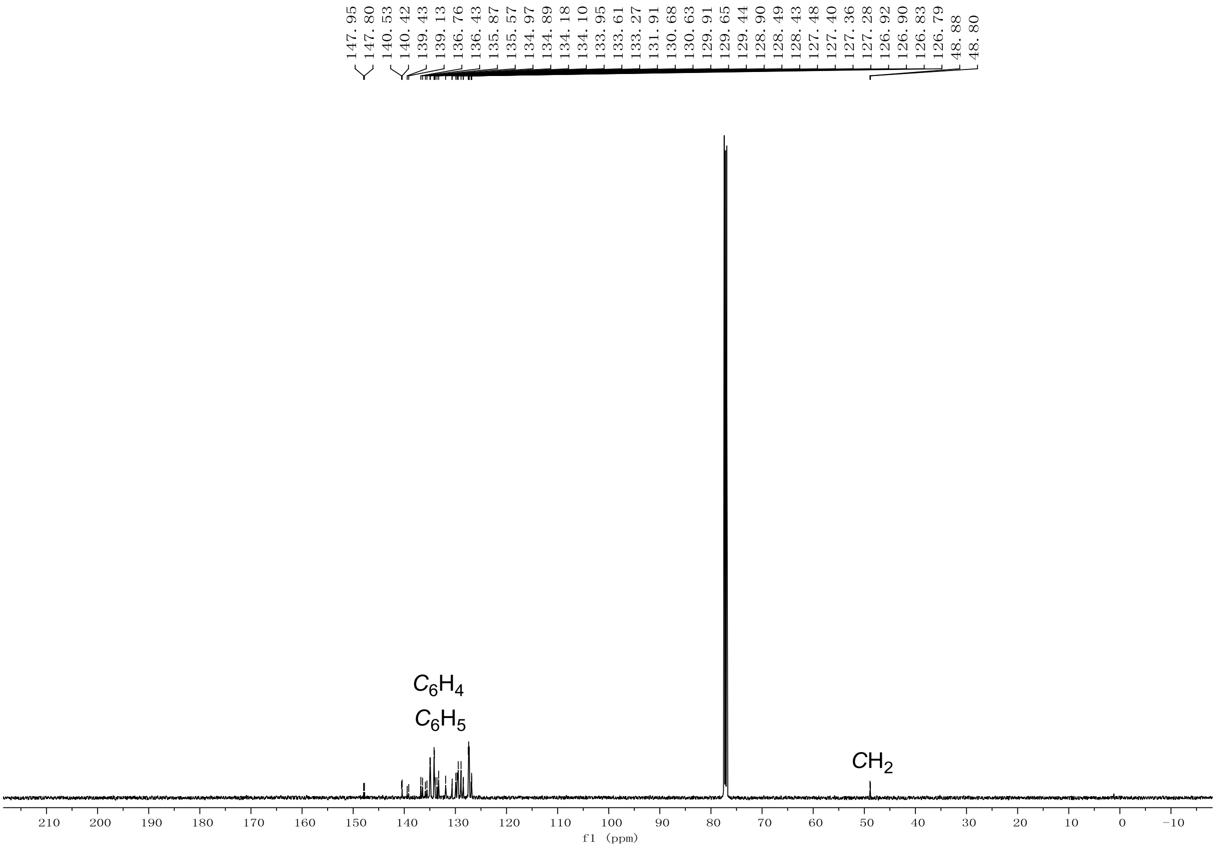

Figure S23. ^{3}C{^{1}H} NMR spectrum of complex **4** in $CDCl_3$.

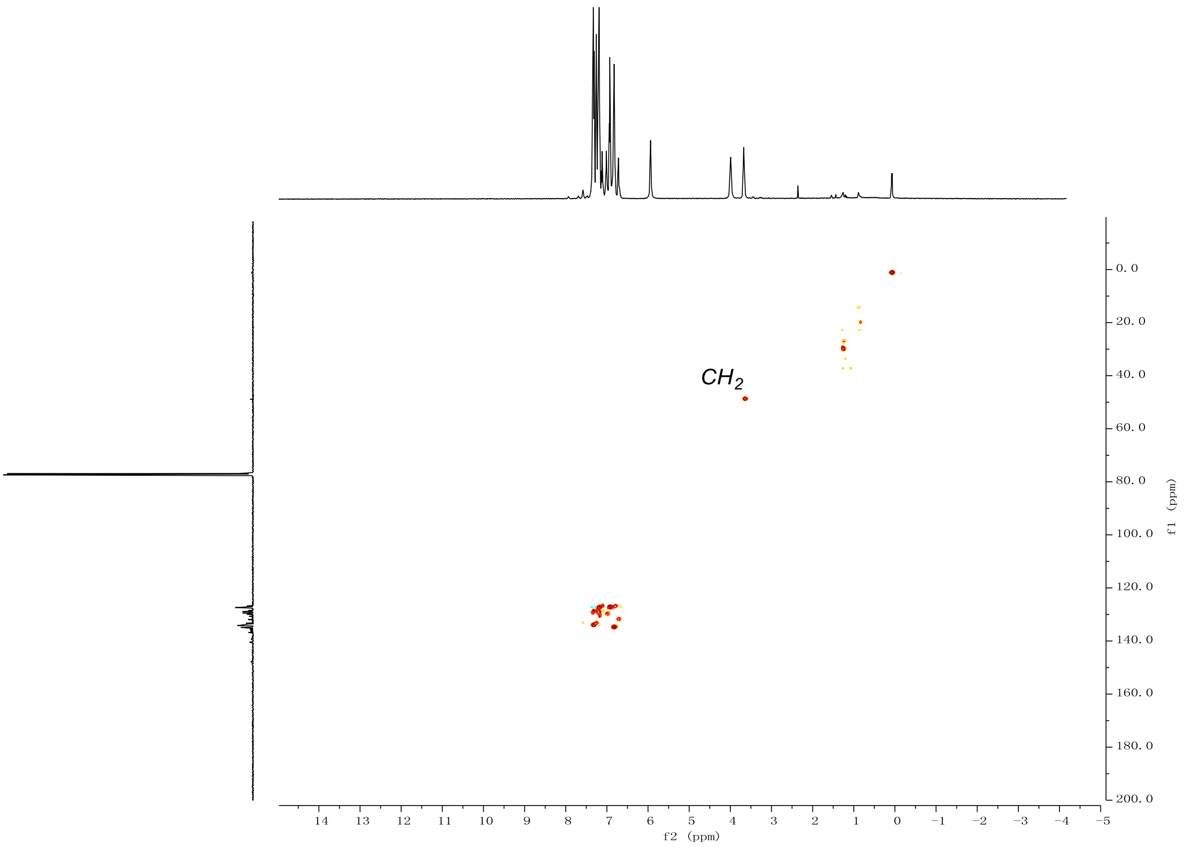

Figure S24. ^{1}H-^{13}C HSQC NMR spectrum of complex **4** in $CDCl_3$.

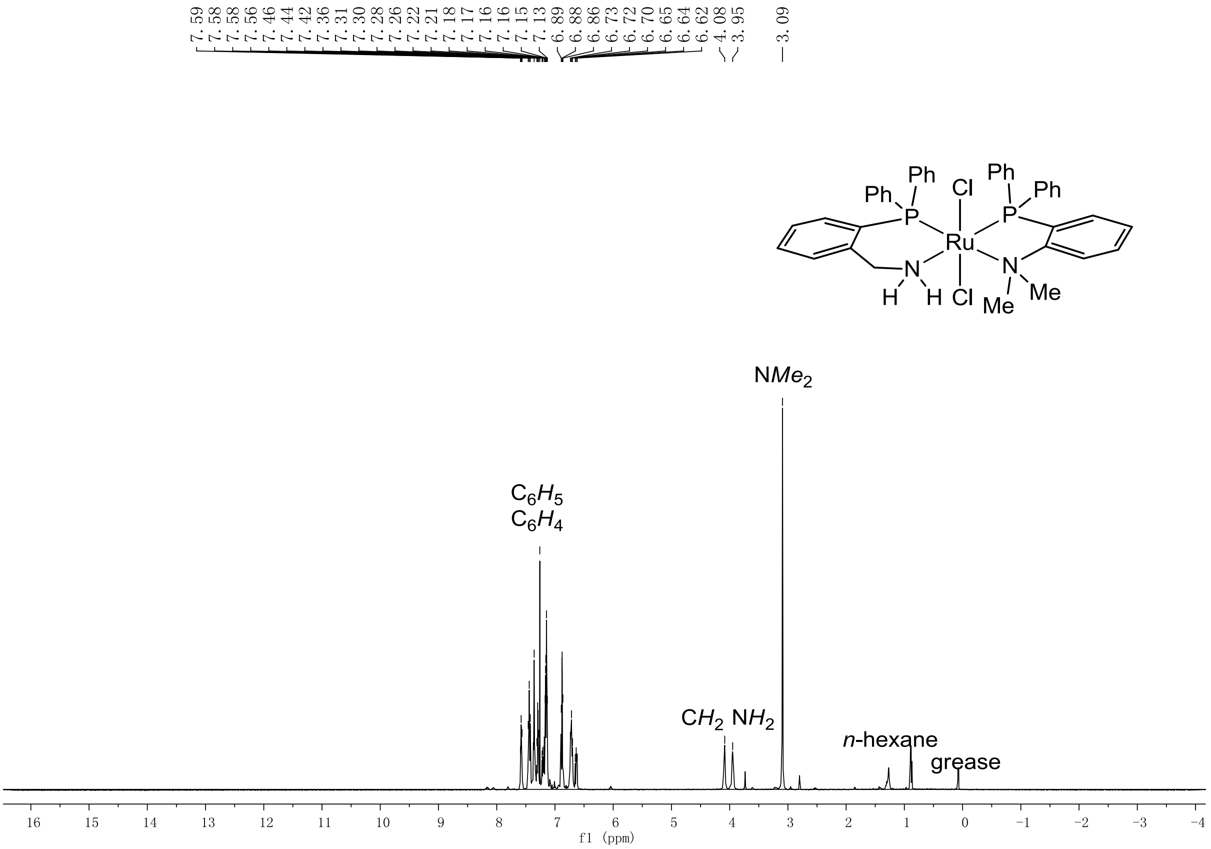

Figure S25. ^{1}H NMR spectrum of complex **5** in $CDCl_3$.

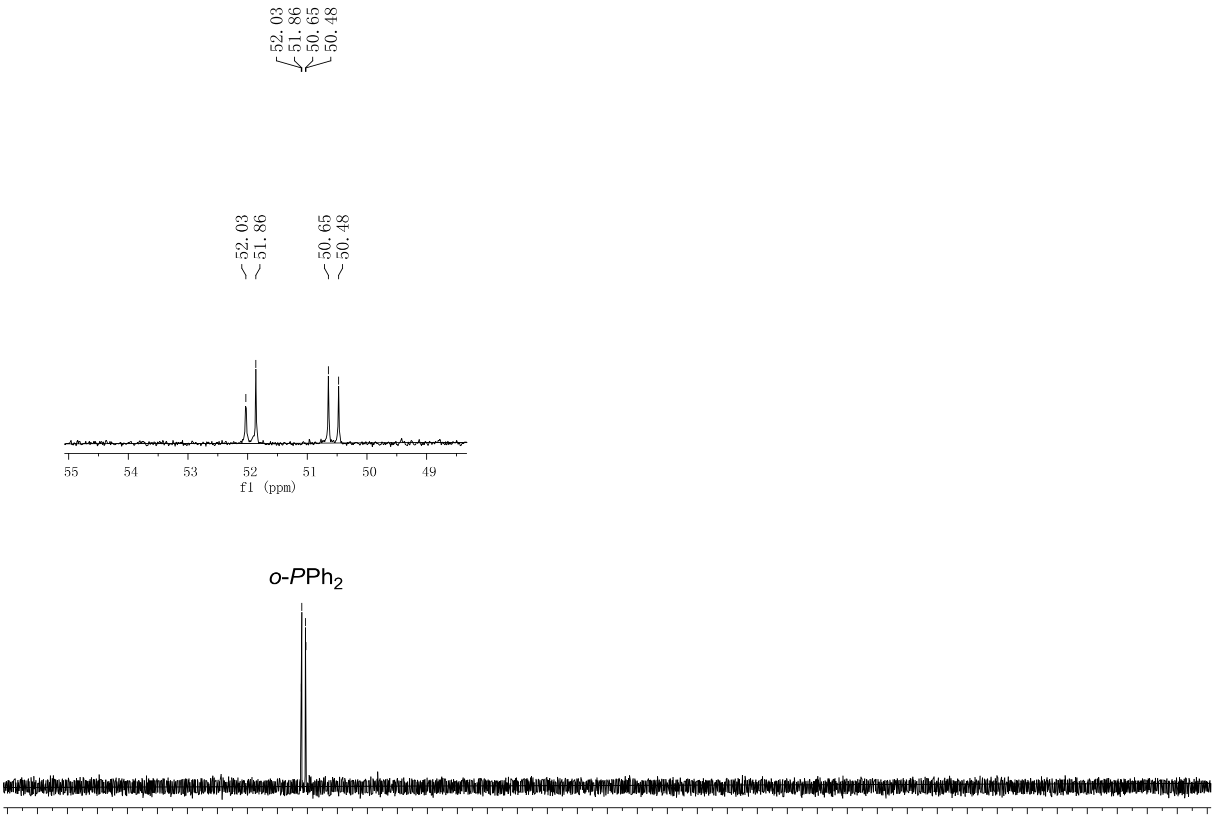

Figure S26. $^{31}P\{^{1}H\}$ NMR spectrum of complex **5** in $CDCl_3$.

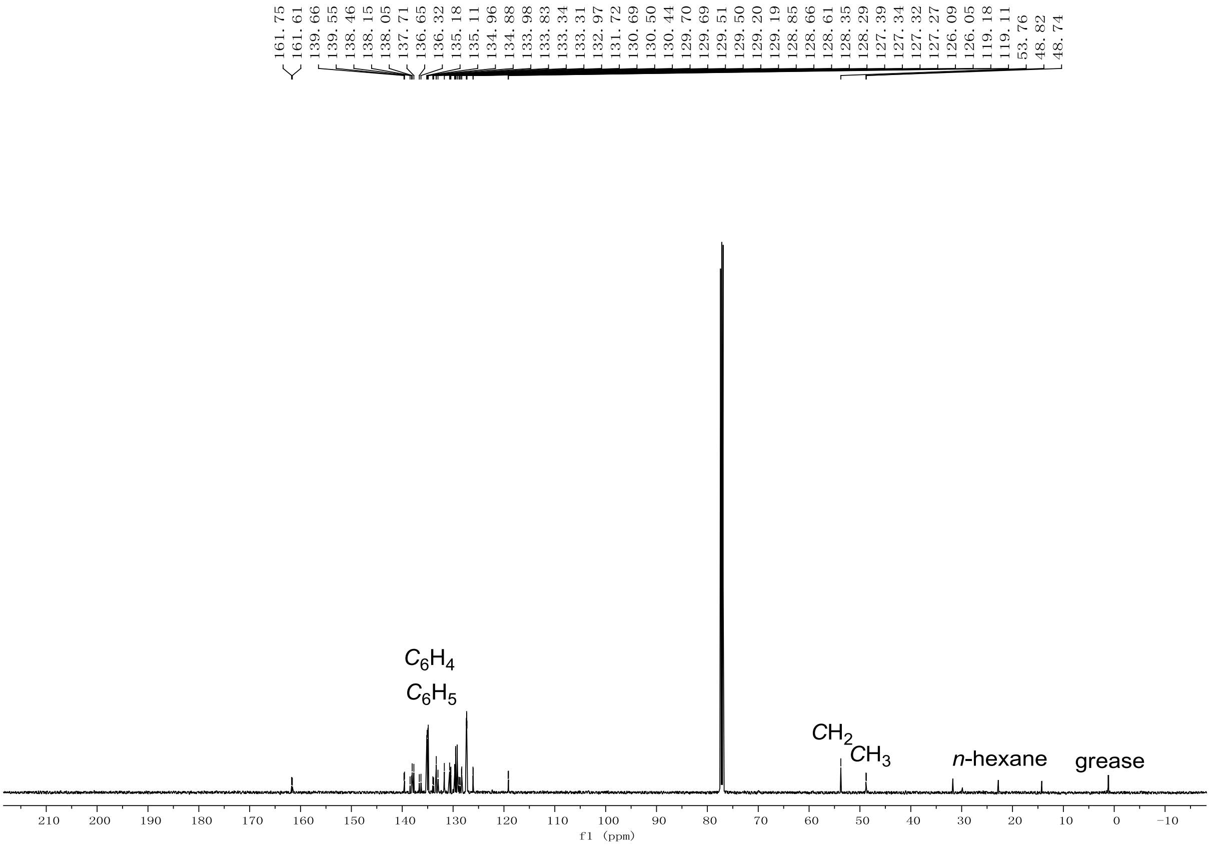

Figure S27. $^{13}C\{^{1}H\}$ NMR spectrum of complex **5** in $CDCl_3$.

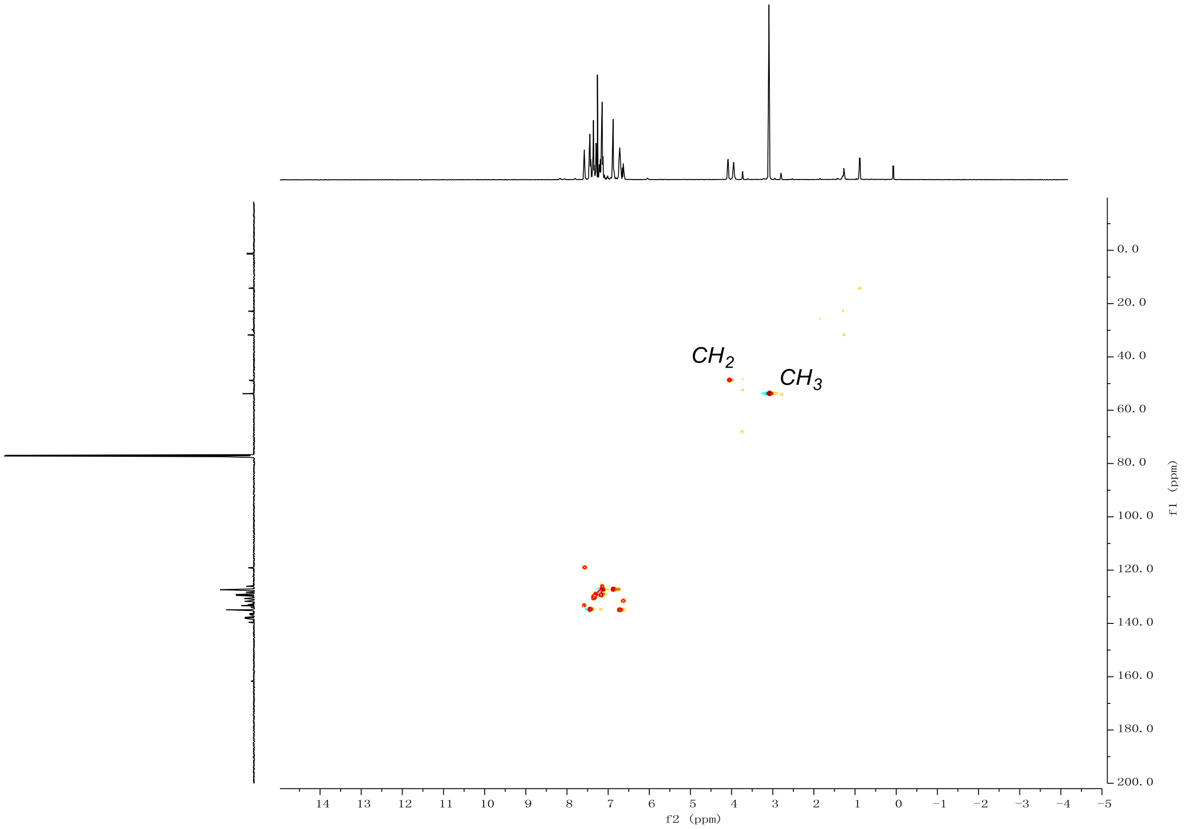

Figure S28. ^{1}H-^{13}C HSQC NMR spectrum of complex **5** in $CDCl_3$.

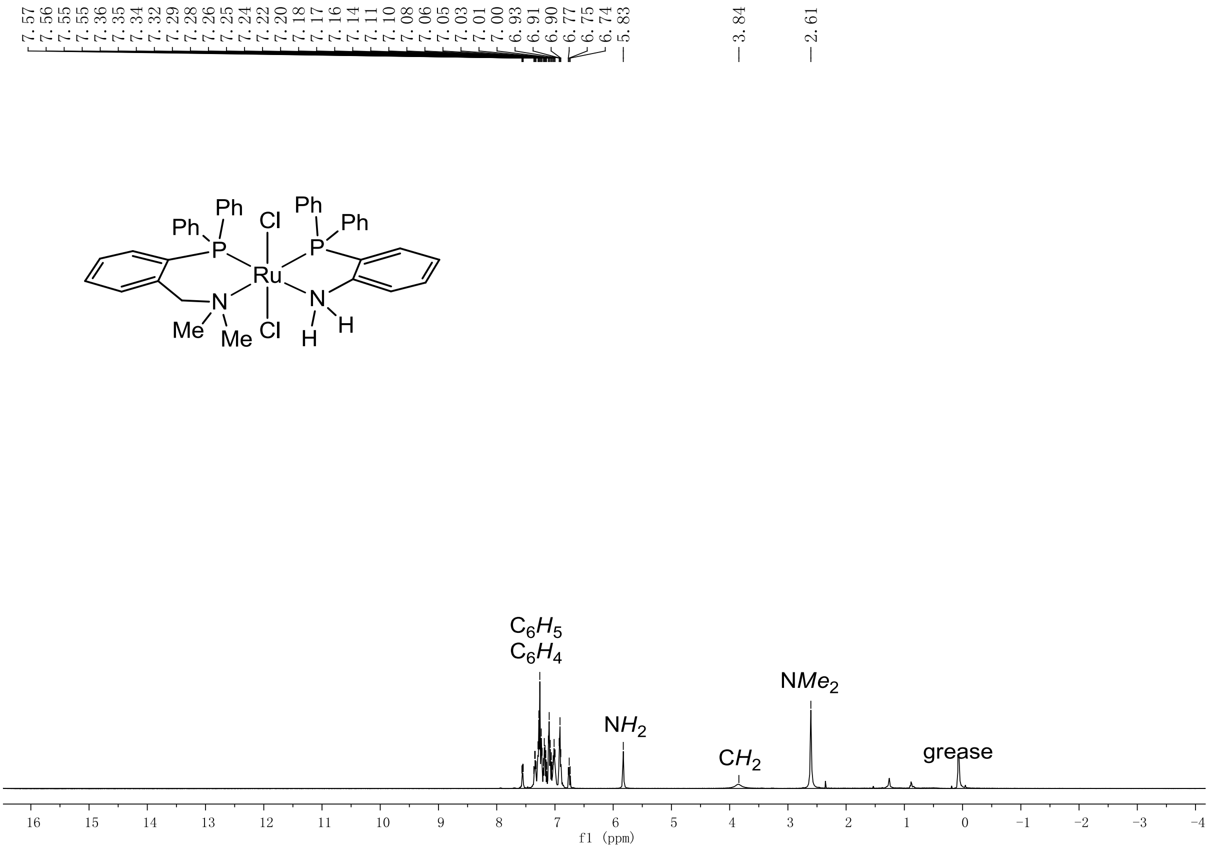

Figure S29. ^{1}H NMR spectrum of complex **6** in $CDCl_3$.

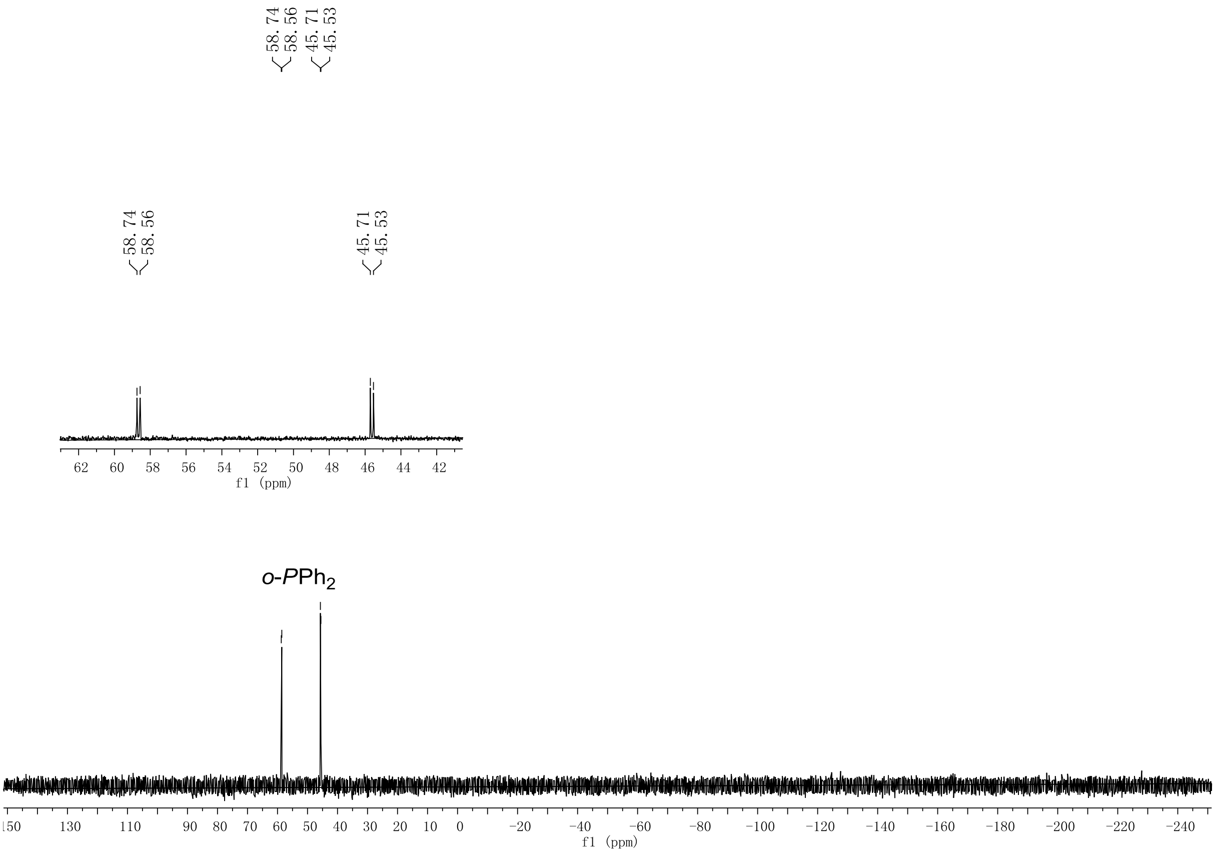

Figure S30. $^{31}P\{^{1}H\}$ NMR spectrum of complex **6** in $CDCl_3$.

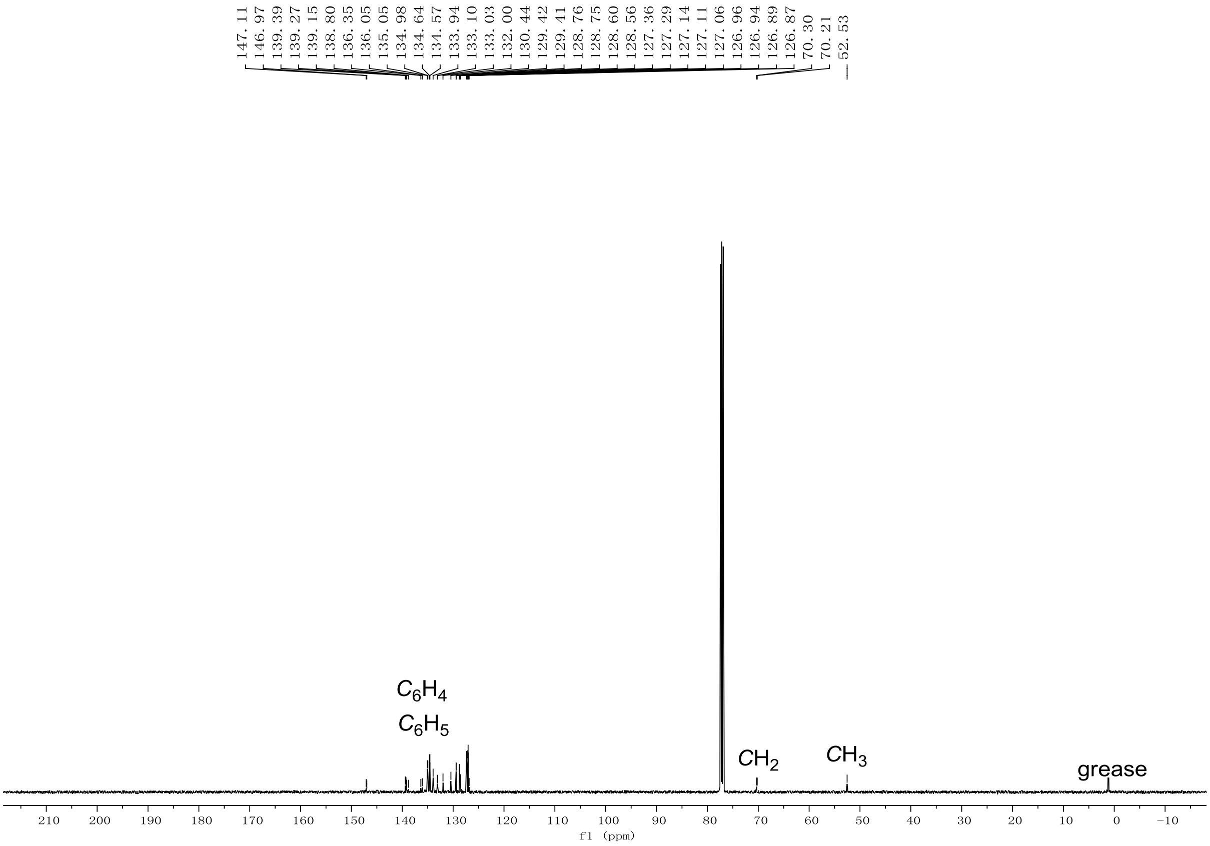

Figure S31. $^{13}C\{^{1}H\}$ NMR spectrum of complex **6** in $CDCl_3$.

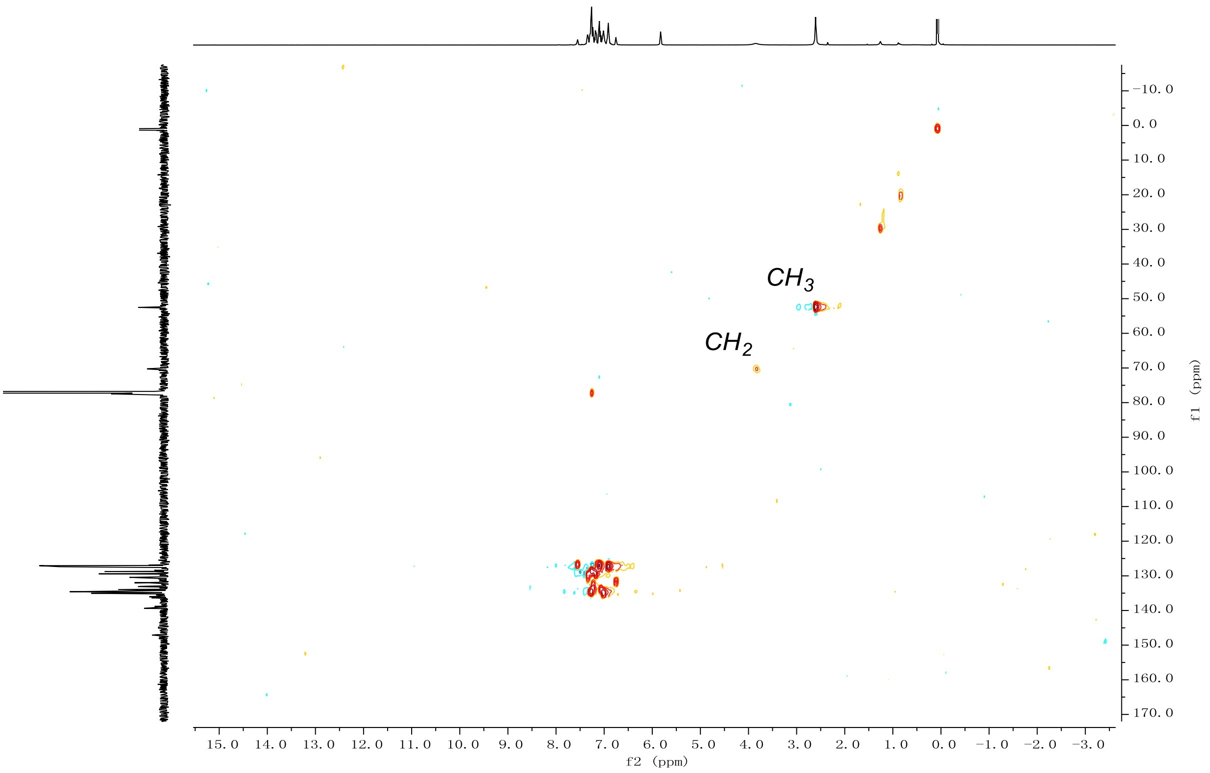

Figure S32. ^{1}H-^{13}C HSQC NMR spectrum of complex **6** in $CDCl_3$.

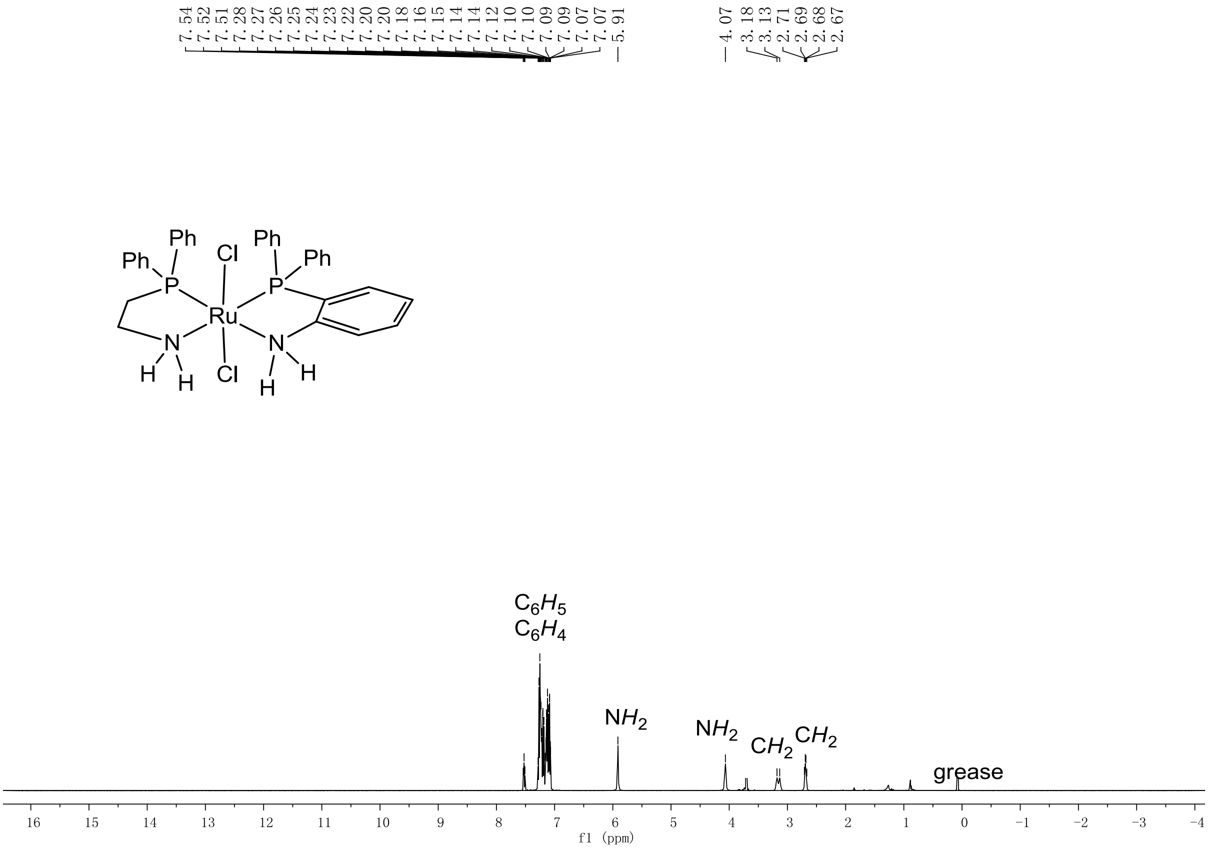

Figure S33. ^{1}H NMR spectrum of complex **9** in $CDCl_3$.

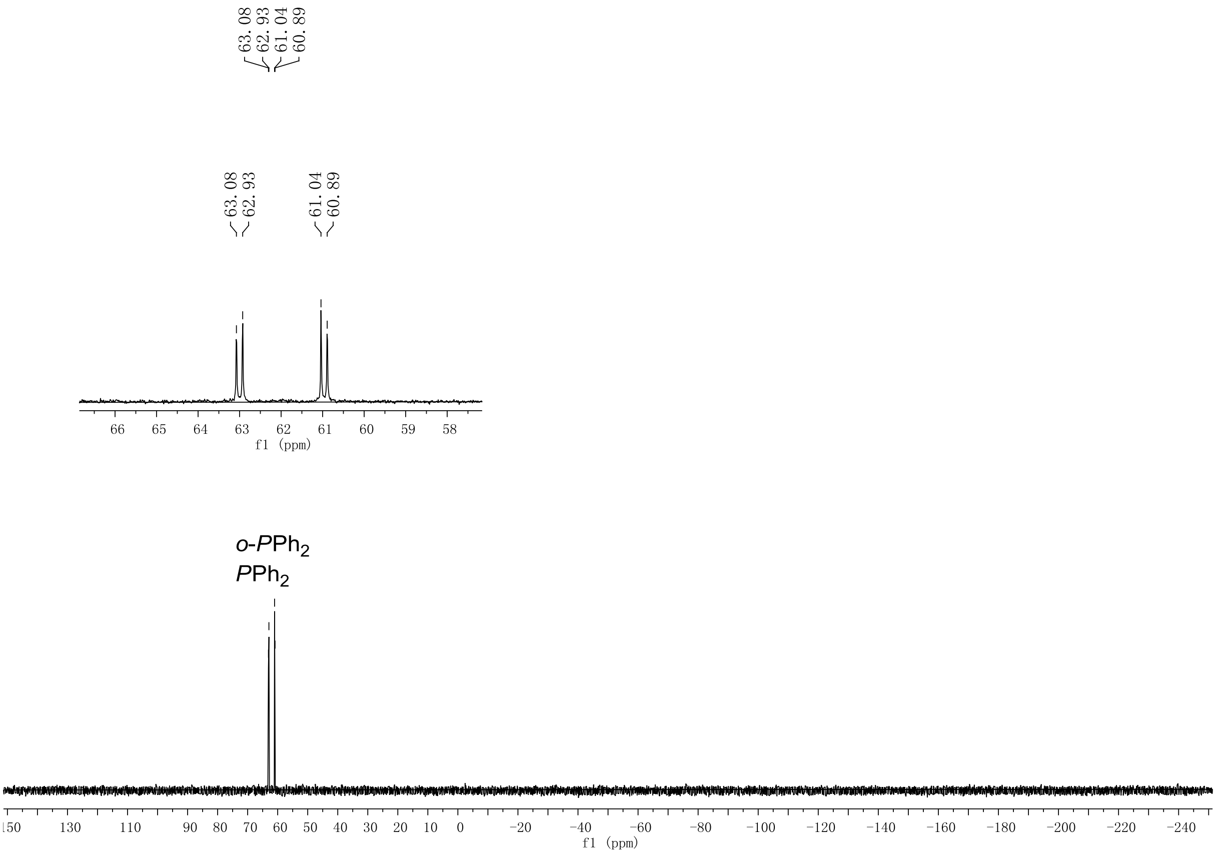

Figure S34. ^{31}P{^{1}H} NMR spectrum of complex **9** in $CDCl_3$.

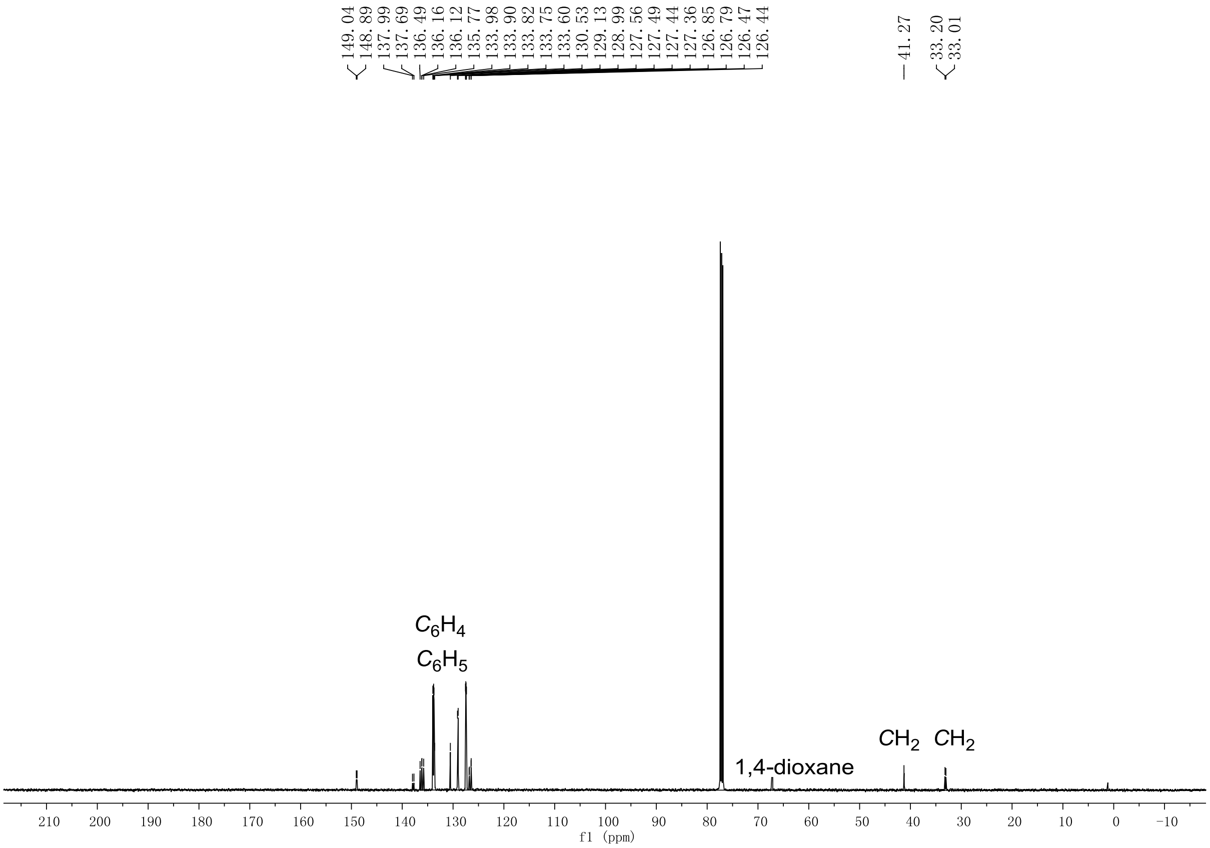

Figure S35. $^{13}C\{^{1}H\}$ NMR spectrum of complex **9** in $CDCl_3$.

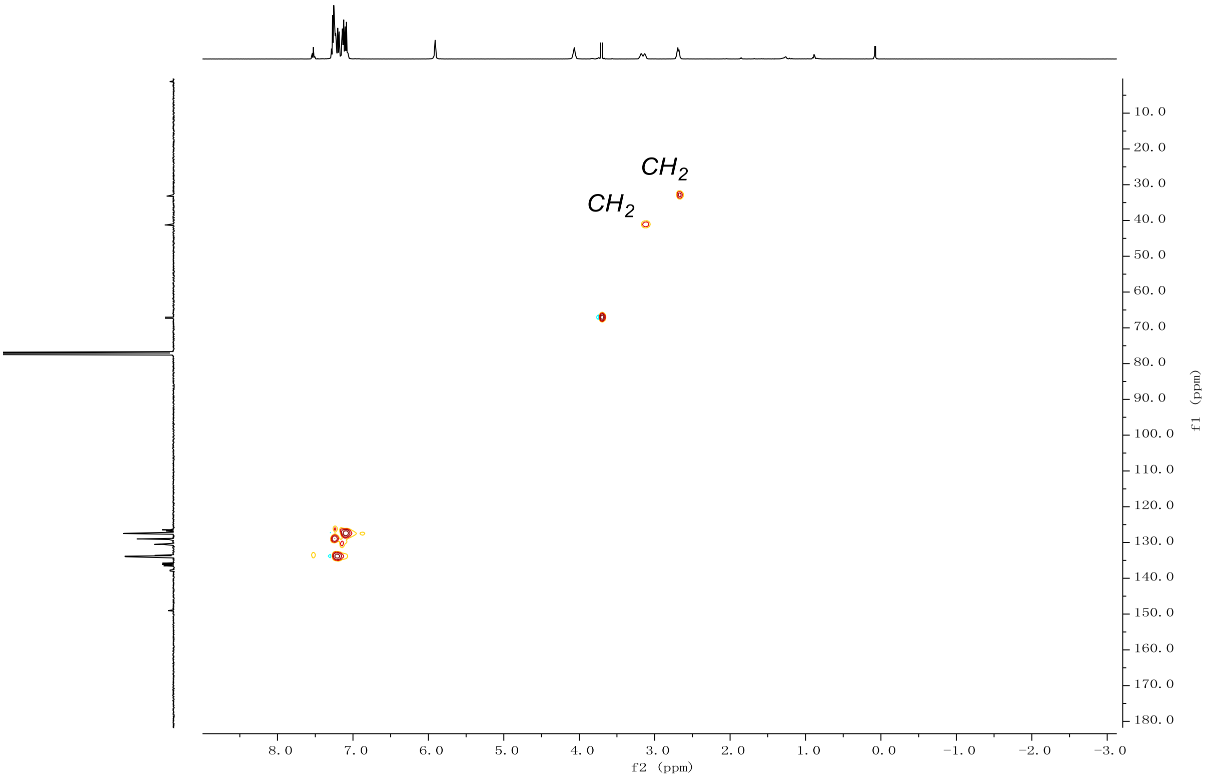

Figure S36. ^{1}H-^{13}C HSQC NMR spectrum of complex **9** in $CDCl_3$.

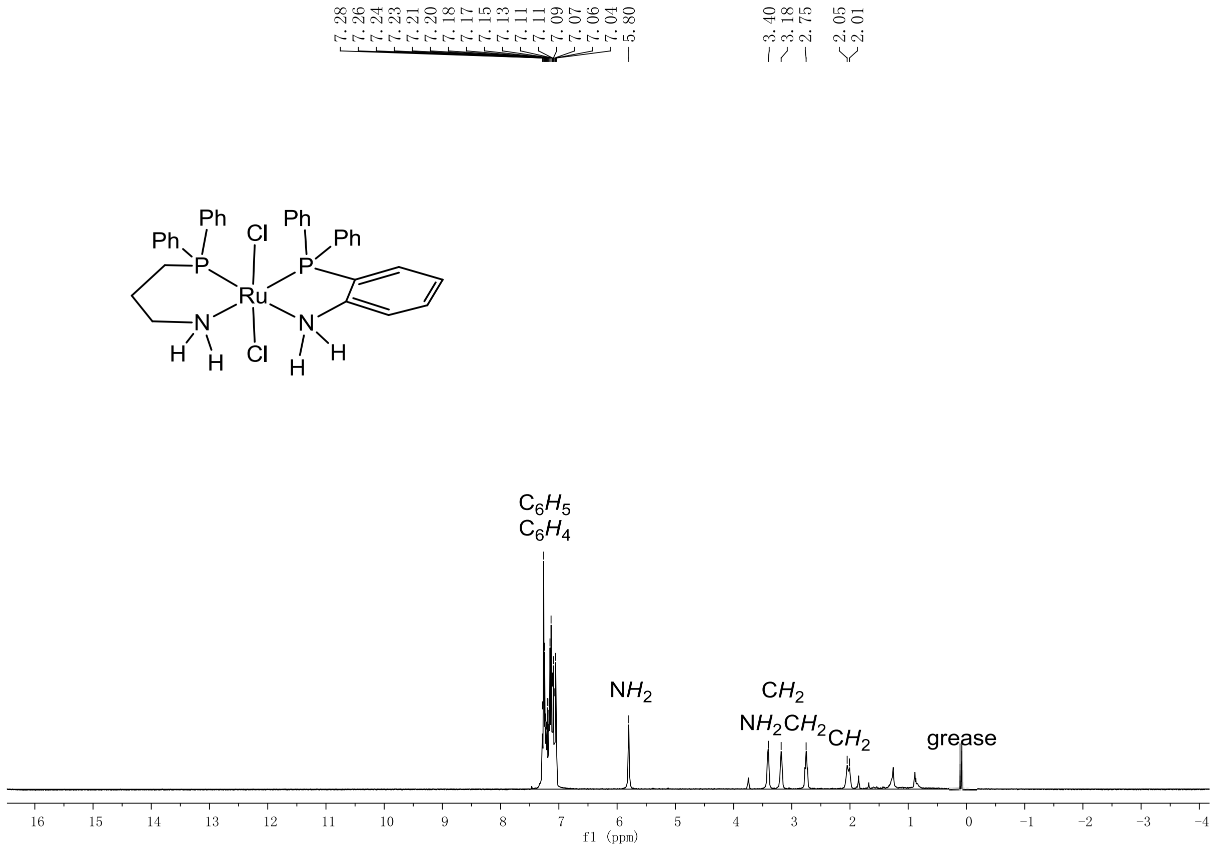

Figure S37. ^{1}H NMR spectrum of complex **10** in $CDCl_3$.

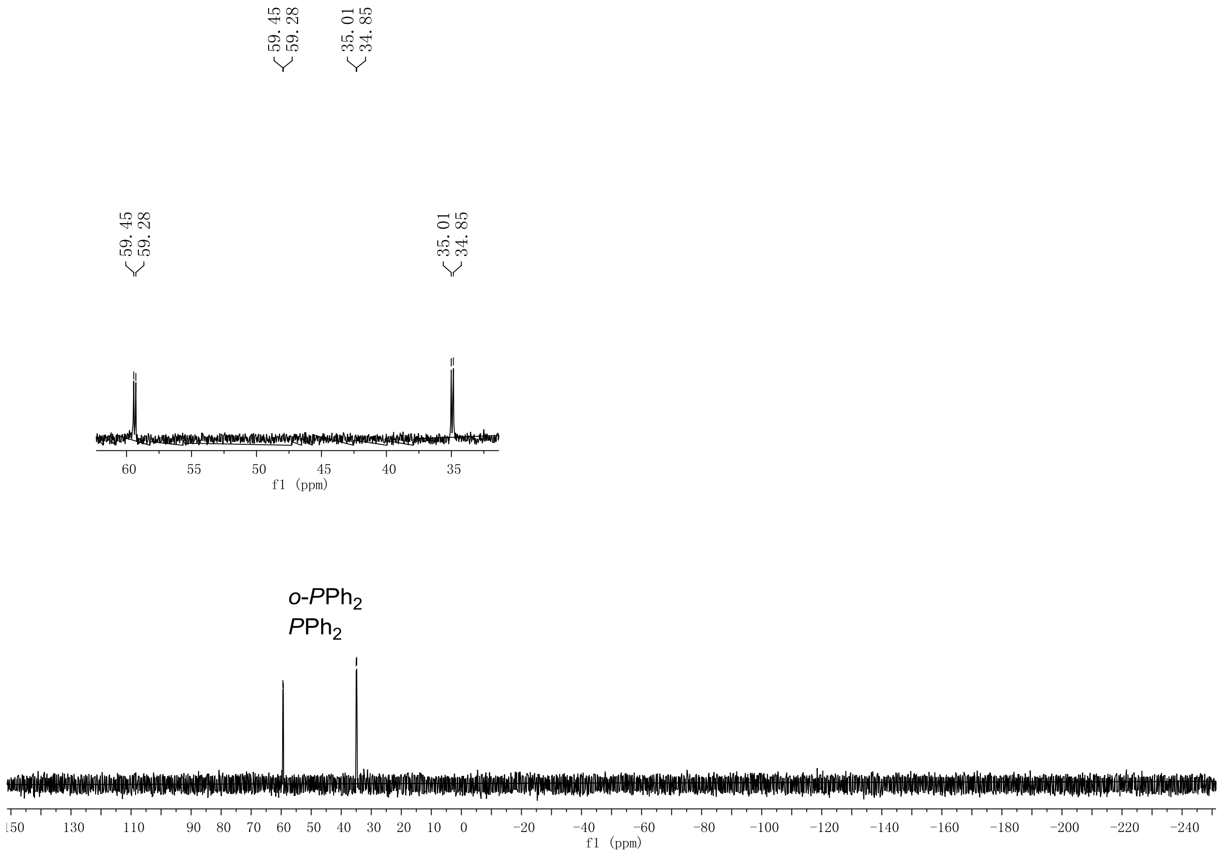

Figure S38. $^{31}P\{^{1}H\}$ NMR spectrum of complex **10** in $CDCl_3$.

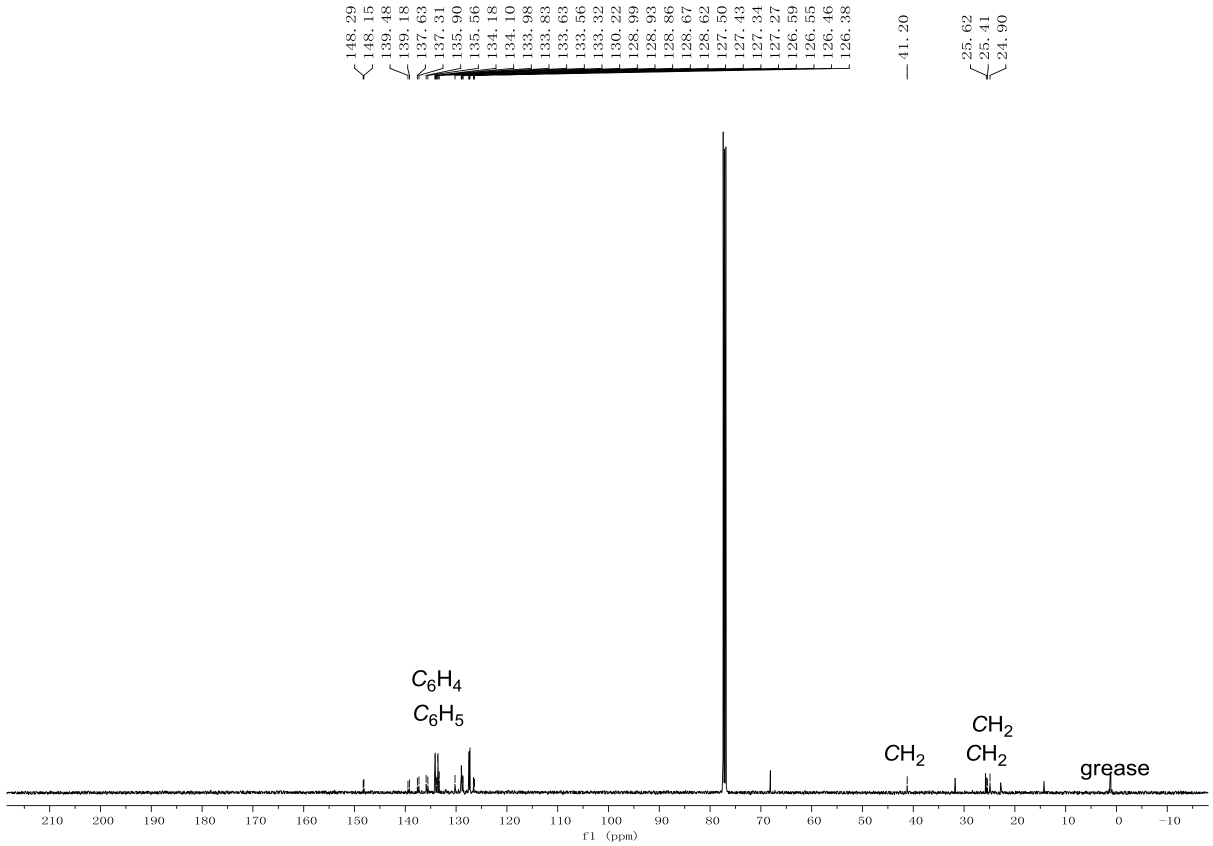

Figure S39. $^{13}C\{^{1}H\}$ NMR spectrum of complex **10** in $CDCl_3$.

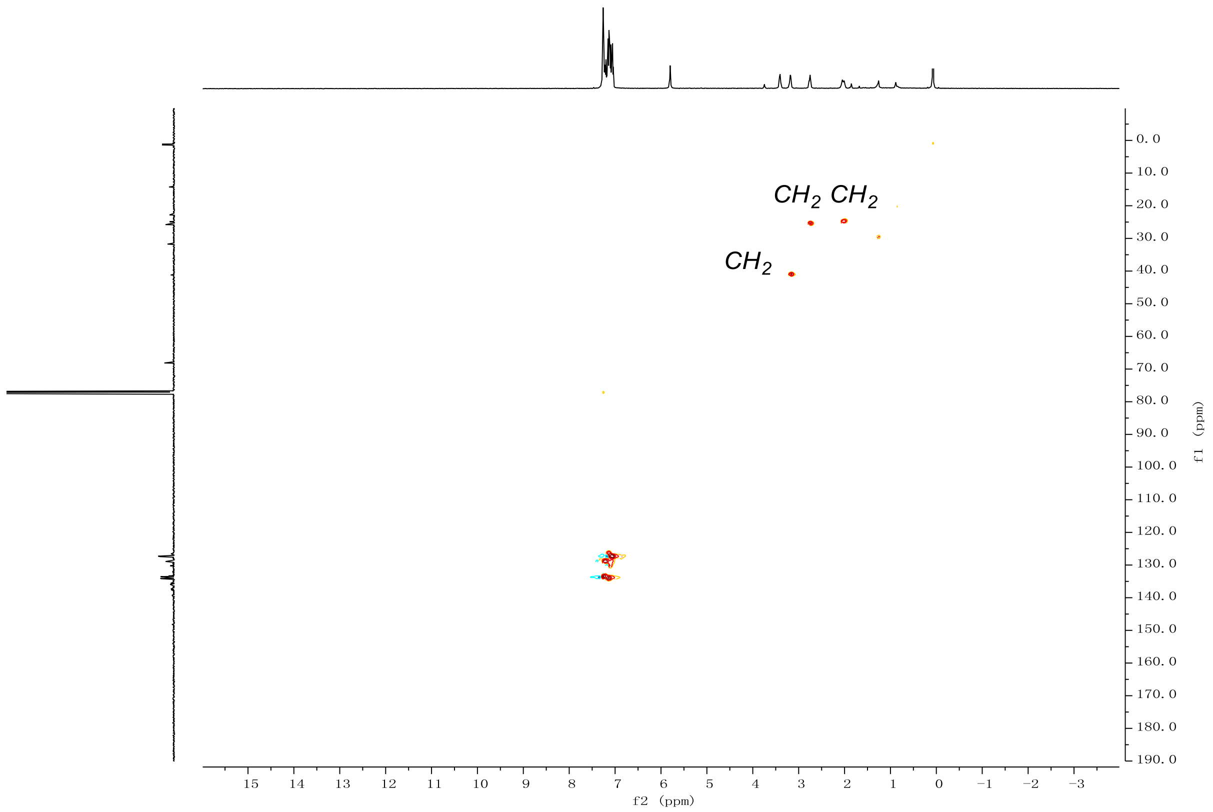

Figure S40. ^{1}H-^{13}C HSQC NMR spectrum of complex **10** in $CDCl_3$.

VI. Cartesian coordinates for the optimized geometries of the reactants, transition states and intermediates

DMO

C	-0.70303500	0.31970200	0.00006200
O	-0.90978200	1.51162600	0.00012800
O	-1.63871000	-0.63268700	-0.00004900
C	0.70303400	-0.31970200	0.00005000
O	0.90978000	-1.51162600	0.00012500
O	1.63871000	0.63268600	-0.00006800
C	-3.00408600	-0.16563400	-0.00010300
H	-3.61465100	-1.06713900	-0.00079300
H	-3.19632300	0.43537200	0.89166900
H	-3.19590400	0.43649500	-0.89120200
C	3.00408800	0.16563500	-0.00008900
H	3.61465200	1.06714100	-0.00019800
H	3.19615700	-0.43585100	0.89139200
H	3.19607600	-0.43601300	-0.89147900

MB

C	2.13334600	-1.37852900	0.00035200
C	0.75197200	-1.18090700	0.00034000
C	0.23140000	0.12223200	0.00001600
C	1.10318700	1.22125100	-0.00033900
C	2.48260800	1.01897000	-0.00040800
C	2.99956100	-0.28083900	-0.00005000
H	2.53381600	-2.38798100	0.00065600
H	0.07520000	-2.02749500	0.00058500
H	0.68235700	2.22115400	-0.00053800
H	3.15419200	1.87236300	-0.00073700
H	4.07448500	-0.43769400	-0.00007700
C	-1.23490000	0.39379500	0.00015700
O	-1.73029000	1.50636700	0.00084900
O	-1.97573800	-0.73893200	-0.00064100
C	-3.40311400	-0.55258500	-0.00024700
H	-3.71546400	-0.00074600	-0.89005100
H	-3.82556600	-1.55710300	-0.00107800
H	-3.71515000	-0.00230800	0.89064300

2H^{s}

Ru	0.00000400	-0.26297900	0.00011300
P	1.74182200	1.14840200	-0.05397200
P	-1.74183900	1.14839700	0.05404600
N	1.62999600	-1.81845600	-0.17021600
H	1.46363800	-2.17431600	-1.10965300
H	1.48613000	-2.57965000	0.48933800
N	-1.63004900	-1.81843300	0.17073600
H	-1.46365100	-2.17341700	1.11050000
H	-1.48620200	-2.58022500	-0.48813000
C	3.24706600	0.03313300	-0.03596700
C	4.57592900	0.47851300	0.03526600
H	4.78612500	1.54457300	0.05504600
C	5.63869000	-0.42494600	0.09536100
H	6.66021100	-0.06042100	0.15787000
C	5.38238100	-1.79967900	0.08195100
H	6.20165300	-2.51100600	0.13415100
C	4.06669300	-2.25834800	-0.00320100
H	3.86144700	-3.32654300	-0.02462800
C	3.00651900	-1.34646500	-0.06586600
C	-3.24707100	0.03313400	0.03593100
C	-4.57591500	0.47851400	-0.03563500
H	-4.78610200	1.54457400	-0.05552500
C	-5.63866300	-0.42494700	-0.09597300

H	-6.66016800	-0.06042500	-0.15876000
C	-5.38235200	-1.79967800	-0.08249400
H	-6.20160800	-2.51100800	-0.13490900
C	-4.06668800	-2.25834900	0.00304500
H	-3.86145300	-3.32654400	0.02457100
C	-3.00653000	-1.34646600	0.06597500
H	0.11457900	-0.35076300	1.71486400
H	-0.11455700	-0.35068200	-1.71456200
C	-2.11604900	2.27035500	1.50608800
H	-1.35346900	3.05462400	1.54550400
H	-3.10271500	2.73982800	1.43191100
H	-2.04882200	1.68201500	2.42370400
C	-2.11727800	2.31778700	-1.35697700
H	-3.09636300	2.79854400	-1.26088600
H	-1.34593400	3.09415900	-1.37694200
H	-2.06256800	1.75523300	-2.29109800
C	2.11736300	2.31786600	1.35696400
H	1.34650000	3.09473400	1.37642900
H	2.06190500	1.75552200	2.29116500
H	3.09678400	2.79799600	1.26119300
C	2.11596200	2.27028900	-1.50609100
H	1.35359300	3.05476700	-1.54532400
H	3.10277700	2.73948400	-1.43214400
H	2.04837100	1.68195000	-2.42368000

2H

Ru	-0.00002600	1.47897500	0.00059800
P	-1.77043400	0.07383500	-0.01856500
P	1.77045400	0.07387000	0.01870800
N	-1.62797000	3.01313800	-0.24081900
H	-1.59033400	3.14486100	-1.25060600
H	-1.40526600	3.90390400	0.19748000
N	1.62796100	3.01295300	0.24312200
H	1.59078700	3.14393100	1.25302400
H	1.40504100	3.90407100	-0.19435100
C	-3.21924700	1.22310900	0.30898400
C	-4.50699700	0.82480000	0.69553600
H	-4.72624400	-0.23013600	0.82356700
C	-5.50511800	1.76540500	0.95734900
H	-6.49121000	1.43578900	1.27199500
C	-5.22846500	3.12913300	0.82442900
H	-5.99706300	3.86832700	1.03096600
C	-3.95641100	3.54131400	0.42285600
H	-3.73620000	4.60054900	0.30820800
C	-2.95978100	2.59348400	0.16816000
C	3.21909300	1.22340800	-0.30863700
C	4.50665700	0.82543200	-0.69612700
H	4.72586400	-0.22940000	-0.82509300
C	5.50461300	1.76627300	-0.95775900
H	6.49056200	1.43692100	-1.27312800
C	5.22796300	3.12988800	-0.82374000
H	5.99642000	3.86926800	-1.03013700
C	3.95607600	3.54173000	-0.42126600
H	3.73587000	4.60087700	-0.30580100
C	2.95962600	2.59367900	-0.16675000
C	-2.36092300	-0.83003400	-1.56038600
C	-3.47670200	-1.68505400	-1.57867000
H	-4.01395600	-1.90476200	-0.66140300
C	-3.90288200	-2.27523800	-2.77012600
H	-4.76840600	-2.93269800	-2.76689600
C	-3.21514800	-2.02343100	-3.96289000
H	-3.54508400	-2.48520600	-4.88978600
C	-2.10243500	-1.18014000	-3.95317500
H	-1.55798900	-0.98430700	-4.87321800

C	-1.67857300	-0.58592600	-2.75912600
H	-0.81882500	0.08009700	-2.73810800
C	-1.73346700	-2.59100000	1.02911900
H	-1.50571600	-2.91041700	0.01705400
C	-1.78773800	-3.54478500	2.05136100
H	-1.59779200	-4.58979400	1.82115300
C	-2.08860300	-3.15733500	3.35879500
H	-2.13641300	-3.89809300	4.15223800
C	-2.31985500	-1.80667600	3.64081700
H	-2.54637400	-1.49264700	4.65638300
C	-2.25144300	-0.85378200	2.62283900
H	-2.39887600	0.19565500	2.85843700
C	-1.97268800	-1.23527600	1.30031400
C	1.97261600	-1.23457600	-1.30083900
C	2.25041400	-0.85233800	-2.62334700
H	2.39709300	0.19729400	-2.85856800
C	2.31879300	-1.80474900	-3.64178200
H	2.54454100	-1.49013900	-4.65733900
C	2.08848500	-3.15566200	-3.36022100
H	2.13626800	-3.89604400	-4.15401700
C	1.78858900	-3.54386700	-2.05278600
H	1.59938900	-4.58909100	-1.82294100
C	1.73433500	-2.59056400	-1.03009600
H	1.50731000	-2.91053100	-0.01804600
C	2.36125800	-0.83064000	1.56002400
C	3.47756800	-1.68497700	1.57793200
H	4.01521100	-1.90364400	0.66064300
C	3.90383800	-2.27578900	2.76904300
H	4.76977400	-2.93270500	2.76552000
C	3.21568000	-2.02529200	3.96183900
H	3.54569500	-2.48755300	4.88846600
C	2.10247200	-1.18265300	3.95250300
H	1.55771500	-0.98780300	4.87257100
C	1.67852100	-0.58780000	2.75880000
H	0.81843700	0.07779900	2.73809700
H	-0.23419500	1.60035700	1.69351300
H	0.23414200	1.60172000	-1.69231500

3H^{s}

Ru	-0.00000400	-0.45802200	0.00010600
P	1.68195900	1.05312900	-0.20901100
P	-1.68196600	1.05317300	0.20914400
N	-1.47249100	-2.11727200	0.34054500
C	3.67028900	-0.99308400	-0.50024100
C	4.94791900	-1.54663900	-0.33204200
C	5.95471900	-0.86454900	0.35368000
C	5.67819600	0.39142700	0.89146900
C	4.40835800	0.95294100	0.72807700
C	3.38815700	0.28851000	0.02906300
C	-2.62798100	-1.81477700	1.22589400
C	-3.67033900	-0.99299700	0.50014800
C	-4.94793300	-1.54657100	0.33176800
C	-5.95459700	-0.86456900	-0.35424600
C	-5.67796400	0.39132600	-0.89216000
C	-4.40815700	0.95286800	-0.72859000
C	-3.38811200	0.28853600	-0.02926400
C	2.62781400	-1.81496900	-1.22573200
N	1.47242800	-2.11724600	-0.34017600
H	-5.15402300	-2.53197700	0.74396300
H	-6.93657300	-1.31393900	-0.47188100
H	-6.44279900	0.93722800	-1.43797900
H	-4.22288700	1.93053000	-1.15839500
H	4.22318100	1.93067700	1.15775700
H	6.44314400	0.93741500	1.43704300

H	6.93671300	-1.31391100	0.47119900
H	5.15392700	-2.53209400	-0.74415700
H	2.21578900	-1.29089200	-2.09359800
H	3.10061500	-2.74046700	-1.58116700
H	-3.10084100	-2.74019300	1.58148000
H	-2.21604200	-1.29056800	2.09372000
H	-0.92561000	-2.85384000	0.78132800
H	-1.81274900	-2.46966600	-0.55280600
H	0.29285000	-0.54424400	1.69426100
H	-0.29281700	-0.54383500	-1.69402700
C	-1.98691900	1.93832300	1.83983000
H	-2.11141200	1.19173800	2.62756100
H	-1.10461600	2.53529000	2.08652600
H	-2.87127300	2.58319700	1.79944100
C	-1.75882600	2.51933300	-0.94975500
H	-1.81201800	2.15904600	-1.97975300
H	-2.59331500	3.19467900	-0.73784000
H	-0.83126500	3.08482500	-0.83486500
C	1.98661400	1.93837700	-1.83968000
H	2.11099400	1.19182100	-2.62745500
H	1.10425900	2.53533100	-2.08620600
H	2.87095800	2.58327200	-1.79940400
C	1.75895700	2.51916700	0.95005300
H	2.59328300	3.19467900	0.73802800
H	0.83126100	3.08450300	0.83549300
H	1.81246000	2.15872800	1.97998300
H	0.92562700	-2.85413100	-0.78052200
H	1.81274600	-2.46903800	0.55338200

3H

Ru	0.00011700	-1.53750800	-0.00038300
P	1.74785300	-0.05545900	0.07283700
P	-1.74775800	-0.05548100	-0.07284300
N	-1.48757800	-3.21484000	0.14350400
C	3.64115500	-2.15494800	0.46228200
C	4.76247900	-2.76474500	1.04017800
C	5.51661200	-2.11871700	2.02260000
C	5.14229300	-0.84055600	2.43366600
C	4.02477300	-0.22212600	1.86357800
C	3.25671100	-0.85908300	0.87857600
C	-2.87256200	-2.91408700	0.59426600
C	-3.64097400	-2.15511100	-0.46252900
C	-4.76225800	-2.76491500	-1.04048600
C	-5.51652400	-2.11876400	-2.02273400
C	-5.14237600	-0.84047000	-2.43353200
C	-4.02490400	-0.22202000	-1.86335600
C	-3.25672600	-0.85908600	-0.87853200
C	2.87310300	-2.91370300	-0.59494400
N	1.48789800	-3.21447800	-0.14504600
H	-5.04962000	-3.76113500	-0.71082300
H	-6.38272000	-2.60945900	-2.45742300
H	-5.71615800	-0.31719400	-3.19375200
H	-3.75557300	0.77370800	-2.19647800
H	3.75527900	0.77348900	2.19690100
H	5.71598100	-0.31738500	3.19402800
H	6.38285200	-2.60939700	2.45721800
H	5.04998000	-3.76086400	0.71033400
H	2.78217100	-2.33424500	-1.51801800
H	3.41465100	-3.83960300	-0.83010900
H	-3.41403700	-3.84000500	0.82951400
H	-2.78108400	-2.33482600	1.51738300
H	-1.06967100	-3.85125900	0.81857900
H	-1.53139000	-3.68961200	-0.75687300
H	0.12729700	-1.64901200	1.70615200

H -0.12693300 -1.64795700 -1.70684200

H 1.07013700 -3.85028100 -0.82078000

H 1.53107500 -3.68977200 0.75507600

C -2.61677100 0.54810800 1.49348400

C -3.82247900 1.27106900 1.45641300

C -2.08518700 0.19442100 2.73925100

C -4.46597600 1.64794800 2.63683300

H -4.26893500 1.53905500 0.50341800

C -2.73065600 0.56669300 3.92443100

H -1.17277300 -0.39712000 2.75799400

C -3.91957800 1.29731100 3.87715700

H -5.39574200 2.20880100 2.58858700

H -2.30393900 0.28059700 4.88258800

H -4.42276500 1.58657900 4.79604500

C -1.57341200 1.51604300 -1.07309500

C -1.22264000 1.39751900 -2.42965600

C -1.71410400 2.80014700 -0.52848200

C -1.05129400 2.53046700 -3.22603700

H -1.07847900 0.40762400 -2.85212000

C -1.53068400 3.93730000 -1.32384700

H -1.95842800 2.92523900 0.52032600

C -1.20700400 3.80746300 -2.67540800

H -0.78586700 2.41660300 -4.27357800

H -1.63934700 4.92313500 -0.87957000

H -1.06812100 4.69050900 -3.29332300

C 1.57303200 1.51579600 1.07342500

C 1.71257000 2.79999700 0.52871500

C 1.22297300 1.39707900 2.43014200

C 1.52877700 3.93704600 1.32413700

H 1.95623800 2.92525500 -0.52022700

C 1.05126500 2.52993600 3.22657700

H 1.07962200 0.40712200 2.85273500

C 1.20586100 3.80702600 2.67586300

H 1.63654600 4.92293200 0.87975600

H 0.78639700 2.41590000 4.27424200

H 1.06670200 4.68997700 3.29384800

C 2.61712500 0.54859700 -1.49310000

C 3.82269900 1.27177100 -1.45568100

C 2.08573800 0.19524400 -2.73904700

C 4.46623700 1.64917100 -2.63590300

H 4.26902600 1.53951100 -0.50256000

C 2.73127500 0.56802500 -3.92405000

H 1.17336400 -0.39636600 -2.75814000

C 3.92003400 1.29886100 -3.87641200

H 5.39588300 2.21020000 -2.58736100

H 2.30469000 0.28215100 -4.88233100

H 4.42325300 1.58857100 -4.79514300

2H^{s}-TS1$_{MB}$

Ru -0.36367700 -0.71701000 -0.34078400

P 1.23006900 -1.85981700 0.77813900

P -2.23199700 -1.26463100 0.79317000

N 1.36616700 -0.15855500 -1.65411600

H 1.49599400 0.84843100 -1.53384700

H 1.09637500 -0.31318800 -2.62349100

N -1.79253500 0.42817900 -1.60382100

H -1.66179700 0.22042200 -2.59179200

C 2.76086000 -1.65466400 -0.27454800

C 3.99387700 -2.27806400 -0.02987000

H 4.11156200 -2.91790500 0.84076300

C 5.07561800 -2.09998900 -0.89404000

H 6.02128600 -2.59486200 -0.69154100

C 4.93530900 -1.28683400 -2.02307000

H 5.77019700 -1.14434500 -2.70315100

C	3.71825800	-0.65134300	-2.27333100
H	3.60602600	-0.00759700	-3.14270200
C	2.63788700	-0.83329600	-1.40254700
C	-3.60096800	-0.41826000	-0.15520200
C	-4.96561700	-0.49409300	0.16240300
H	-5.28679500	-1.05806200	1.03445000
C	-5.92531900	0.13648300	-0.63143100
H	-6.97826700	0.06463600	-0.37389900
C	-5.52387200	0.85694800	-1.76122200
H	-6.26264400	1.35108400	-2.38581300
C	-4.16900600	0.94803000	-2.08387300
H	-3.84993700	1.52119300	-2.95107600
C	-3.20827400	0.31462300	-1.28504000
H	-0.58171500	-2.06929700	-1.27740800
C	-2.85169900	-3.02637500	0.86863600
H	-2.16355000	-3.61574700	1.48217400
H	-3.85619300	-3.09537900	1.29825600
H	-2.84958600	-3.43713300	-0.14298900
C	-2.54664500	-0.74353600	2.56249400
H	-3.58437500	-0.90760100	2.87133200
H	-1.88957800	-1.33030100	3.21261100
H	-2.28577000	0.31027500	2.67733100
C	1.14636000	-3.71624800	0.97932500
H	0.33284400	-3.95530700	1.67129900
H	0.91671900	-4.16120600	0.00896900
H	2.07645100	-4.13761200	1.37374900
C	1.84707500	-1.37983300	2.47725300
H	1.04150000	-1.54957100	3.19885100
H	2.72630900	-1.95674600	2.78264400
H	2.08888900	-0.31514200	2.47315600
C	3.39569300	2.65522300	1.88478500
C	2.01660800	2.50657300	1.74120800
C	1.41155500	2.69993000	0.49064900
C	2.20496300	3.06385300	-0.60530200
C	3.59002100	3.20544900	-0.46134400
C	4.18961500	2.99778700	0.78234000
H	3.85594300	2.50540600	2.85793400
H	1.39697100	2.24202400	2.59116100
H	1.71687300	3.26611300	-1.55451400
H	4.19577000	3.49144500	-1.31720400
H	5.26429300	3.10853900	0.89715600
C	-0.07975700	2.58314200	0.29005400
O	-0.60159700	2.80319300	-0.82861200
O	-0.73865700	2.91847000	1.45493600
C	-2.15121200	3.07573800	1.34127400
H	-2.62712700	2.12547100	1.07509800
H	-2.49392800	3.39999200	2.32571000
H	-2.40299500	3.82397000	0.58439800
H	-1.47399300	1.40634400	-1.44332300
H	-0.13643600	0.86867400	0.49794500

2H^{s}-TS1$_{MB}$′

Ru	0.28236000	-0.69936400	-0.37012400
P	2.15991200	-1.29485000	0.72878200
P	-1.27683900	-1.91477500	0.70962600
N	1.72284300	0.58273400	-1.52988900
H	1.49261100	1.49715600	-1.11674300
H	1.52770900	0.62251100	-2.52765500
N	-1.48872000	-0.05549000	-1.59708600
H	-1.24958200	-0.16840800	-2.58035100
C	3.53379800	-0.48661300	-0.24958900
C	4.90387900	-0.67921800	-0.01854100
H	5.22835800	-1.29582400	0.81559100
C	5.86438700	-0.10669800	-0.85501600

H 6.92086700 -0.27471900 -0.66626900
C 5.45879400 0.67418900 -1.94169500
H 6.19730700 1.11739800 -2.60358700
C 4.09921300 0.89347600 -2.17132000
H 3.77991200 1.51752500 -3.00316000
C 3.14084400 0.32180200 -1.32607500
C -2.84492400 -1.62872200 -0.26589200
C -4.07508400 -2.25445600 -0.01231200
H -4.16597400 -2.94944600 0.81820300
C -5.18932700 -2.00573200 -0.81559600
H -6.13251000 -2.50271500 -0.60693600
C -5.08523800 -1.11807000 -1.89122700
H -5.94587100 -0.91959100 -2.52350200
C -3.87099900 -0.48046700 -2.14933900
H -3.78671800 0.22102300 -2.97611900
C -2.75803200 -0.73418200 -1.33964900
H 0.49817000 -1.98281200 -1.40904100
C -1.20562100 -3.78100000 0.78165900
H -0.37674400 -4.07516000 1.43260900
H -2.12930200 -4.21874200 1.17339900
H -1.00861200 -4.16076400 -0.22300400
C -1.81244300 -1.53830900 2.45966300
H -2.70570500 -2.09949200 2.75292300
H -0.99193300 -1.79475800 3.13693100
H -2.00131900 -0.46659200 2.54560200
C 2.74920000 -3.06575800 0.80449800
H 2.05008800 -3.64056300 1.41911600
H 2.74235500 -3.47920700 -0.20622200
H 3.75014700 -3.15397300 1.23854900
C 2.47922500 -0.74920700 2.48469700
H 1.88623500 -1.38420200 3.15142600
H 3.53464700 -0.83662800 2.76317700
H 2.13400300 0.28071200 2.60859900
C -2.79899400 3.56768300 -0.86295000
C -1.48859400 3.22339400 -0.50944600
C -1.21446300 2.68122900 0.75628700
C -2.26778000 2.51042400 1.66524700
C -3.57373900 2.85098900 1.31033800
C -3.84691400 3.37716600 0.04248500
H -2.99670500 4.00152100 -1.84012700
H -0.67087800 3.40903100 -1.19805300
H -2.03656600 2.12528200 2.65311900
H -4.37991800 2.71492900 2.02635300
H -4.86301300 3.64671400 -0.23170700
C 0.18288100 2.32216500 1.21518300
O 0.47551200 2.23500400 2.41284000
O 1.13823900 2.83475000 0.29373000
C 2.41493100 3.13184800 0.87217900
H 2.92628400 2.22326800 1.20381900
H 2.99351100 3.61317300 0.08055200
H 2.30479500 3.80423500 1.72676700
H -1.61484200 0.94516800 -1.42517600
H 0.10037700 0.77600900 0.63495200

2H^{s}-TS2$_{MB}$

Ru -0.25013600 -1.03645600 -0.24879200
P 1.59778500 -1.49216500 0.98368200
P -1.93344200 -1.09742800 1.28158100
N 1.24901600 -0.84588300 -1.88745900
H 1.15361700 0.17540500 -1.97174600
H 0.94844200 -1.26118800 -2.76668100
N -1.89807200 -0.38081200 -1.56866300
H -1.88207500 -0.59957700 -2.56237400
C 2.97746700 -1.53602600 -0.27789400

C	4.31039100	-1.87209800	0.00102100
H	4.60605700	-2.10778400	1.01999200
C	5.26788300	-1.92446900	-1.01383700
H	6.29355100	-2.19603600	-0.78102000
C	4.89867600	-1.63253600	-2.33058800
H	5.63518200	-1.67456500	-3.12781000
C	3.57995600	-1.27838600	-2.62133300
H	3.29126600	-1.03490500	-3.64120700
C	2.62430900	-1.22623100	-1.59991100
C	-3.44904700	-0.69083100	0.29163000
C	-4.75600000	-0.67596300	0.79746600
H	-4.92725500	-0.86357800	1.85512300
C	-5.85154900	-0.45125500	-0.03816900
H	-6.86008500	-0.45406400	0.36444200
C	-5.63120500	-0.23912300	-1.40500600
H	-6.47353500	-0.07490900	-2.07204500
C	-4.33616800	-0.22696100	-1.91915200
H	-4.17351700	-0.04235500	-2.97895000
C	-3.22233600	-0.43752500	-1.08099400
H	-0.41930600	-2.60223900	-0.34255200
C	-2.38957600	-2.68543900	2.14828400
H	-1.57963800	-2.96445100	2.82938500
H	-3.31754000	-2.58774300	2.72027800
H	-2.50074400	-3.47236400	1.39930600
C	-2.00645900	0.10264200	2.71260400
H	-2.95732100	0.03732000	3.25126600
H	-1.18742100	-0.11693500	3.40440500
H	-1.87482900	1.11884600	2.33411400
C	1.81228500	-3.12182000	1.87024700
H	1.08844900	-3.16634900	2.68990600
H	1.59448200	-3.93422900	1.17349000
H	2.81878100	-3.24548700	2.28190500
C	2.26900000	-0.31580900	2.27157500
H	1.59422100	-0.31889200	3.13338000
H	3.27138500	-0.60216400	2.60628700
H	2.29676900	0.69505300	1.85865100
C	1.58060700	3.88938100	2.47242600
C	0.50658500	3.48427900	1.67124900
C	0.71496000	3.06627300	0.35375000
C	2.01938700	3.06812300	-0.16016100
C	3.09346500	3.47392300	0.63311100
C	2.87786500	3.88474100	1.95481400
H	1.40207800	4.21087900	3.49510400
H	-0.50333100	3.49988200	2.07485100
H	2.18294600	2.75831800	-1.18760700
H	4.09948200	3.47500000	0.22162300
H	3.71431300	4.20157700	2.57185200
C	-0.45071300	2.63293500	-0.51856000
O	-0.26307700	1.35892800	-1.03378400
O	-0.55296500	3.61922200	-1.55153200
C	-1.70269100	3.48027100	-2.36905800
H	-2.62529100	3.48943400	-1.76732700
H	-1.71317200	4.33649100	-3.04811600
H	-1.67570100	2.55441000	-2.95888700
H	-1.24323300	0.69441500	-1.36955200
H	-1.38702600	2.67381600	0.07272200

2H^{s}-TS3$_{MB}$

Ru	0.57022500	-0.90344300	0.23340000
P	-0.99304300	-1.94344100	-1.02535700
P	2.35956200	-0.74652500	-1.17468500
N	-1.03510100	-0.95548300	1.77796400
H	-1.19756200	0.03928100	1.99074700
H	-0.65136000	-1.39292600	2.61384700

N	1.92824700	0.27559400	1.52790900
H	1.80448400	0.27928000	2.53758300
C	-2.45825500	-2.11066200	0.12601500
C	-3.68031400	-2.71274700	-0.21154500
H	-3.82690000	-3.11175000	-1.21189000
C	-4.71532200	-2.81464900	0.71946500
H	-5.65360500	-3.28630900	0.44147900
C	-4.53714600	-2.30911000	2.01145600
H	-5.33574500	-2.38463400	2.74389700
C	-3.32957000	-1.70203700	2.35977300
H	-3.18771200	-1.30104500	3.36063600
C	-2.29367600	-1.60076600	1.42232800
C	3.70230400	-0.03926000	-0.10787700
C	5.04476800	0.09191300	-0.48468600
H	5.35363800	-0.19265600	-1.48822600
C	6.00593800	0.55657100	0.41604000
H	7.04628100	0.63971700	0.11585800
C	5.61024700	0.89704900	1.71552500
H	6.34710500	1.24968900	2.43271200
C	4.27398300	0.79595600	2.09849700
H	3.97354900	1.08105800	3.10467500
C	3.29552900	0.33802900	1.19335600
H	1.06392800	-2.35420900	0.61592700
C	3.14787600	-2.25246000	-1.93894200
H	2.44546200	-2.69701600	-2.65065300
H	4.07579400	-2.00714900	-2.46435700
H	3.35343300	-2.97980600	-1.15043200
C	2.29657800	0.41696600	-2.63307900
H	3.28887100	0.57485500	-3.06798600
H	1.62980900	0.00474900	-3.39687200
H	1.88596800	1.36927600	-2.28906400
C	-0.77943600	-3.69551700	-1.63962800
H	0.00680600	-3.70549600	-2.40062100
H	-0.45909600	-4.32355400	-0.80539600
H	-1.69879300	-4.09914100	-2.07579000
C	-1.75119900	-1.15423000	-2.54199800
H	-1.00005700	-1.14784700	-3.33813500
H	-2.63598900	-1.69368000	-2.89602200
H	-2.01610400	-0.11928700	-2.31428700
C	-2.14434100	4.72378600	-1.91326500
C	-1.26920100	4.26713500	-0.92239000
C	-1.50103400	3.05307000	-0.26624600
C	-2.63494700	2.30370700	-0.60808800
C	-3.51482200	2.75851000	-1.59300900
C	-3.27085100	3.96921300	-2.25151000
H	-1.94910500	5.66744700	-2.41566200
H	-0.39360900	4.85689800	-0.65969700
H	-2.82886200	1.36872800	-0.09136000
H	-4.39424100	2.17120200	-1.84475700
H	-3.95657400	4.32279700	-3.01662600
C	-0.50079900	2.54940500	0.76892300
O	0.33872500	1.61913800	0.24124600
O	-1.19982500	1.95973400	1.91790500
C	-1.86135200	2.90192300	2.75073000
H	-2.68068200	3.40814900	2.22327300
H	-2.27189900	2.35112700	3.60124800
H	-1.15846100	3.66129300	3.12489700
H	0.05613300	3.41753500	1.17633100
H	1.20441000	1.17191500	0.99233100

2H^{s}-TS4$_{MB}$

Ru	0.49782100	-1.13931600	0.35170700
P	-1.05615000	-2.39064800	-0.74025900
P	2.42966500	-1.31550700	-0.84653700

N	-1.16884300	-0.73710900	1.71556400
H	-0.99556000	0.47038700	1.92606700
H	-0.97856100	-1.18295800	2.61271400
N	1.75842100	0.15334300	1.64679200
H	1.92681800	-0.42746200	2.46691000
C	-2.63579500	-1.92687800	0.12751700
C	-3.92072700	-2.31264000	-0.28249800
H	-4.04509500	-2.92225100	-1.17468600
C	-5.05086800	-1.92908600	0.44162500
H	-6.04088700	-2.23174400	0.11215900
C	-4.89436400	-1.15018300	1.59437000
H	-5.76634900	-0.84156000	2.16511900
C	-3.62190300	-0.76040900	2.01169100
H	-3.50083300	-0.14621900	2.90045900
C	-2.47705300	-1.14290500	1.28914300
C	3.53377800	-0.02518500	-0.05037500
C	4.78275300	0.38159700	-0.54411800
H	5.19385400	-0.09249000	-1.43176000
C	5.50752400	1.40021500	0.07835800
H	6.46938100	1.70711700	-0.32275800
C	4.98552800	2.02745200	1.21426300
H	5.53846400	2.82600600	1.70088800
C	3.75214900	1.62321600	1.72849600
H	3.34348700	2.09911700	2.61633800
C	3.02945400	0.59703500	1.10559400
H	0.93570300	-2.40708800	1.21221400
C	3.50794300	-2.84134700	-0.83530400
H	2.98930700	-3.63797300	-1.37768900
H	4.48285600	-2.66463300	-1.30111300
H	3.64700900	-3.16579300	0.19846200
C	2.52422200	-0.85034200	-2.65573000
H	3.55229100	-0.75853700	-3.02003900
H	2.01281900	-1.62435800	-3.23644700
H	1.99593700	0.09456100	-2.80384600
C	-1.06922500	-4.25620600	-0.69790900
H	-0.21936600	-4.63128700	-1.27672100
H	-0.95363500	-4.58300000	0.33793400
H	-1.99638800	-4.66643500	-1.11086300
C	-1.45029400	-2.07993800	-2.53911600
H	-0.63250500	-2.47120900	-3.15218300
H	-2.38340900	-2.56015000	-2.85038900
H	-1.52103500	-1.00144000	-2.69760000
C	-1.59789000	5.38855900	-1.14032700
C	-0.76970300	4.35278700	-0.70581800
C	-1.10881400	3.01719000	-0.97194500
C	-2.29232200	2.72410300	-1.67070900
C	-3.11806100	3.75868000	-2.10359100
C	-2.77131800	5.09118200	-1.84073900
H	-1.33349700	6.42131100	-0.93260200
H	0.13954000	4.57170900	-0.15147700
H	-2.54718300	1.68576700	-1.85679500
H	-4.03414900	3.53319800	-2.64199100
H	-3.41847600	5.89560900	-2.17933300
C	-0.20531200	1.94495000	-0.51752000
O	-0.38213100	0.75360000	-0.77707600
O	-0.52006700	1.63857700	2.12198700
C	-1.15294100	2.37070800	3.13239800
H	-1.96193600	3.01364800	2.73993500
H	-1.60823500	1.71839300	3.90359600
H	-0.44281800	3.03129400	3.65913900
H	0.68375300	2.27477600	0.03712500
H	1.13181200	0.92952200	1.95374800

2H^{s}-TS4$_{MB}$′

Ru	-0.00300700	-0.26119100	-0.01222100
P	-1.75044300	1.15321700	0.06774900
P	1.78728200	1.15238400	-0.05776600
N	-1.61713300	-1.81292600	-0.17825700
H	-1.47623500	-2.55752200	0.50162600
H	-1.43372600	-2.20549100	-1.10054300
N	1.64889400	-1.78206600	0.24626700
H	1.53755300	-2.78720500	0.16574200
C	-3.24701300	0.03106300	-0.01768200
C	-4.57909100	0.47041500	0.03238800
H	-4.79408100	1.53343300	0.10218600
C	-5.63957600	-0.43671900	0.00103700
H	-6.66399100	-0.07794000	0.04372900
C	-5.37795400	-1.80815100	-0.08079300
H	-6.19623400	-2.52200500	-0.10246300
C	-4.05882900	-2.26047100	-0.13836500
H	-3.84914500	-3.32547400	-0.20884700
C	-2.99996900	-1.34538600	-0.10850300
C	3.25208100	0.02126100	-0.00011900
C	4.58347000	0.45324700	-0.08057500
H	4.79821000	1.51696500	-0.15885900
C	5.64402600	-0.45308900	-0.08533400
H	6.66889900	-0.10282000	-0.16195500
C	5.36084200	-1.82443400	-0.00126700
H	6.17286700	-2.54729500	-0.01202900
C	4.04928500	-2.27418800	0.10376500
H	3.84471700	-3.33992900	0.18338300
C	2.96452600	-1.36482600	0.11341300
H	-0.09614300	-0.12227000	-1.64303100
C	2.10020600	2.23072100	-1.54718000
H	1.32313100	2.99955700	-1.60216600
H	3.07956600	2.71714400	-1.49861400
H	2.03955500	1.60938300	-2.44278700
C	2.11738000	2.37257500	1.31880300
H	3.10856500	2.82810900	1.22608900
H	1.36071100	3.16350400	1.29334400
H	2.04515600	1.84910400	2.27488300
C	-2.10844700	2.39992700	-1.27468600
H	-1.33466300	3.17326800	-1.24706700
H	-2.05398000	1.89444000	-2.24109300
H	-3.08743600	2.87474000	-1.15523600
C	-2.12052400	2.17456300	1.58989100
H	-1.34274500	2.93734000	1.69301200
H	-3.09689800	2.66734500	1.53978800
H	-2.07887300	1.52216500	2.46524700
H	0.26334700	-0.67018700	1.78967600
H	0.97680500	-1.27409900	1.25913200

2H^{s}-TS5$_{MB}$

Ru	0.00000200	-0.08835900	-0.23796100
P	1.79501700	-1.41357300	0.17358800
P	-1.79501400	-1.41357000	0.17360100
N	1.56920700	1.41280200	-0.72049900
H	1.27460300	2.18369000	-0.07335500
H	1.44499000	1.75106100	-1.67286200
N	-1.56920600	1.41280300	-0.72045900
H	-1.44496100	1.75109100	-1.67280900
C	3.25310900	-0.28417600	-0.11557400
C	4.59594700	-0.65137800	0.06172700
H	4.84369300	-1.65964400	0.38352000
C	5.62696700	0.25989900	-0.17144300
H	6.66145500	-0.04012400	-0.03045700
C	5.32051000	1.56051000	-0.58567500
H	6.11558300	2.27851300	-0.76569400

C	3.98952900	1.93952200	-0.76420000
H	3.74451200	2.95126800	-1.07772100
C	2.95544900	1.02258600	-0.53255800
C	-3.25310500	-0.28415200	-0.11549400
C	-4.59594100	-0.65134400	0.06184100
H	-4.84368400	-1.65958700	0.38370600
C	-5.62696500	0.25991200	-0.17139500
H	-6.66145100	-0.04010200	-0.03037800
C	-5.32051400	1.56048900	-0.58574000
H	-6.11559000	2.27847100	-0.76582300
C	-3.98953400	1.93949500	-0.76428800
H	-3.74452300	2.95122000	-1.07788400
C	-2.95545100	1.02258500	-0.53256200
H	-0.00002600	-0.64261300	-1.76086200
C	-2.19934100	-2.89637000	-0.88543000
H	-1.44035500	-3.66580400	-0.71284400
H	-3.18626300	-3.31191300	-0.65844300
H	-2.15486700	-2.59829900	-1.93514500
C	-2.14690100	-2.10057700	1.87477500
H	-3.13910100	-2.55750200	1.94529700
H	-1.39171100	-2.85776400	2.10743100
H	-2.06128500	-1.29186800	2.60440000
C	2.19930500	-2.89645000	-0.88534800
H	1.44018800	-3.66577400	-0.71285700
H	2.15502400	-2.59839900	-1.93507800
H	3.18613800	-3.31212200	-0.65821000
C	2.14692800	-2.10044800	1.87481100
H	1.39169000	-2.85755100	2.10758100
H	3.13909700	-2.55744100	1.94533300
H	2.06140800	-1.29165700	2.60435600
O	0.00000700	2.84149600	0.96276100
C	-0.00002600	3.67917500	2.08262400
H	-0.88732000	4.33725300	2.10878200
H	-0.00051100	3.10566600	3.03067000
H	0.88772400	4.33661400	2.10930900
H	-1.27461100	2.18365100	-0.07328100
H	0.00003800	1.43681000	1.31498500
H	0.00010200	0.57093800	1.53898900

2H^{s}-IN2$_{MB}$

Ru	-0.45157800	-0.89748200	-0.23465500
P	1.33897600	-1.54030800	1.00256300
P	-2.10389700	-0.74196900	1.30372000
N	1.06825000	-0.88875200	-1.87173400
H	1.05092800	0.15141100	-1.83036700
H	0.75868900	-1.18129600	-2.79542300
N	-2.09008000	-0.25178300	-1.61173200
H	-2.09020400	-0.62918200	-2.55588400
C	2.69981200	-1.75703100	-0.25824700
C	3.98290000	-2.24516000	0.02629600
H	4.25254500	-2.49187900	1.04992100
C	4.92102200	-2.44079700	-0.98938900
H	5.90727000	-2.82957700	-0.75277500
C	4.57986100	-2.14172800	-2.31201100
H	5.29973800	-2.29536700	-3.11086700
C	3.31360400	-1.63396200	-2.60786300
H	3.05227300	-1.38080600	-3.63282000
C	2.37730000	-1.43429800	-1.58581300
C	-3.62922600	-0.36306100	0.29504100
C	-4.92967900	-0.27182200	0.81145300
H	-5.09636700	-0.38634900	1.87951200
C	-6.02514200	-0.05901600	-0.02812200
H	-7.02747100	-0.00312300	0.38652300
C	-5.82375400	0.06980800	-1.40592000

H	-6.66904900	0.22833400	-2.06952900
C	-4.53245300	0.00598000	-1.93202500
H	-4.37170000	0.12659500	-3.00086900
C	-3.43470500	-0.20199800	-1.08706600
H	-0.79120200	-2.45367000	-0.28389800
C	-2.64299800	-2.21255800	2.31769900
H	-1.84164500	-2.46774100	3.01777600
H	-3.55695400	-2.01311000	2.88554200
H	-2.79995800	-3.06041500	1.64754700
C	-2.13694200	0.59862900	2.60697600
H	-3.09940600	0.64231300	3.12688200
H	-1.34588700	0.40189600	3.33709500
H	-1.93496100	1.56078400	2.13045000
C	1.33512000	-3.19403400	1.86918700
H	0.62244700	-3.15319700	2.69867400
H	1.00373600	-3.96023500	1.16544700
H	2.32220100	-3.45272800	2.26496100
C	2.15296300	-0.48569400	2.31022100
H	1.47282900	-0.39811500	3.16338800
H	3.09413900	-0.92656100	2.65411100
H	2.34066200	0.51279300	1.91166500
C	2.66892100	3.55751300	2.24247000
C	1.44414300	3.32016800	1.60734200
C	1.39610300	2.81444900	0.30448100
C	2.60177000	2.55826900	-0.36296500
C	3.82701800	2.79406800	0.26304000
C	3.86543000	3.29361600	1.57113200
H	2.68739200	3.94935800	3.25631000
H	0.51414000	3.53512800	2.12951800
H	2.57082500	2.18157500	-1.38089300
H	4.75381800	2.59349800	-0.26850700
H	4.81911200	3.47726600	2.05877900
C	0.05805100	2.56846300	-0.38843500
O	-0.06860300	1.33622500	-0.95646100
O	-0.03711700	3.63288000	-1.36819800
C	-1.28869200	3.69908400	-2.01879300
H	-2.11813800	3.75535400	-1.29344900
H	-1.28837700	4.61088000	-2.62304400
H	-1.45822900	2.83688400	-2.67924100
H	-1.61910000	0.68102100	-1.61549200
H	-0.75486900	2.75917000	0.34896200

2H^{s}-IN2$_{MB}$′

Ru	0.37415600	-0.75754100	-0.29603000
P	-1.24728500	-1.82619900	0.88010700
P	2.20089200	-1.03321900	1.04010900
N	-1.33090000	-0.39291500	-1.71956900
H	-1.02633000	-0.53551000	-2.68087700
H	-1.54071400	0.60380600	-1.62117900
N	1.86129100	0.31201100	-1.57398800
H	1.56870900	1.29576200	-1.35706500
C	-2.70591800	-1.85505800	-0.28668000
C	-3.90482100	-2.54592800	-0.05493200
H	-4.04189900	-3.09253400	0.87415800
C	-4.92578900	-2.55503600	-1.00666200
H	-5.84449100	-3.10137600	-0.81371600
C	-4.75882800	-1.86302400	-2.21028000
H	-5.54608900	-1.86720900	-2.95839600
C	-3.57863600	-1.15690200	-2.44791700
H	-3.45058400	-0.60266900	-3.37488500
C	-2.55983400	-1.15135000	-1.48904100
C	3.61175200	-0.42598400	-0.01350400
C	4.96773400	-0.52986000	0.32830500
H	5.25248300	-0.95032800	1.28909500

C	5.96536000	-0.11398500	-0.55504800
H	7.01159200	-0.20917400	-0.27928700
C	5.60954800	0.41883600	-1.79812300
H	6.37788400	0.74050900	-2.49535600
C	4.26308800	0.55121900	-2.14065100
H	3.98348100	0.98777800	-3.09677000
C	3.26508100	0.13641800	-1.25018000
H	0.04425100	1.08165500	0.39542300
C	2.34991100	-0.05187600	2.60827700
H	1.71809800	-0.51742100	3.37198800
H	3.38300000	-0.02371300	2.96942600
H	1.97876400	0.96482600	2.41340400
C	2.78736800	-2.72432600	1.56161200
H	3.76531300	-2.68440100	2.05072000
H	2.06404700	-3.14475200	2.26622900
H	2.83896600	-3.37222400	0.68358000
C	-1.98275300	-1.11540100	2.44122400
H	-1.22695900	-1.15280400	3.23181500
H	-2.24750600	-0.06939100	2.26927000
H	-2.86939300	-1.66940800	2.76631400
C	-1.08714300	-3.62324300	1.35444100
H	-0.30567600	-3.71656100	2.11379900
H	-2.01908800	-4.02860100	1.75996600
H	-0.78571000	-4.19578400	0.47464500
H	1.72666000	0.16501000	-2.57217400
H	0.69750100	-2.17276100	-0.96395900
C	-3.44958800	3.29512800	-0.54813300
C	-2.10243500	2.91606100	-0.59237500
C	-1.37561500	2.71018900	0.58963700
C	-2.02036100	2.90795600	1.81777500
C	-3.36656000	3.28066800	1.86599300
C	-4.08903500	3.47041900	0.68244900

H	-3.99456900	3.46782300	-1.47329100
H	-1.58998700	2.83150800	-1.54726200
H	-1.43411900	2.78580500	2.72363300
H	-3.85123500	3.43392600	2.82721800
H	-5.13418000	3.76596400	0.71887700
C	0.09839700	2.25149500	0.61075200
O	0.79257800	2.56404300	1.62923800
O	0.70397700	2.65718000	-0.75159700
C	1.28068800	3.95911200	-0.72188300
H	1.85415400	4.08327600	-1.64666600
H	0.50310700	4.73548700	-0.67923300
H	1.93868800	4.06989900	0.14607100

2H^{s}-IN3$_{MB}$

Ru	-0.20285800	-1.10097800	-0.21887700
P	1.66709600	-1.50506400	1.01427800
P	-1.90422700	-1.22612800	1.29614300
N	1.29161500	-0.88063400	-1.86383800
H	1.23439000	0.13270500	-2.00858800
H	0.96714100	-1.32877900	-2.71857700
N	-1.81960500	-0.52527000	-1.51924500
H	-1.77552400	-0.67013600	-2.52614100
C	3.03166900	-1.58106100	-0.26611900
C	4.36364300	-1.93458000	-0.00287900
H	4.66873500	-2.16901200	1.01353900
C	5.30797300	-2.00604900	-1.02884100
H	6.33232500	-2.29130000	-0.80665000
C	4.92778200	-1.71597800	-2.34279900
H	5.65382800	-1.77305800	-3.14859200
C	3.61027300	-1.34610900	-2.61920700
H	3.31180700	-1.10649200	-3.63725100
C	2.66834000	-1.27674100	-1.58644900

C	-3.40572000	-0.84897200	0.29049300
C	-4.72258100	-0.85232800	0.76703900
H	-4.91300800	-1.04245300	1.82169500
C	-5.80375700	-0.63842200	-0.09020100
H	-6.82123900	-0.64996400	0.28847500
C	-5.54855900	-0.42724500	-1.45397800
H	-6.37753900	-0.27456700	-2.14112100
C	-4.24659400	-0.40599200	-1.94345000
H	-4.06761600	-0.23047300	-3.00272800
C	-3.13520100	-0.59744600	-1.08324200
H	-0.33200700	-2.65842700	-0.33125800
C	-2.30891900	-2.83728800	2.14541000
H	-1.49565500	-3.09904700	2.82948700
H	-3.24433500	-2.77209700	2.70991400
H	-2.39402900	-3.61916500	1.38769200
C	-2.00592800	-0.04818800	2.74516100
H	-2.96990800	-0.12229900	3.25892400
H	-1.20426300	-0.27736000	3.45432600
H	-1.86867500	0.97469800	2.38642800
C	1.93044100	-3.08771300	1.97248400
H	1.22611400	-3.10226200	2.81004500
H	1.71038100	-3.93703000	1.32163900
H	2.94714200	-3.17743800	2.36746300
C	2.35451800	-0.26235700	2.23481400
H	1.69868900	-0.23126100	3.11063400
H	3.36837600	-0.51913200	2.55930100
H	2.35899800	0.72872900	1.77436300
C	1.20580600	4.09759300	2.52702600
C	0.22092500	3.63791200	1.64583500
C	0.53943200	3.33255300	0.31946500
C	1.85901700	3.49915900	-0.12339700
C	2.84368100	3.95699600	0.75318400
C	2.52003600	4.25656400	2.08234200
H	0.94473800	4.33271200	3.55519200
H	-0.80262900	3.52046200	1.99378600
H	2.10493600	3.27597100	-1.15674200
H	3.86346900	4.08551500	0.40047800
H	3.28716700	4.61544100	2.76297500
C	-0.52784700	2.83264600	-0.62964600
O	-0.17665400	1.55026300	-1.11033600
O	-0.63225400	3.77562600	-1.68016700
C	-1.72237800	3.54793400	-2.56451400
H	-2.67652000	3.51012900	-2.01792000
H	-1.74108200	4.38945700	-3.26021000
H	-1.59836600	2.61554300	-3.12996000
H	-0.98303800	0.97915200	-1.32042200
H	-1.49920500	2.76704600	-0.11145500

2H^{s}-IN4$_{MB}$

Ru	-0.81781300	-0.90026900	-0.23242500
P	0.31653000	-2.51542300	0.89758700
P	-2.68703900	-0.53075300	1.02947900
N	0.89765500	-1.07267400	-1.64296500
H	1.31083400	-0.13245900	-1.69444600
H	0.49276100	-1.27265700	-2.55627600
N	-1.65362800	0.72109400	-1.36200200
H	-1.39292200	0.87583400	-2.33396300
C	1.82128300	-2.82881900	-0.17199100
C	2.82737600	-3.76606800	0.11123600
H	2.76192200	-4.37217900	1.01112100
C	3.91570500	-3.93840900	-0.74568400
H	4.68382800	-4.66961100	-0.51011400
C	4.01148300	-3.16697600	-1.90836700
H	4.85425100	-3.29367800	-2.58184300

C 3.02061200 -2.22922300 -2.20262000
H 3.09110800 -1.62354800 -3.10303900
C 1.93062900 -2.05907800 -1.33994400
C -3.63676700 0.69814100 0.03259000
C -4.92499900 1.16168200 0.32340500
H -5.43027600 0.81980500 1.22474300
C -5.58821200 2.04259000 -0.53404200
H -6.59140200 2.38836100 -0.30398300
C -4.93786900 2.45764300 -1.70589300
H -5.44257200 3.13246600 -2.39339000
C -3.65079400 2.02067400 -2.00323300
H -3.15817700 2.36113300 -2.91229800
C -2.95724400 1.13965100 -1.13593800
H -1.60380600 -2.07737200 -0.90072600
C -3.90794500 -1.89631900 1.37580300
H -3.44490700 -2.63192300 2.04059700
H -4.81881300 -1.51506200 1.84743200
H -4.16286100 -2.38645000 0.43356100
C -2.54749600 0.25803300 2.71888200
H -3.52734600 0.56611500 3.09815200
H -2.09914700 -0.45371800 3.41934900
H -1.89701900 1.13343900 2.64572500
C -0.35640300 -4.24383100 1.13780700
H -1.20081800 -4.19528100 1.83209200
H -0.72232900 -4.61464600 0.17761200
H 0.39257400 -4.93435600 1.53824100
C 1.09161800 -2.23563500 2.58124200
H 0.28993100 -2.14709700 3.32116300
H 1.75845100 -3.05231600 2.87708000
H 1.65038800 -1.29639400 2.56851600
C 3.40883700 4.95473600 1.53413700
C 2.44071700 4.48982400 0.63674000
C 2.29032200 3.12031800 0.39973600
C 3.12359200 2.21309500 1.06916400
C 4.09124400 2.67360800 1.96249300
C 4.23593400 4.04659000 2.19820500
H 3.51455500 6.02128900 1.71218100
H 1.79481100 5.19799700 0.12242600
H 3.00742000 1.14973800 0.88409500
H 4.73312700 1.96402000 2.47760000
H 4.98959800 4.40350900 2.89465500
C 1.23083000 2.61691900 -0.56268700
O 0.30587600 1.84844300 0.11415000
O 1.81563000 1.78902100 -1.60418500
C 2.61389200 2.50423700 -2.53968700
H 3.50355500 2.93694700 -2.06484800
H 2.92839600 1.78945300 -3.30388400
H 2.03758300 3.30906000 -3.01964700
H 0.75366700 3.47348900 -1.07174200
H -0.47327500 1.61162800 -0.48851600

$\mathbf{2H}^{s}$-IN5$_{MB}$

Ru 0.63092000 -0.71605200 0.25117600
P -0.97369100 -1.68473500 -0.99652700
P 2.34396600 -0.51365100 -1.22912000
N -0.92354700 -0.82616000 1.84413400
H -1.08961200 0.16850200 2.06607000
H -0.52239900 -1.26767300 2.66937100
N 2.13718100 0.32342200 1.58340200
H 2.06594300 0.23425000 2.59322800
C -2.39072800 -1.92703700 0.19658600
C -3.61491100 -2.53271000 -0.12402800
H -3.79617600 -2.87958900 -1.13793800
C -4.60786300 -2.70520300 0.84187300

H	-5.54871000	-3.17946900	0.57725800
C	-4.38349700	-2.26816400	2.15151900
H	-5.14854400	-2.40000600	2.91136700
C	-3.17363000	-1.65537500	2.48229900
H	-2.99762700	-1.30428200	3.49648100
C	-2.18065500	-1.48223700	1.50969800
C	3.79836800	0.00411700	-0.18118800
C	5.12862900	0.05356500	-0.61812800
H	5.36558400	-0.17732200	-1.65358900
C	6.16665500	0.36801000	0.26282700
H	7.19429500	0.38627700	-0.08843800
C	5.87244400	0.64522600	1.60113500
H	6.67117600	0.88286000	2.29823700
C	4.54881000	0.63002300	2.04489100
H	4.31803300	0.87024000	3.08034400
C	3.50877300	0.31799800	1.15944600
H	1.10085500	-2.21111300	0.56232700
C	3.02251100	-1.94720000	-2.20722900
H	2.25436300	-2.27721000	-2.91303500
H	3.92257400	-1.67983900	-2.76923900
H	3.24381500	-2.77006500	-1.52376800
C	2.26465200	0.82450100	-2.52503900
H	3.23942200	1.01256100	-2.98614800
H	1.54885000	0.52744100	-3.29775900
H	1.89117800	1.72845700	-2.03817400
C	-0.75733700	-3.40583300	-1.69182500
H	0.00108500	-3.37568900	-2.47998900
H	-0.40185500	-4.06361600	-0.89595800
H	-1.68748100	-3.80022600	-2.11340400
C	-1.77394100	-0.83762500	-2.45486900
H	-1.03539300	-0.77812000	-3.26066100
H	-2.65196200	-1.38050800	-2.82014400
H	-2.05932700	0.17648500	-2.17011400
C	-2.64554100	3.54311600	-2.40496300
C	-1.61588800	3.40618600	-1.47157700
C	-1.78222000	2.62114800	-0.32091200
C	-3.01281400	1.98485300	-0.11941600
C	-4.05023600	2.12073400	-1.04976000
C	-3.87094500	2.89894200	-2.19622600
H	-2.49623600	4.15506000	-3.29092200
H	-0.66466800	3.90742000	-1.63570900
H	-3.16115000	1.38122100	0.77016400
H	-4.99899700	1.61955500	-0.87495200
H	-4.67702400	3.00784600	-2.91683700
C	-0.58944500	2.48314400	0.63804100
O	0.35804100	1.64412400	0.20413900
O	-1.07534200	2.04767400	1.97907000
C	-1.67595700	3.06925300	2.75838400
H	-2.60486100	3.44453200	2.30620700
H	-1.90831400	2.64292600	3.73913200
H	-0.98791400	3.91723100	2.89588700
H	-0.18354200	3.50447500	0.81685600
H	1.62158400	1.18765500	1.26915800

2H^{s}-IN6$_{MB}$

Ru	-0.18778400	-1.14548300	0.07586900
P	-1.95181300	-1.12027300	-1.36803500
P	1.62236100	-1.75914600	-1.17773100
N	-1.72591600	-0.65946000	1.51676000
H	-0.90461200	0.53092600	2.47504100
H	-1.72221300	-1.25354100	2.34585300
N	1.32850500	-1.14506600	1.72531400
H	1.05924000	-1.98687600	2.23259500
C	-3.36357200	-0.59622500	-0.29654200

C	-4.67703500	-0.38690300	-0.74064200
H	-4.90230700	-0.44169000	-1.80407500
C	-5.71249100	-0.13494000	0.15975300
H	-6.72676100	0.02446000	-0.19415000
C	-5.42059300	-0.11117300	1.53282000
H	-6.21735600	0.06526300	2.25180300
C	-4.12001400	-0.30000800	1.98765200
H	-3.90693800	-0.26135700	3.05431900
C	-3.04796400	-0.52414700	1.08610700
C	3.05553000	-1.58254900	0.02201500
C	4.41013800	-1.71373500	-0.32082000
H	4.68510800	-1.98706500	-1.33613900
C	5.41974300	-1.48205300	0.61557500
H	6.46262500	-1.57962600	0.32718400
C	5.08199100	-1.11402600	1.92167500
H	5.85975000	-0.92205200	2.65535800
C	3.73924300	-0.99907100	2.28583500
H	3.46834400	-0.72549900	3.30276500
C	2.73008900	-1.23894400	1.34479000
H	-0.46621700	-2.68372700	0.36323700
C	1.84934800	-3.50100500	-1.81738700
H	1.11975300	-3.67649500	-2.61397900
H	2.85688700	-3.67345200	-2.20989400
H	1.64402800	-4.20290400	-1.00596700
C	2.24927900	-0.78845700	-2.65279500
H	2.29418900	0.26986900	-2.38516700
H	3.23391800	-1.12314800	-2.99437700
H	1.53443400	-0.90803500	-3.47311600
C	-2.56873800	-2.67745300	-2.19182100
H	-1.83141700	-3.01327000	-2.92803800
H	-2.67980200	-3.45219000	-1.43001700
H	-3.52895500	-2.51605300	-2.69194500
C	-1.99734500	0.06844400	-2.80852800
H	-1.26798400	-0.24890200	-3.56040100
H	-2.98931200	0.10825100	-3.26983900
H	-1.72220100	1.06312800	-2.45120600
C	1.15263600	5.85313600	-0.09890200
C	0.90106300	4.59577700	0.45214500
C	0.68729200	3.48611400	-0.38248100
C	0.72738200	3.64856700	-1.77928800
C	0.97853600	4.90360000	-2.32705100
C	1.19143500	6.00693100	-1.48823300
H	1.31625500	6.70905500	0.54926100
H	0.86682100	4.46577800	1.53111600
H	0.55792200	2.78326100	-2.41236500
H	1.00816200	5.02975500	-3.40553000
H	1.38591300	6.98484000	-1.91973400
C	0.42109100	2.17428600	0.23414400
O	0.21833500	1.14870700	-0.41947600
O	-0.23435900	0.99380900	3.07502200
C	-0.84332300	1.34477100	4.30673300
H	-1.27597700	0.47108300	4.81727100
H	-0.07395900	1.77262300	4.95745000
H	-1.63763100	2.09293200	4.17149700
H	0.40118500	2.13851900	1.33567800
H	1.15113800	-0.35539800	2.35634000

2H^{s}-IN6$_{MB}$′

Ru	0.03210100	-0.21502100	-0.13350000
P	-1.75478400	1.17149400	0.09937200
P	1.85234300	1.17429900	-0.11120200
N	-1.55316900	-1.78832300	-0.24282600
H	-1.35653300	-2.54384400	0.41313100
H	-1.40201700	-2.16788700	-1.17723200

N 1.50323200 -1.64859500 0.10509200

H 1.34048900 -2.65214200 0.08635200

C -3.22372300 0.00525600 0.05269400

C -4.56470900 0.40158000 0.17695900

H -4.80596400 1.45448500 0.29586300

C -5.60096200 -0.53355500 0.15537400

H -6.63185600 -0.20559900 0.25411400

C -5.30679800 -1.89286600 0.00963100

H -6.10565900 -2.62849600 -0.00604500

C -3.97926700 -2.30449000 -0.11979600

H -3.74376900 -3.35975000 -0.23909600

C -2.94575000 -1.36077200 -0.09983100

C 3.26486500 -0.00195900 0.02084600

C 4.62084400 0.33431000 0.04323400

H 4.91973000 1.37858800 -0.03096000

C 5.60845800 -0.64922700 0.15612000

H 6.65987700 -0.37916900 0.17067800

C 5.21029200 -1.99190700 0.24747100

H 5.96314000 -2.77218900 0.33363000

C 3.86509100 -2.34666300 0.22896300

H 3.57815800 -3.39421000 0.30224400

C 2.84900800 -1.36153100 0.11772500

H -0.18512600 0.13875100 -1.65120100

C 2.24523800 2.23911100 -1.59025100

H 1.49049600 3.02592600 -1.68220100

H 3.23475900 2.69825200 -1.50071100

H 2.21279900 1.61585000 -2.48657300

C 2.14404500 2.38776000 1.28076500

H 3.14543900 2.82613400 1.22150800

H 1.40029200 3.18940200 1.23138600

H 2.03805900 1.86731000 2.23594200

C -2.26034100 2.48200700 -1.13912800

H -1.50958000 3.27846500 -1.12200400

H -2.26852100 2.03996500 -2.13823200

H -3.24039200 2.91718100 -0.91924400

C -2.07629200 2.11127600 1.69028800

H -1.31434200 2.89053100 1.78972900

H -3.06650900 2.57833200 1.71135300

H -1.97919500 1.42659500 2.53675700

2H^{s}-IN7$_{MB}$

Ru -0.02297200 -0.11757100 -0.06055000

P 1.77532500 -1.49836400 0.27867500

P -1.88485100 -1.45628600 0.06622800

N 1.54841800 1.31594300 -0.49120300

H 1.38696400 1.93531100 -1.28351500

N -1.54324600 1.44940500 -0.53447900

H -1.35110800 1.69503800 -1.50446700

C 3.21752500 -0.40060100 -0.04297700

C 4.56074900 -0.79697300 0.03684300

H 4.80794700 -1.79580600 0.39172300

C 5.59009600 0.05652500 -0.35759000

H 6.62679900 -0.26017800 -0.29567000

C 5.25607400 1.32709200 -0.85687600

H 6.04344900 2.00073700 -1.18716700

C 3.93243600 1.74220600 -0.92744600

H 3.69375800 2.73658100 -1.30009700

C 2.87017100 0.90123800 -0.49765600

C -3.29613400 -0.24580300 -0.15360300

C -4.65563100 -0.58115500 -0.05421500

H -4.94577000 -1.61176300 0.13250100

C -5.64900200 0.39134400 -0.17958200

H -6.69583500 0.11420000 -0.09391700

C -5.28999400 1.72349100 -0.40886600

H	-6.05541700	2.48865100	-0.50081700
C	-3.94315100	2.07123900	-0.52162500
H	-3.65693500	3.10410100	-0.70418300
C	-2.94938000	1.09207200	-0.39963700
H	0.03420400	-0.51847100	-1.60846100
C	-2.23086100	-2.73037600	-1.25336700
H	-1.50755400	-3.54525700	-1.15157000
H	-3.24380000	-3.13862100	-1.18010900
H	-2.08845300	-2.26701900	-2.23195500
C	-2.41822600	-2.40294900	1.58973500
H	-3.40364900	-2.86589800	1.47632600
H	-1.68188500	-3.18908700	1.78396100
H	-2.43375400	-1.72517000	2.44723500
C	2.06625200	-2.98746700	-0.80598000
H	1.29370300	-3.73691700	-0.60643200
H	1.99443000	-2.67702100	-1.85056300
H	3.05034100	-3.43016400	-0.62322200
C	2.09944800	-2.23635600	1.96447700
H	1.32404600	-2.97359500	2.19526400
H	3.07828300	-2.72455000	2.01009600
H	2.06215000	-1.44165700	2.71392500
O	0.21761600	3.23156200	0.97932600
C	0.85206100	4.42922900	1.39683900
H	0.07984800	5.09897100	1.78761600
H	1.58527300	4.24593900	2.19501700
H	1.36239400	4.93815900	0.56560100
H	0.88305300	2.59780900	0.58756900
H	0.09686800	0.76779400	1.64665800
H	-0.23348400	0.07021800	1.82409300
H	-1.30396000	2.27402900	0.02951100

2H^{s}-IN7$_{MB}$′

Ru	0.02695700	-0.21179300	0.17936300
P	-1.77817400	1.18188300	-0.06217500
P	1.86083800	1.17157200	0.08013600
N	-1.55608600	-1.80048900	-0.00287100
H	-1.43736500	-2.53673400	0.69065900
H	-1.29121200	-2.19910500	-0.90294300
N	1.56881600	-1.69548500	0.15848500
H	1.40226100	-2.69596700	0.13454600
C	-3.23645000	0.00376700	-0.09957800
C	-4.58100600	0.40345200	-0.15402700
H	-4.82715400	1.46063200	-0.20686300
C	-5.61556300	-0.53332900	-0.12943300
H	-6.64964500	-0.20276800	-0.16476400
C	-5.31537500	-1.89698800	-0.05333500
H	-6.11308300	-2.63359300	-0.02936800
C	-3.98374000	-2.31277900	-0.01290000
H	-3.74393400	-3.37262300	0.03486400
C	-2.95043200	-1.36858400	-0.03778600
C	3.26278100	0.00244000	-0.05430400
C	4.60811700	0.36659700	-0.19358100
H	4.87766000	1.42115400	-0.23094000
C	5.61557300	-0.59323800	-0.29151000
H	6.65484200	-0.30036100	-0.40250500
C	5.24773300	-1.95230400	-0.24769300
H	6.01642800	-2.71839600	-0.32629000
C	3.92445500	-2.33988300	-0.10695800
H	3.66780900	-3.39750100	-0.07484300
C	2.87328400	-1.37569500	0.00027400
H	0.09856200	-0.20163100	-1.41561300
C	2.06376300	2.36497500	-1.33751000
H	1.32279800	3.16703900	-1.25872400
H	3.06521000	2.80639900	-1.33771100

H 1.90589400 1.82553900 -2.27371000
C 2.27997100 2.28390800 1.52347600
H 3.25830400 2.75685100 1.38970900
H 1.51683700 3.06208500 1.62645500
H 2.29835200 1.68422500 2.43697900
C -2.03685800 2.17298000 -1.62414600
H -1.30234900 2.98356600 -1.65004900
H -1.86232600 1.52416800 -2.48513400
H -3.04250500 2.60126800 -1.67958900
C -2.33181400 2.43905400 1.21015300
H -1.57219400 3.22502800 1.26495800
H -3.29464900 2.89736600 0.96268700
H -2.39968100 1.95722000 2.18903900
H -0.21180200 0.03218800 2.06766700
H 0.20110900 -0.64339500 2.04396200

2H^{s}-IN8$_{MB}$

Ru -0.00000800 -0.06936500 -0.25724500
P 1.75914400 -1.39070900 0.21015400
P -1.75915400 -1.39067600 0.21023200
N 1.60957600 1.39990500 -0.81602700
H 1.41175500 2.26276400 -0.30016900
H 1.47566600 1.58957200 -1.80721500
N -1.60955000 1.39997900 -0.81583800
H -1.47568600 1.58990400 -1.80698400
C 3.24907700 -0.31190800 -0.13183200
C 4.58292800 -0.70364500 0.06043100
H 4.80476400 -1.70531100 0.41932000
C 5.63705800 0.17437300 -0.19774700
H 6.66328500 -0.14629900 -0.04175700
C 5.36585900 1.46831200 -0.65422200
H 6.17879600 2.16051100 -0.85421800
C 4.04437900 1.87084000 -0.85384900
H 3.82578600 2.87519600 -1.20926100
C 2.99063800 0.98498700 -0.59695800
C -3.24908200 -0.31187700 -0.13175500
C -4.58294100 -0.70363900 0.06041700
H -4.80478200 -1.70531200 0.41927900
C -5.63706800 0.17435800 -0.19784500
H -6.66330100 -0.14633600 -0.04194200
C -5.36585700 1.46829800 -0.65430900
H -6.17879300 2.16047500 -0.85438700
C -4.04437000 1.87084600 -0.85385300
H -3.82577300 2.87520400 -1.20925900
C -2.99063200 0.98501400 -0.59687700
H -0.00003000 -0.60147500 -1.86043300
C -2.15041300 -2.93192800 -0.77327100
H -1.37799600 -3.67935800 -0.56634600
H -3.12909200 -3.35500200 -0.52365200
H -2.10968400 -2.68356900 -1.83588500
C -2.11481700 -2.00475300 1.94039700
H -3.09716200 -2.48038300 2.02749300
H -1.34464200 -2.73261900 2.21444600
H -2.04591700 -1.15800800 2.62658400
C 2.15037100 -2.93194700 -0.77338100
H 1.37796300 -3.67937800 -0.56642400
H 2.10959600 -2.68358800 -1.83599200
H 3.12906000 -3.35502400 -0.52380600
C 2.11485200 -2.00481800 1.94030100
H 1.34467100 -2.73267400 2.21436400
H 3.09719100 -2.48046200 2.02736700
H 2.04597900 -1.15807800 2.62649700
O -0.00008600 3.02819100 1.05397100
C 0.00011700 3.76365100 2.27131300

H	-0.89040400	4.40286300	2.32924100
H	0.00148600	3.09279600	3.13962600
H	0.88948400	4.40458100	2.32775800
H	-1.41176400	2.26274500	-0.29980100
H	-0.00003700	2.03525200	1.24827400
H	-0.00000400	0.58213300	1.37978900

2H-TS1$_{DMO}$

Ru	0.01900200	-0.40988600	-1.04203000
P	-1.94827100	0.23969800	-0.10997900
P	1.54221200	0.95531200	-0.04157100
N	-1.26970300	-1.79163000	-2.18246700
H	-0.93467700	-2.73101500	-1.89426000
H	-1.06901000	-1.69409000	-3.17609400
N	1.79298400	-1.12279700	-2.18188000
H	1.60194700	-0.99573300	-3.17383300
H	1.85683600	-2.12930200	-2.00363000
C	-3.21462600	-0.78473400	-1.04264800
C	-4.60399800	-0.70929700	-0.85804000
H	-5.01215100	-0.01360200	-0.13250300
C	-5.47287500	-1.50756400	-1.60200200
H	-6.54548000	-1.43185600	-1.44784700
C	-4.95798500	-2.40225200	-2.54646400
H	-5.62688000	-3.02890400	-3.12943500
C	-3.57893100	-2.49219800	-2.73651200
H	-3.16880800	-3.19266300	-3.46003400
C	-2.70936400	-1.68717500	-1.98849700
C	3.16290000	0.42975200	-0.83266300
C	4.41941900	0.96965300	-0.51445400
H	4.50203700	1.69611700	0.28726500
C	5.56378400	0.59471400	-1.21785900
H	6.52627100	1.02574500	-0.95757900
C	5.46497800	-0.32665500	-2.26637800
H	6.34850300	-0.61449700	-2.82904500
C	4.22532400	-0.88029900	-2.58583700
H	4.14068300	-1.60606600	-3.39166000
C	3.08150700	-0.51235200	-1.86544100
C	-2.39025400	-0.08070500	1.68288100
C	-3.54558600	0.43017100	2.30128200
H	-4.21376600	1.08997800	1.75645100
C	-3.83893400	0.11629600	3.62989100
H	-4.73520000	0.52015200	4.09345300
C	-2.97919500	-0.71043500	4.36223700
H	-3.20680200	-0.95210100	5.39709400
C	-1.82549300	-1.21535000	3.75930800
H	-1.14552200	-1.85163100	4.31850600
C	-1.53146100	-0.90034400	2.42846700
H	-0.63490100	-1.29502700	1.96020800
C	-2.34360700	2.96578400	0.62219900
H	-1.87770300	2.69456200	1.56475500
C	-2.71203700	4.29579100	0.40209400
H	-2.52230600	5.04002600	1.17070900
C	-3.31833800	4.66664800	-0.80062700
H	-3.60822100	5.69963200	-0.97187100
C	-3.54507600	3.69880700	-1.78388600
H	-4.01325800	3.97609400	-2.72465700
C	-3.17139300	2.37056200	-1.56384400
H	-3.34367700	1.63382600	-2.34231000
C	-2.57423700	1.98382700	-0.35399400
C	1.88596100	0.85174600	1.79031800
C	2.66355400	-0.19181300	2.31382500
H	3.13794300	-0.90518700	1.64941000
C	2.82005700	-0.34619200	3.69343800
H	3.41874200	-1.16888700	4.07342600

C	2.19621900	0.54027300	4.57525800
H	2.31652200	0.42018400	5.64835800
C	1.40621700	1.57522300	4.06692400
H	0.90598100	2.26368400	4.74267500
C	1.24952400	1.72619300	2.68698100
H	0.63114100	2.53550100	2.31057700
C	1.61105100	2.80862200	-0.34974000
C	2.42139100	3.68125300	0.39773600
H	2.99117200	3.31046700	1.24368700
C	2.49517100	5.03863800	0.07615500
H	3.12622800	5.69752100	0.66683700
C	1.75894600	5.54676000	-0.99899300
H	1.81524000	6.60302400	-1.24831900
C	0.94685300	4.68910800	-1.74416500
H	0.36231800	5.07508600	-2.57487700
C	0.87221000	3.33086400	-1.42040300
H	0.23879600	2.65957700	-1.99317000
H	-0.26151500	0.69658000	-2.22912200
H	0.35025700	-1.82779100	0.04714300
C	0.36593600	-3.59997800	0.06378200
O	0.05386800	-3.96606500	-1.08603600
O	-0.42009000	-3.82719400	1.14907600
C	1.82630700	-3.54141600	0.52561000
O	2.20741700	-3.54374400	1.67478400
O	2.66257500	-3.54901400	-0.53999500
C	-1.79068400	-4.13295000	0.87816900
H	-2.22081900	-4.41871000	1.83844500
H	-2.31090400	-3.25060300	0.49208700
H	-1.87167200	-4.95192400	0.15851400
C	4.06454100	-3.62783800	-0.21649600
H	4.57805700	-3.73174000	-1.17154500
H	4.39557800	-2.72077000	0.29463400
H	4.25402800	-4.49353900	0.42225000

2H-TS1$_{MB}$

Ru	-0.16307200	-0.40798000	-0.86971500
P	-0.88584600	1.57234600	-0.01064600
P	2.00499400	-0.55477200	-0.14403200
N	-2.11159800	-0.33767900	-1.93487900
H	-2.66163100	-1.14490800	-1.63315100
H	-1.89961700	-0.47127100	-2.92197500
N	0.36878400	-2.34446000	-1.82090100
H	-0.09855800	-2.49484200	-2.71307400
C	-2.49995200	1.87598000	-0.91762500
C	-3.29953400	3.02159300	-0.77867400
H	-2.98884600	3.81467700	-0.10670800
C	-4.48318700	3.16473800	-1.50186200
H	-5.08550800	4.06102100	-1.38405200
C	-4.88935300	2.15346400	-2.37957600
H	-5.80959500	2.25642200	-2.94736700
C	-4.10951800	1.00638100	-2.52157500
H	-4.42336200	0.20900100	-3.19068900
C	-2.92217500	0.86931500	-1.79190500
C	2.71042100	-1.90484000	-1.22806000
C	4.07703100	-2.17365600	-1.39370400
H	4.80839500	-1.59724000	-0.83475700
C	4.51224900	-3.14830100	-2.29375400
H	5.57469800	-3.33684900	-2.41806700
C	3.57576400	-3.86492200	-3.04516900
H	3.90432500	-4.62128700	-3.75231500
C	2.21302900	-3.60656100	-2.88952600
H	1.48080600	-4.16629200	-3.46683500
C	1.77996700	-2.62799500	-1.98677400
H	0.43075500	0.43194600	-2.15406500

C	-5.25001000	-1.70236900	0.45024700
C	-3.94299800	-1.88915700	0.90306800
C	-3.06699600	-2.73631800	0.20743600
C	-3.52505200	-3.39719900	-0.94138300
C	-4.83611400	-3.20908500	-1.39478500
C	-5.70234900	-2.36072100	-0.69982600
H	-5.91828900	-1.04229800	0.99635100
H	-3.59714300	-1.38262900	1.79634800
H	-2.84907800	-4.07916400	-1.44802200
H	-5.18262100	-3.73795700	-2.27895800
H	-6.72222000	-2.21655000	-1.04580800
C	-1.65510500	-3.01905900	0.68088300
O	-0.93344800	-3.84466000	0.06757900
O	-1.60865100	-2.92710100	2.05731000
C	-0.39982100	-3.39539000	2.65915400
H	0.44341900	-2.77257000	2.34951200
H	-0.55324100	-3.31111600	3.73633400
H	-0.20482500	-4.43418700	2.37952200
H	-0.05472500	-3.02578200	-1.14768900
H	-0.98214500	-1.51868800	0.34055200
C	0.04013600	3.16753000	-0.31954800
C	0.95705500	3.65920400	0.62267500
C	-0.08486100	3.84141600	-1.54446700
C	1.71209800	4.80488400	0.35829700
H	1.08497600	3.15266500	1.57461800
C	0.66541100	4.98993900	-1.80902900
H	-0.77131300	3.47008200	-2.29886700
C	1.56436600	5.47843600	-0.85653500
H	2.41710200	5.16614000	1.10180100
H	0.54628100	5.50168800	-2.76040100
H	2.14822700	6.37141800	-1.06171400
C	-1.41618700	1.78027000	1.78058300
C	-1.72260900	3.02202700	2.36447100
C	-1.56167300	0.61910100	2.55282100
C	-2.17489100	3.09719000	3.68400000
H	-1.59818200	3.93975700	1.79829400
C	-2.01712500	0.69343600	3.87346500
H	-1.31874700	-0.34272800	2.10930200
C	-2.32638100	1.93117000	4.44229800
H	-2.40657900	4.06574100	4.11921000
H	-2.12568700	-0.21888700	4.45385400
H	-2.67815500	1.98999700	5.46883500
C	3.26514500	0.80520800	-0.43925100
C	4.36235000	1.02742800	0.40820900
C	3.14025400	1.58747300	-1.59817600
C	5.31517800	2.00470400	0.09999400
H	4.48390100	0.44118200	1.31272100
C	4.09634500	2.55582100	-1.90997900
H	2.28176800	1.43448200	-2.24520800
C	5.18780200	2.76842400	-1.06230400
H	6.15707100	2.16262200	0.76905800
H	3.98172800	3.15154400	-2.81136900
H	5.92907600	3.52573900	-1.30318700
C	2.46670100	-1.07827400	1.59418200
C	3.17658000	-2.24961600	1.89686300
C	2.04268700	-0.26048000	2.65589500
C	3.46306900	-2.58937200	3.22429600
H	3.50285200	-2.91090400	1.10161100
C	2.33526100	-0.59258200	3.97961800
H	1.46794000	0.63773800	2.45117600
C	3.04879900	-1.76072900	4.26919600
H	4.01050900	-3.50380400	3.43674300
H	1.99691900	0.05568400	4.78327900
H	3.27289800	-2.02407700	5.29918700

3H-TS1$_{DMO}$

Ru	0.00139400	-0.42100400	-1.11416400
P	-1.70775900	0.80115100	-0.13410100
P	1.80823100	0.43495900	0.01886400
N	1.37945200	-1.49443100	-2.46678800
C	-3.47678500	0.01573700	-2.22125800
C	-4.42800900	0.22807600	-3.22650800
C	-4.94763100	1.50062900	-3.47974900
C	-4.51403500	2.57752000	-2.70893400
C	-3.56373300	2.37773100	-1.70111500
C	-3.02738900	1.10888300	-1.44420500
C	2.75788500	-0.98783800	-2.68227200
C	3.65429700	-1.17077300	-1.47702400
C	4.84888900	-1.88502300	-1.65378600
C	5.74353200	-2.08658700	-0.60160400
C	5.43853000	-1.57080500	0.65761600
C	4.25457700	-0.85336300	0.84612300
C	3.34955300	-0.63480400	-0.20422000
C	2.97898800	-1.39000300	-1.97391400
N	1.53463700	-1.54679700	-2.28151500
H	5.07642200	-2.29359100	-2.63571400
H	6.66150700	-2.64389300	-0.76498800
H	6.11670700	-1.71931700	1.49346600
H	4.03879200	-0.45694000	1.83181800
H	3.24360900	3.22814400	-1.10998400
H	4.91177300	3.57379000	-2.88222800
H	5.68382400	1.64317700	-4.26573400
H	4.77109800	-0.61973700	-3.81575600
H	3.10619200	-1.66311400	-0.92484200
H	3.57634900	-2.09219800	-2.56998700
H	3.22284600	-1.49807100	-3.53601300
H	2.66944000	0.07170100	-2.94246800
H	0.90522900	-1.44873900	-3.36600900
H	1.37444800	-2.48533100	-2.19034800
H	-0.00800000	0.77610900	-2.25152300
H	0.04096200	-1.90449700	-0.05345800
H	-1.26789000	-2.52417000	-2.12355800
H	1.39889400	-1.34353300	-3.27066500
C	2.64474700	2.05416400	-0.47268500
C	3.70470000	2.61249500	0.26296500
C	2.28277700	2.65922300	-1.68350900
C	4.36302700	3.75831800	-0.18767500
H	4.02746200	2.15240400	1.19143200
C	2.94981900	3.79952900	-2.14500300
H	1.47160400	2.22170900	-2.25996100
C	3.98738400	4.35703800	-1.39525600
H	5.17495300	4.17806000	0.40027400
H	2.65479100	4.25044400	-3.08916400
H	4.50435200	5.24511200	-1.74868700
C	1.66585400	0.53909700	1.87744600
C	1.50727700	-0.66249800	2.59343000
C	1.60177300	1.75239800	2.57652900
C	1.33511700	-0.64752800	3.97814000
H	1.50475600	-1.61292900	2.06702100
C	1.41540200	1.76577300	3.96400700
H	1.68554100	2.69531200	2.04733500
C	1.29313600	0.56812900	4.67031400
H	1.22183700	-1.58741700	4.51135200
H	1.36424400	2.71705800	4.48668100
H	1.15499400	0.57962100	5.74806000
C	-1.39518600	2.53115200	0.51252500
C	-1.75093900	2.93710300	1.80751100
C	-0.74649200	3.45513800	-0.32402800
C	-1.46916900	4.23363600	2.25177700

H	-2.24484900	2.24536900	2.48010200
C	-0.47146700	4.75124800	0.11531800
H	-0.45679500	3.15413800	-1.32471600
C	-0.83171100	5.14560800	1.40817100
H	-1.75013400	4.52585600	3.26010700
H	0.03191200	5.44777400	-0.54929000
H	-0.61405900	6.15253400	1.75389200
C	-2.78051900	0.10180700	1.24153500
C	-4.13028900	0.45994500	1.40857500
C	-2.20243500	-0.79305200	2.15104600
C	-4.87863200	-0.06229800	2.46616000
H	-4.60122400	1.14883200	0.71417200
C	-2.94810700	-1.31034000	3.21613800
H	-1.17094000	-1.09859600	2.01507300
C	-4.28671800	-0.94586200	3.37661900
H	-5.92149800	0.22205700	2.57998500
H	-2.48144000	-2.01161700	3.90110500
H	-4.86907500	-1.35186600	4.19952400
C	-1.08371700	-3.80791000	0.62977200
O	-1.17734100	-3.88016800	1.83567500
O	-2.13156400	-3.91900100	-0.21299200
C	0.23043000	-3.66092100	-0.14899800
O	0.32120100	-3.93505600	-1.36067600
O	1.25757200	-3.86191000	0.72782800
C	-3.40426600	-4.15610800	0.42085000
H	-4.12073700	-4.25373000	-0.39500000
H	-3.36592100	-5.07693300	1.00820600
H	-3.66943900	-3.32290600	1.07566200
C	2.49651800	-4.31304600	0.16982900
H	3.09348500	-4.63942300	1.02255800
H	2.32782500	-5.14471100	-0.51999900
H	3.01448800	-3.50544100	-0.35046800

3H-TS1$_{MB}$

Ru	0.01447600	-0.38601000	-1.10280100
P	2.02425400	-0.13119600	0.00392000
P	-1.18470600	1.38150500	-0.17178500
N	-1.78581200	-0.93600100	-2.28783000
C	3.18268100	-2.37931700	-1.34884000
C	4.02360800	-3.49761300	-1.44675900
C	4.81725900	-3.91335600	-0.37690400
C	4.77703300	-3.19599100	0.81826100
C	3.93914400	-2.08425400	0.93137900
C	3.12541800	-1.66179100	-0.13172300
C	-3.11943100	-0.42005300	-1.87956300
C	-3.23582200	1.06464300	-2.13095400
C	-4.16103900	1.51539500	-3.08169400
C	-4.31990200	2.87650100	-3.35407200
C	-3.54217700	3.80385800	-2.66267400
C	-2.61551600	3.36562900	-1.71060400
C	-2.44356700	2.00240700	-1.43052800
C	2.40726700	-1.97010800	-2.58181500
N	0.94213300	-1.92670400	-2.36828900
H	-4.76894800	0.78595200	-3.61238500
H	-5.04323000	3.20392100	-4.09549900
H	-3.65288600	4.86757800	-2.85483100
H	-2.02536800	4.10322900	-1.17901000
H	3.92176900	-1.54281600	1.87002700
H	5.39188100	-3.49528600	1.66268300
H	5.45713300	-4.78520300	-0.47871200
H	4.05358300	-4.05117900	-2.38220800
H	2.71719600	-0.97380700	-2.91426800
H	2.65182400	-2.67433700	-3.38779000
H	-3.91633600	-0.94013700	-2.42426100
H	-3.24152700	-0.65965500	-0.82039300

H	-1.79179800	-1.95702500	-2.22116500
H	-1.63080900	-0.68936600	-3.26433400
H	-0.56991900	-1.81971500	-0.06753600
H	0.42817300	0.70762600	-2.25824800
H	0.51694900	-1.78394900	-3.28208300
H	0.57837600	-2.82991700	-2.01700200
C	-2.29278600	1.20082200	1.33755300
C	-3.55471800	1.80722700	1.44901000
C	-1.80605700	0.46257400	2.42538500
C	-4.30663100	1.68042600	2.62138000
H	-3.95736800	2.38597000	0.62419000
C	-2.54637200	0.35207900	3.60531600
H	-0.84291700	-0.02856300	2.34621200
C	-3.80165000	0.95852400	3.70657500
H	-5.28350900	2.15235800	2.68644000
H	-2.14297600	-0.21364200	4.44095500
H	-4.38185500	0.86799600	4.62100900
C	-0.30049600	2.97979300	0.25953800
C	0.59231800	3.52007600	-0.68016700
C	-0.49013200	3.66253700	1.47054900
C	1.27290800	4.71134100	-0.42186500
H	0.74973500	2.99886400	-1.61768100
C	0.19768100	4.85224700	1.73478400
H	-1.17225000	3.27203000	2.21678900
C	1.07985500	5.38182500	0.79038100
H	1.96003900	5.10884600	-1.16358500
H	0.03868600	5.36295500	2.68089900
H	1.61310800	6.30602100	0.99604900
C	1.97132700	0.14779200	1.85247800
C	2.23360800	1.39633800	2.43491200
C	1.53794900	-0.90195000	2.68391900
C	2.10119200	1.58238000	3.81594700
H	2.53674100	2.23515600	1.81747400
C	1.42412200	-0.72124000	4.06341200
H	1.27554700	-1.86214600	2.25030200
C	1.71024000	0.52298000	4.63641300
H	2.30580300	2.55950500	4.24479500
H	1.10239000	-1.55070800	4.68765000
H	1.61821500	0.66599300	5.70954000
C	3.34270100	1.09055900	-0.58198800
C	4.51903300	1.33652400	0.14732300
C	3.21036700	1.67566500	-1.84787100
C	5.52090300	2.16212300	-0.36646200
H	4.66336900	0.88185800	1.12212600
C	4.21830700	2.49261300	-2.37190800
H	2.30446700	1.48248100	-2.41636600
C	5.37448300	2.74353800	-1.63072000
H	6.41906800	2.34449400	0.21760400
H	4.09545100	2.93293300	-3.35824300
H	6.15720900	3.38102700	-2.03290600
C	-4.35981500	-2.57325900	1.62102300
C	-2.99146600	-2.68338600	1.37321100
C	-2.53283700	-3.23765400	0.17215000
C	-3.46260700	-3.70534100	-0.76600600
C	-4.83499700	-3.60067200	-0.51588100
C	-5.28737100	-3.02943500	0.67615200
H	-4.70323700	-2.12555100	2.54914000
H	-2.27222300	-2.33036300	2.10179800
H	-3.09504400	-4.16934500	-1.67627300
H	-5.54753900	-3.97314600	-1.24745100
H	-6.35276900	-2.94315500	0.87200800
C	-1.05924000	-3.43019200	-0.12440200
O	-0.70054900	-3.84933500	-1.25570600
O	-0.42636300	-3.87519600	1.02571800

C	0.83183900	-4.52218000	0.83524200
H	1.58808400	-3.81694800	0.48057100
H	1.11642300	-4.90654500	1.81677300
H	0.74252100	-5.34301800	0.11780900

Potential reactive conformer **i** (**2H**-MB)

Ru	0.04161500	-0.36630500	-1.12071300
P	-0.76287000	1.47717500	-0.18376600
P	2.02638000	-0.51391700	-0.15104500
N	-1.92291500	-0.43782900	-2.11460200
H	-2.39327400	-1.28837800	-1.80526700
H	-1.77709900	-0.54708300	-3.11681200
N	0.64038400	-2.23911400	-2.12128100
H	0.73177600	-2.07195000	-3.12204800
H	-0.13773500	-2.8930580	-1.99672200
C	-2.41767700	1.69649600	-0.98254800
C	-3.28197500	2.77651400	-0.75699500
H	-2.99574500	3.55583900	-0.05905500
C	-4.49903100	2.86828000	-1.42946800
H	-5.15696700	3.71280700	-1.24720300
C	-4.86523200	1.87687200	-2.34395000
H	-5.81018000	1.94406300	-2.87470400
C	-4.01725800	0.79473300	-2.57291800
H	-4.29695500	0.01294600	-3.27354900
C	-2.80111100	0.70243600	-1.89161300
C	2.65152500	-2.17395400	-0.68751300
C	3.80736700	-2.79611700	-0.19599500
H	4.41451600	-2.29023600	0.54667900
C	4.17314300	-4.06903600	-0.62949200
H	5.06747200	-4.54064300	-0.23322400
C	3.38279300	-4.73868200	-1.56768400
H	3.65932600	-5.73280400	-1.90584000
C	2.23133500	-4.13165400	-2.06679700
H	1.60438000	-4.64696400	-2.78962800
C	1.86619500	-2.85536400	-1.62891700
C	-1.14531800	1.61628600	1.62180200
C	-1.51266400	2.82531000	2.23487300
H	-1.53744400	3.74393300	1.65802000
C	-1.82447400	2.86405800	3.59382900
H	-2.10837900	3.80584200	4.05518300
C	-1.76476900	1.69705300	4.36139800
H	-2.00235000	1.73027500	5.42102400
C	-1.38708500	0.49427700	3.76280700
H	-1.31973600	-0.41408300	4.35414700
C	-1.08062900	0.45656300	2.40171700
H	-0.77822200	-0.46739900	1.92291000
C	1.05744400	3.55278500	0.39782000
H	1.20239300	3.04791500	1.34683900
C	1.84368900	4.66098100	0.08036900
H	2.59588800	5.00663400	0.78379300
C	1.66979600	5.31715900	-1.13940300
H	2.28200400	6.17906300	-1.38854600
C	0.70387100	4.85792000	-2.03919600
H	0.56041200	5.36464800	-2.98953400
C	-0.07791100	3.74652400	-1.72330600
H	-0.81350900	3.38599100	-2.43562400
C	0.08841900	3.08229500	-0.50014600
C	2.13663000	-0.60755000	1.69013600
C	1.78735400	-1.78935700	2.35923800
H	1.56241500	-2.68403600	1.78808500
C	1.71175800	-1.82256600	3.75137600
H	1.44431100	-2.74780900	4.25444000
C	1.97764800	-0.67114300	4.49793000
H	1.91335300	-0.69584200	5.58183900

C	2.32389000	0.51056600	3.84161000
H	2.52707600	1.41273800	4.41149500
C	2.40466100	0.54090900	2.44841300
H	2.67622600	1.46456700	1.94954200
C	3.42384100	0.64564500	-0.52743900
C	4.65831900	0.59549100	0.14125600
H	4.82328700	-0.13327000	0.92767400
C	5.67297500	1.49842900	-0.17337300
H	6.62317400	1.44631900	0.35082200
C	5.46508300	2.47328000	-1.15402700
H	6.25408300	3.18080100	-1.39311100
C	4.23874400	2.53821300	-1.81578100
H	4.06171900	3.30260800	-2.56676100
C	3.22544300	1.62881100	-1.50465700
H	2.26509100	1.67591300	-2.00805100
H	0.55132000	0.50595100	-2.51766900
H	-0.54308900	-1.36483700	0.09265700
C	-4.38676300	-0.93699400	1.81004400
C	-3.38817900	-1.85324100	1.48981400
C	-3.39035900	-2.47860200	0.23613500
C	-4.41558800	-2.20420100	-0.67959800
C	-5.42532000	-1.30396800	-0.34579600
C	-5.40491800	-0.66078500	0.89392200
H	-4.35951000	-0.42642700	2.76731700
H	-2.58804400	-2.05980100	2.18835500
H	-4.41049600	-2.69765100	-1.64608100
H	-6.21784000	-1.09305500	-1.05614400
H	-6.17881300	0.05867000	1.14369100
C	-2.27306600	-3.36119300	-0.18719700
O	-1.98480900	-3.57020800	-1.36270300
O	-1.63567700	-3.92584500	0.84353400
C	-0.44170500	-4.66672200	0.52995900
H	0.34614200	-3.98046000	0.21574700
H	-0.15365700	-5.16633600	1.45470600
H	-0.63478200	-5.40034100	-0.25517400

Potential reactive conformer **ii** (**2H**-MB)

Ru	0.31900100	0.10834800	-0.91414200
P	-1.44984600	1.35767000	-0.36958900
P	-0.20345500	-1.87011400	-0.06073300
N	1.04056500	2.09068900	-1.64620500
H	1.71050500	1.99319700	-2.40625200
H	1.54902400	2.49551700	-0.85204600
N	2.21200600	-0.93447200	-1.43344500
H	2.99389700	-0.29569300	-1.29172100
C	-1.29791400	2.80686400	-1.50082600
C	-2.33273400	3.67442300	-1.86167700
H	-3.32158000	3.53537600	-1.43529300
C	-2.10734900	4.69916300	-2.78294100
H	-2.92060800	5.35830600	-3.07159100
C	-0.83756600	4.86637200	-3.33944700
H	-0.65842600	5.65759800	-4.06128600
C	0.20914500	4.01868600	-2.96905400
H	1.20201800	4.15010400	-3.39139900
C	-0.02084000	2.99186700	-2.05342900
C	1.43089700	-2.72420300	0.06675800
C	1.67833600	-3.89133400	0.80073800
H	0.87845600	-4.33625800	1.38428600
C	2.94388700	-4.47581200	0.79821100
H	3.12928400	-5.37237000	1.38240700
C	3.97119900	-3.90767900	0.03944200
H	4.95759300	-4.36171100	0.02783400
C	3.73192400	-2.75381800	-0.70631700
H	4.52357300	-2.30375700	-1.29648800

C 2.46949600 -2.15804100 -0.68229700
H 1.02541900 0.40289700 0.57008400
H 2.19225200 -1.15127800 -2.42889500
H -0.23904200 -0.13666100 -2.53473800
C 5.96195600 -0.29955600 0.11590800
C 4.94785300 0.28447000 0.87310600
C 4.24177500 1.38369000 0.36614300
C 4.55413100 1.88559800 -0.90629300
C 5.55761700 1.28882800 -1.66621200
C 6.26610500 0.19779800 -1.15354700
H 6.50616800 -1.15078500 0.51226300
H 4.69675600 -0.10883400 1.85049700
H 4.00984800 2.74423300 -1.28318100
H 5.79498800 1.67857100 -2.65129100
H 7.05322800 -0.26307500 -1.74272500
C 3.15045700 2.04158600 1.13580100
O 2.51916000 3.00740700 0.71986900
O 2.95747600 1.49334400 2.33696600
C 1.79133500 1.94711100 3.05761500
H 1.79164600 1.38697900 3.99226200
H 1.85549400 3.01852300 3.25396700
H 0.89977900 1.71713400 2.47567700
C -3.19143900 0.78677500 -0.55660100
C -4.26858800 1.37799900 0.12001800
C -3.43277700 -0.31295000 -1.39104600
C -5.56080200 0.87313900 -0.03498300
H -4.09705400 2.22226600 0.77939900
C -4.72320100 -0.82134800 -1.54190500
H -2.59707200 -0.77023200 -1.90602000
C -5.79012100 -0.22966000 -0.86180800
H -6.38764000 1.33696000 0.49545400
H -4.88972500 -1.68387400 -2.18114200
H -6.79511400 -0.62724200 -0.97111900
C -1.16464000 -3.19394600 -0.94641500
C -1.61481500 -4.36457300 -0.31523100
C -1.37321800 -3.04995100 -2.32337900
C -2.27504500 -5.35461700 -1.04320200
H -1.46100500 -4.50299000 0.74958200
C -2.03383000 -4.03993800 -3.05402500
H -1.01583600 -2.14630100 -2.81012200
C -2.49015800 -5.19348700 -2.41457000
H -2.62296900 -6.25191700 -0.53910100
H -2.19333200 -3.90701600 -4.12052500
H -3.00850300 -5.96343400 -2.97893100
C -1.51219400 2.17483500 1.28923800
C -1.15537500 3.51694300 1.47466400
C -1.82891800 1.39871600 2.41542500
C -1.10747900 4.06883600 2.75746200
H -0.90919300 4.13653800 0.61934600
C -1.78396500 1.94971100 3.69444600
H -2.10705900 0.36009900 2.29068800
C -1.41866100 3.28776400 3.87116900
H -0.82565600 5.11056400 2.88331700
H -2.02897400 1.33020200 4.55207200
H -1.37983300 3.71710000 4.86820500
C -0.90539700 -1.95724500 1.63584500
C -0.08698800 -1.68290600 2.74154000
C -2.28229400 -2.13224800 1.83728400
C -0.62907500 -1.61550000 4.02418300
H 0.97319200 -1.50484700 2.59296100
C -2.82539800 -2.05954000 3.12223600
H -2.93595700 -2.31485800 0.99124800
C -1.99994300 -1.80743800 4.21966200
H 0.01721000 -1.40414200 4.87130900

H -3.89397100 -2.19470500 3.26188300

H -2.42173600 -1.75268800 5.21895000

Potential reactive conformer **iii** (**2H**-MB)

Ru -0.28922100 0.15261100 -1.03712000

P 1.60410200 0.99278100 -0.24244100

P -0.25583000 -1.84883600 -0.09021200

N -0.54252900 2.19609400 -1.86369400

H -1.23484200 2.62900000 -1.25005600

H -0.93665000 2.18962500 -2.80256000

N -2.19637400 -0.55451300 -1.91194900

H -2.09219100 -0.57599500 -2.92502600

H -2.92493700 0.13129900 -1.71317800

C 1.75857600 2.62656500 -1.09595600

C 2.88850600 3.45420300 -1.05549100

H 3.75867100 3.14604200 -0.48554200

C 2.91701600 4.65692300 -1.75948100

H 3.80283000 5.28413200 -1.72702700

C 1.80773900 5.04724900 -2.51468100

H 1.82452900 5.98096500 -3.06865200

C 0.67322600 4.23699800 -2.55685800

H -0.19749900 4.53698900 -3.13425700

C 0.64907400 3.03152100 -1.85095500

C -1.87727200 -2.60086300 -0.56773400

C -2.36006300 -3.83144800 -0.10069700

H -1.76833600 -4.40767200 0.60240600

C -3.59691300 -4.31706800 -0.51928400

H -3.96047400 -5.26996000 -0.14625800

C -4.36640700 -3.57594500 -1.42081300

H -5.32958400 -3.95007400 -1.75480400

C -3.89982900 -2.34913400 -1.88921700

H -4.49515700 -1.76094700 -2.58101200

C -2.66429400 -1.85952700 -1.45729800

C 1.83299000 1.43331200 1.54349600

C 3.06346900 1.86307600 2.06775000

H 3.94207500 1.90409600 1.43270400

C 3.17591500 2.21757600 3.41157300

H 4.13381200 2.54804500 3.80363800

C 2.06077400 2.14432000 4.25213800

H 2.15054600 2.41798000 5.29972800

C 0.83627000 1.71382800 3.74110600

H -0.03440500 1.64687800 4.38573900

C 0.72394700 1.36024100 2.39481300

H 0.22288800 1.02374600 1.98806200

C 3.76691200 -0.73828500 0.30686500

H 3.33609900 -0.83306600 1.29746200

C 4.87946400 -1.50770200 -0.03623400

H 5.30071000 -2.19789900 0.68910000

C 5.44272700 -1.39620200 -1.30828000

H 6.30779000 -1.99540000 -1.57712200

C 4.88571200 -0.51117400 -2.23570800

H 5.31878300 -0.41823000 -3.22775800

C 3.77049000 0.25374900 -1.89397400

H 3.33270500 0.92515900 -2.62606800

C 3.20115300 0.15242600 -0.61672900

C -0.29109000 -1.92696300 1.75243700

C -1.47315800 -1.59651100 2.43199600

H -2.38298100 -1.41427300 1.86928300

C -1.48347500 -1.47766100 3.82070700

H -2.40723700 -1.22265800 4.33276800

C -0.30939100 -1.67912100 4.55285900

H -0.31643500 -1.57902700 5.63431100

C 0.87149700 -2.00542500 3.88555900

H 1.79042900 -2.15814900 4.44441500

C 0.88000500 -2.13008000 2.49456700

H 1.80435900 -2.38287100 1.98715000

C 0.97630800 -3.16635500 -0.50480400

C 1.07095600 -4.38110300 0.19431800

H 0.42161600 -4.57596700 1.04117100

C 2.01691900 -5.33765100 -0.17326300

H 2.07946400 -6.27303000 0.37588500

C 2.88619500 -5.09166600 -1.24020500

H 3.62618000 -5.83580400 -1.52119200

C 2.80482600 -3.88461500 -1.93516900

H 3.48687400 -3.67687000 -2.75426800

C 1.85624400 -2.92836600 -1.56771200

H 1.79473300 -1.98026900 -2.09112800

H 0.47395400 -0.26917100 -2.53050600

H -1.20427900 0.64806000 0.26821800

C -5.07550700 1.65122000 -1.60221800

C -4.10060000 2.38448800 -0.92286500

C -3.71988600 2.00824000 0.37204500

C -4.32467700 0.90150700 0.98239000

C -5.28860500 0.16655500 0.29879200

C -5.66801000 0.54162200 -0.99380800

H -5.37436500 1.94906700 -2.60264400

H -3.64590900 3.24933100 -1.39218800

H -4.01251700 0.62129200 1.98194400

H -5.73949900 -0.70273400 0.76678900

H -6.42287000 -0.02993700 -1.52488700

C -2.67851700 2.74306700 1.13964800

O -2.48247500 2.61719900 2.33252000

O -1.98001400 3.60678800 0.36410500

C -0.94259800 4.35350100 1.03594100

H -0.19952000 3.67497800 1.45677800

H -0.49472400 4.98499300 0.26992300

H -1.37471700 4.96539700 1.83085600

Potential reactive conformer **iv** (**2H**-MB)

Ru -0.36567500 -0.24852200 0.05932700

P 1.47038000 -1.52463300 0.08542500

P 0.52348700 1.77487600 -0.13000600

N -1.30830700 -2.27432500 0.30468200

H -2.27621100 -2.31838500 -0.02199000

H -1.33248100 -2.42487400 1.31250800

N -2.26325600 0.90279700 0.07882400

H -2.90041100 0.49786300 0.76243600

C 0.83962900 -3.15621600 -0.49048100

C 1.58856500 -4.14973600 -1.12768200

H 2.65456000 -4.00396500 -1.27536200

C 0.96793600 -5.30745800 -1.60358200

H 1.55393100 -6.06818600 -2.11072400

C -0.40779700 -5.47522000 -1.43823500

H -0.89723900 -6.36872800 -1.81430300

C -1.16594300 -4.49215800 -0.79581900

H -2.23779100 -4.61370000 -0.67246600

C -0.54427900 -3.33545300 -0.32670800

C -0.86104200 2.91749900 0.29150300

C -0.73235800 4.29151500 0.52917200

H 0.24834500 4.75368900 0.47288900

C -1.84871200 5.06123200 0.85330000

H -1.73777300 6.12322000 1.05120900

C -3.10915400 4.46133700 0.92599200

H -3.98243900 5.05526400 1.17899900

C -3.25270300 3.09786100 0.66877900

H -4.22981700 2.62714400 0.71447700

C -2.13090300 2.32876100 0.35235400

H -0.39597100 -0.08487300 1.75641600

H	-2.70890300	0.77899700	-0.82924300
H	-0.56723200	-0.49764400	-1.61529200
C	-6.36476000	1.08982100	1.37950200
C	-6.09537900	0.29785100	0.26446000
C	-5.20420100	-0.77908400	0.37271500
C	-4.59004200	-1.05617800	1.60411900
C	-4.85104600	-0.25226900	2.71140400
C	-5.73778900	0.82203400	2.59944900
H	-7.05722900	1.92132000	1.29486200
H	-6.56909500	0.51071200	-0.68639100
H	-3.91258400	-1.89830000	1.68368700
H	-4.36497100	-0.46378800	3.65832400
H	-5.94178000	1.44801200	3.46268600
C	-4.85838600	-1.63147300	-0.79431700
O	-4.04991300	-2.55151200	-0.75672600
O	-5.51670700	-1.29128900	-1.90763200
C	-5.19779400	-2.05495500	-3.08940800
H	-5.82019800	-1.63894100	-3.88089000
H	-4.13997000	-1.94691100	-3.33926900
H	-5.43070000	-3.11105700	-2.93651200
C	2.91411400	-1.16833200	-1.00289500
C	4.24683300	-1.30982500	-0.59259700
C	2.64624300	-0.62935200	-2.27006800
C	5.28916300	-0.90886100	-1.43205700
H	4.47502700	-1.71170200	0.38802200
C	3.68591800	-0.23096700	-3.10811800
H	1.61372300	-0.49510500	-2.56969900
C	5.01221800	-0.36530800	-2.68790300
H	6.31794400	-1.01487100	-1.09948200
H	3.45890400	0.20308300	-4.07753600
H	5.82441800	-0.04303100	-3.33318600
C	2.30488000	-1.93532100	1.68459700

C	2.84165400	-3.20105400	1.96163600
C	2.42348900	-0.92303800	2.64766500
C	3.49023200	-3.44612100	3.17417700
H	2.75499400	-4.00026000	1.23324100
C	3.08365800	-1.16374100	3.85247000
H	1.98951400	0.04916400	2.45188700
C	3.61750100	-2.42658600	4.12005700
H	3.89607200	-4.43313300	3.37812500
H	3.17372600	-0.36471800	4.58269400
H	4.12497900	-2.61725700	5.06151000
C	1.10441100	2.51911900	-1.72888000
C	1.98221900	3.61223200	-1.78328200
C	0.59705400	2.00226800	-2.92791300
C	2.35947100	4.15905900	-3.01076100
H	2.38675600	4.03177300	-0.86867000
C	0.96883900	2.55130900	-4.15583200
H	-0.07311900	1.14811800	-2.88387200
C	1.85655800	3.62838000	-4.20077700
H	3.04860500	4.99859300	-3.03678100
H	0.57175200	2.13235300	-5.07637800
H	2.15543700	4.05134600	-5.15564100
C	1.89710300	2.21984700	1.00936100
C	1.64615400	2.67408200	2.31093500
C	3.21473700	1.90153300	0.64248500
C	2.69098000	2.80965500	3.22695100
H	0.63076600	2.90696900	2.61473600
C	4.25611200	2.02956100	1.56110400
H	3.42772100	1.53952100	-0.35733600
C	3.99789000	2.48343900	2.85655000
H	2.48173000	3.16379600	4.23241700
H	5.26698100	1.76523800	1.26445100
H	4.80797500	2.57953500	3.57355100

Potential reactive conformer **i** (**3H**-MB)

Ru	-0.36567500	-0.24852200	0.05932700
P	1.47038000	-1.52463300	0.08542500
P	0.52348700	1.77487600	-0.13000600
N	-1.30830700	-2.27432500	0.30468200
H	-2.27621100	-2.31838500	-0.02199000
H	-1.33248100	-2.42487400	1.31250800
N	-2.26325600	0.90279700	0.07882400
H	-2.90041100	0.49786300	0.76243600
C	0.83962900	-3.15621600	-0.49048100
C	1.58856500	-4.14973600	-1.12768200
H	2.65456000	-4.00396500	-1.27536200
C	0.96793600	-5.30745800	-1.60358200
H	1.55393100	-6.06818600	-2.11072400
C	-0.40779700	-5.47522000	-1.43823500
H	-0.89723900	-6.36872800	-1.81430300
C	-1.16594300	-4.49215800	-0.79581900
H	-2.23779100	-4.61370000	-0.67246600
C	-0.54427900	-3.33545300	-0.32670800
C	-0.86104200	2.91749900	0.29150300
C	-0.73235800	4.29151500	0.52917200
H	0.24834500	4.75368900	0.47288900
C	-1.84871200	5.06123200	0.85330000
H	-1.73777300	6.12322000	1.05120900
C	-3.10915400	4.46133700	0.92599200
H	-3.98243900	5.05526400	1.17899900
C	-3.25270300	3.09786100	0.66877900
H	-4.22981700	2.62714400	0.71447700
C	-2.13090300	2.32876100	0.35235400
H	-0.39597100	-0.08487300	1.75641600
H	-2.70890300	0.77899700	-0.82924300
H	-0.56723200	-0.49764400	-1.61529200
C	-6.36476000	1.08982100	1.37950200
C	-6.09537900	0.29785100	0.26446000
C	-5.20420100	-0.77908400	0.37271500
C	-4.59004200	-1.05617800	1.60411900
C	-4.85104600	-0.25226900	2.71140400
C	-5.73778900	0.82203400	2.59944900
H	-7.05722900	1.92132000	1.29486200
H	-6.56909500	0.51071200	-0.68639100
H	-3.91258400	-1.89830000	1.68368700
H	-4.36497100	-0.46378800	3.65832400
H	-5.94178000	1.44801200	3.46268600
C	-4.85838600	-1.63147300	-0.79431700
O	-4.04991300	-2.55151200	-0.75672600
O	-5.51670700	-1.29128900	-1.90763200
C	-5.19779400	-2.05495500	-3.08940800
H	-5.82019800	-1.63894100	-3.88089000
H	-4.13997000	-1.94691100	-3.33926900
H	-5.43070000	-3.11105700	-2.93651200
C	2.91411400	-1.16833200	-1.00289500
C	4.24683300	-1.30982500	-0.59259700
C	2.64624300	-0.62935200	-2.27006800
C	5.28916300	-0.90886100	-1.43205700
H	4.47502700	-1.71170200	0.38802200
C	3.68591800	-0.23096700	-3.10811800
H	1.61372300	-0.49510500	-2.56969900
C	5.01221800	-0.36530800	-2.68790300
H	6.31794400	-1.01487100	-1.09948200
H	3.45890400	0.20308300	-4.07753600
H	5.82441800	-0.04303100	-3.33318600
C	2.30488000	-1.93532100	1.68459700
C	2.84165400	-3.20105400	1.96163600
C	2.42348900	-0.92303800	2.64766500

C	3.49023200	-3.44612100	3.17417700
H	2.75499400	-4.00026000	1.23324100
C	3.08365800	-1.16374100	3.85247000
H	1.98951400	0.04916400	2.45188700
C	3.61750100	-2.42658600	4.12005700
H	3.89607200	-4.43313300	3.37812500
H	3.17372600	-0.36471800	4.58269400
H	4.12497900	-2.61725700	5.06151000
C	1.10441100	2.51911900	-1.72888000
C	1.98221900	3.61223200	-1.78328200
C	0.59705400	2.00226800	-2.92791300
C	2.35947100	4.15905900	-3.01076100
H	2.38675600	4.03177300	-0.86867000
C	0.96883900	2.55130900	-4.15583200
H	-0.07311900	1.14811800	-2.88387200
C	1.85655800	3.62838000	-4.20077700
H	3.04860500	4.99859300	-3.03678100
H	0.57175200	2.13235300	-5.07637800
H	2.15543700	4.05134600	-5.15564100
C	1.89710300	2.21984700	1.00936100
C	1.64615400	2.67408200	2.31093500
C	3.21473700	1.90153300	0.64248500
C	2.69098000	2.80965500	3.22695100
H	0.63076600	2.90696900	2.61473600
C	4.25611200	2.02956100	1.56110400
H	3.42772100	1.53952100	-0.35733600
C	3.99789000	2.48343900	2.85655000
H	2.48173000	3.16379600	4.23241700
H	5.26698100	1.76523800	1.26445100
H	4.80797500	2.57953500	3.57355100

Potential reactive conformer **ii** (**3H**-MB)

Ru	0.18549700	-0.41946500	-1.01270600
P	0.48622200	1.58482500	-0.03776200
P	-1.90728700	-0.78638300	-0.27334000
N	0.30233100	-2.48014500	-1.78664200
C	3.05371200	1.90981900	-1.21991600
C	4.37889100	2.36139900	-1.24689700
C	4.93343800	3.05310000	-0.17101200
C	4.15088800	3.30295400	0.95488500
C	2.83010400	2.85659300	0.99424100
C	2.26160000	2.15082800	-0.07638700
C	-0.47781000	-3.50800200	-1.05610300
C	-1.95155200	-3.41053000	-1.37060300
C	-2.57658900	-4.52023700	-1.95290100
C	-3.93506400	-4.50883800	-2.26713200
C	-4.68797500	-3.36765200	-1.99841900
C	-4.07677100	-2.25401600	-1.42170700
C	-2.71133600	-2.24992100	-1.10179900
C	2.53113700	1.16435300	-2.42282400
N	2.10066800	-0.20482600	-2.05427100
H	-1.98379700	-5.40737700	-2.16124900
H	-4.39691900	-5.38188800	-2.71870700
H	-5.74817800	-3.33968400	-2.23335500
H	-4.67669400	-1.37496900	-1.21842900
H	2.23655500	3.05998500	1.87733700
H	4.56235300	3.84375100	1.80226600
H	5.96468900	3.39099800	-0.21319200
H	4.98248000	2.16222600	-2.12853000
H	1.66441900	1.66762400	-2.86067400
H	3.32077000	1.12598900	-3.18164500
H	-0.12872600	-4.51344400	-1.31831700
H	-0.28420700	-3.34973300	0.00842700
H	1.28840800	-2.72337200	-1.70392500

H	0.06900300	-2.53516100	-2.77701900
H	0.99544400	-1.03023100	0.32742300
H	-0.51368800	0.09163300	-2.49953800
H	2.00718600	-0.74291600	-2.91329600
H	2.85050000	-0.63893700	-1.52082800
C	4.15517400	-3.36966000	-1.88137000
C	3.64757800	-3.24161800	-0.58859600
C	4.01058000	-2.13884100	0.19813900
C	4.88989500	-1.17545600	-0.31408000
C	5.40324600	-1.31296000	-1.60338900
C	5.03432600	-2.40767700	-2.38962600
H	3.87201400	-4.22313400	-2.49000800
H	2.96882400	-3.98564600	-0.18831400
H	5.14682100	-0.32062400	0.30053000
H	6.08210400	-0.56267400	-1.99598500
H	5.43075200	-2.51257200	-3.39508200
C	3.47731900	-1.93219700	1.57289500
O	3.72875500	-0.95522100	2.25821900
O	2.71167400	-2.95287900	1.99309600
C	2.13156600	-2.79715000	3.30132100
H	1.56885700	-1.86537300	3.36120600
H	1.47113700	-3.65340500	3.43310500
H	2.91222700	-2.79959900	4.06694200
C	-0.30284800	3.14877000	-0.65897800
C	-0.15374200	4.38261000	-0.00352400
C	-1.02468200	3.10270300	-1.85481200
C	-0.73265100	5.53689900	-0.52715400
H	0.41124800	4.44326800	0.92107900
C	-1.60326700	4.25978400	-2.38329900
H	-1.12807500	2.14667700	-2.35728100
C	-1.46150600	5.47782200	-1.71984800
H	-0.61415900	6.48370000	-0.00744200
H	-2.16942100	4.20406400	-3.30868400
H	-1.91299900	6.37837100	-2.12671700
C	0.11480500	1.65766200	1.77186300
C	0.94310700	0.96162400	2.66793600
C	-1.02944400	2.29062000	2.27004400
C	0.64047300	0.91818600	4.02678300
H	1.82802200	0.45105800	2.30499300
C	-1.35056100	2.21924100	3.62893800
H	-1.67707100	2.84085800	1.59906100
C	-0.51548200	1.53715700	4.51249100
H	1.30305100	0.38858100	4.70582700
H	-2.25122200	2.70568200	3.99321400
H	-0.75996700	1.48848100	5.56970700
C	-3.13418700	0.55695000	-0.60629400
C	-3.64385800	1.37310100	0.41036000
C	-3.47623600	0.85019200	-1.93660500
C	-4.46362500	2.46399600	0.10671500
H	-3.40005100	1.16386200	1.44425300
C	-4.31602200	1.91934800	-2.23818300
H	-3.07262100	0.24029500	-2.73795100
C	-4.80476300	2.73923200	-1.21650600
H	-4.83719400	3.09326800	0.90971700
H	-4.57551900	2.12452100	-3.27299600
H	-5.44458800	3.58445400	-1.45284500
C	-2.30459000	-1.25830400	1.48070300
C	-3.62330000	-1.44031400	1.93039900
C	-1.25038000	-1.51515600	2.36150000
C	-3.87630800	-1.84622900	3.23913000
H	-4.45626400	-1.26172400	1.25833600
C	-1.50194100	-1.92589600	3.67313600
H	-0.23627200	-1.38475700	2.00029500
C	-2.81385600	-2.08781100	4.11642300

H	-4.90118000	-1.97716800	3.57531900
H	-0.67133800	-2.11150800	4.34770400
H	-3.01201100	-2.40290000	5.13710300

Potential reactive conformer **iii** (**3H**-MB)

Ru	0.16456200	-0.31657100	-1.11117600
P	0.48298900	1.69259500	-0.15978100
P	-1.84447700	-0.76756600	-0.20996400
N	0.20906900	-2.33763400	-2.01008700
C	3.19823100	1.71292500	-0.89861500
C	4.56839600	1.88666700	-0.68208000
C	5.06133300	2.27498500	0.56507100
C	4.17052100	2.49442200	1.61413000
C	2.79841800	2.33261000	1.40820000
C	2.29443200	1.94354300	0.16137800
C	-0.78776100	-3.35210600	-1.58346500
C	-2.20049500	-2.95423400	-1.94427600
C	-2.90920100	-3.74628400	-2.85281300
C	-4.22211700	-3.43818200	-3.21510100
C	-4.84103200	-2.32151100	-2.65823800
C	-4.14513400	-1.52054500	-1.74935300
C	-2.82713400	-1.81659600	-1.38425900
C	2.72520500	1.25406700	-2.25669600
N	2.08648000	-0.08535200	-2.18193800
H	-2.42307900	-4.62013700	-3.27969700
H	-4.75282900	-4.06698700	-3.92389600
H	-5.86403400	-2.07050600	-2.92415200
H	-4.63931200	-0.65611700	-1.32086500
H	2.11509100	2.51564600	2.22936400
H	4.53653100	2.79845700	2.59075800
H	6.12972100	2.40165700	0.71300700
H	5.25706400	1.70882500	-1.50406000
H	1.98046700	1.93978700	-2.67103300
H	3.58049700	1.23212700	-2.94171700
H	-0.56529300	-4.32121700	-2.04449600
H	-0.67662000	-3.46565000	-0.50170100
H	1.13373000	-2.70951100	-1.79807700
H	0.15603200	-2.24287900	-3.02284200
H	1.03417100	-0.95063100	0.17167400
H	-0.61135100	0.22598000	-2.55879700
H	1.91573000	-0.39740300	-3.13586200
H	2.76184500	-0.73242300	-1.77840600
C	5.02141300	-1.76841300	-1.34117800
C	4.46394500	-1.63036500	-0.06922800
C	3.53852900	-2.57490200	0.39151100
C	3.17580300	-3.65398400	-0.42595200
C	3.72387400	-3.78090800	-1.70103900
C	4.65072700	-2.83892700	-2.15988700
H	5.73980800	-1.03553300	-1.69477100
H	4.73279700	-0.78949100	0.55721300
H	2.46140100	-4.37811900	-0.04997100
H	3.43819300	-4.61683800	-2.33243400
H	5.08451500	-2.94102500	-3.15018100
C	2.91437400	-2.47817200	1.73983500
O	2.20174600	-3.34194900	2.22352600
O	3.23847600	-1.34222300	2.37960700
C	2.65420200	-1.16310800	3.68072200
H	3.10171500	-1.85707900	4.39812400
H	2.87525600	-0.13441800	3.96303200
H	1.57616200	-1.32756700	3.64797400
C	0.14729300	3.29406000	-1.04534700
C	0.67834500	4.50972600	-0.58332200
C	-0.62711900	3.29686400	-2.21010400
C	0.41759100	5.70146500	-1.25862100

H	1.29625500	4.52496700	0.30911400
C	-0.89006400	4.48953500	-2.88869200
H	-0.99897800	2.34966100	-2.58666100
C	-0.37251900	5.69455300	-2.41214500
H	0.83218800	6.63453700	-0.88732500
H	-1.49381600	4.47422600	-3.79204800
H	-0.57402600	6.62284300	-2.93932700
C	-0.25364800	2.00922600	1.50390400
C	-0.08756500	1.00553800	2.47149800
C	-0.99004400	3.15062700	1.83871300
C	-0.62999800	1.14449400	3.74659200
H	0.46558100	0.11398600	2.20586000
C	-1.54031200	3.28815200	3.11585000
H	-1.15774700	3.92847100	1.10392300
C	-1.36001000	2.29098500	4.07405500
H	-0.49139600	0.35497800	4.47996200
H	-2.12110300	4.17445700	3.35461000
H	-1.79189500	2.39994400	5.06478800
C	-2.99212500	0.62870400	0.17052800
C	-3.52507100	0.86958500	1.44236200
C	-3.26471100	1.54534800	-0.85780600
C	-4.31885900	1.99410500	1.67812500
H	-3.30543100	0.19751600	2.26214100
C	-4.05862800	2.66625400	-0.62394800
H	-2.84302000	1.37562200	-1.84158000
C	-4.59038800	2.89424800	0.64837000
H	-4.71163300	2.17058800	2.67513800
H	-4.25364200	3.36512000	-1.43242100
H	-5.20297000	3.77154400	0.83560700
C	-2.03606000	-1.85495400	1.28912200
C	-3.29939500	-2.30249100	1.71072800
C	-0.90165700	-2.30945100	1.96819200
C	-3.42071700	-3.15324000	2.80872300
H	-4.19186300	-1.98559100	1.17994600
C	-1.01840900	-3.16864300	3.06354400
H	0.07411900	-2.00190500	1.61037300
C	-2.27900900	-3.58523200	3.49185300
H	-4.40487000	-3.48523600	3.12766100
H	-0.11834100	-3.51607300	3.56071700
H	-2.37533100	-4.25325600	4.34327500

Potential reactive conformer **iv** (**3H**-MB)

Ru	0.04642100	-0.28997100	-1.15452900
P	0.95770200	1.39288400	0.01388300
P	-2.03999800	-0.20313100	-0.30240200
N	-0.49433200	-2.17861600	-2.18569300
C	3.47704800	1.12729100	-1.29233500
C	4.86567000	1.29481600	-1.37892500
C	5.61434000	1.75567100	-0.29745500
C	4.96701900	2.06207600	0.89809900
C	3.58418200	1.91609300	0.99216100
C	2.81864600	1.44558600	-0.08527700
C	-1.75786900	-2.87838800	-1.84554300
C	-2.97172000	-2.05616900	-2.20408500
C	-3.84753100	-2.52729300	-3.18592500
C	-4.98861100	-1.80547100	-3.54367200
C	-5.26036000	-0.59493500	-2.91016000
C	-4.39224800	-0.11274200	-1.92707100
C	-3.24493400	-0.82643800	-1.56425300
C	2.75056600	0.63228000	-2.52025600
N	1.94238100	-0.57836500	-2.24046900
H	-3.63126200	-3.47452900	-3.67394200
H	-5.65701700	-2.18875800	-4.30899800
H	-6.14716700	-0.02486400	-3.17228800

H	-4.61736700	0.82626900	-1.43406800
H	3.09329500	2.17993800	1.92122400
H	5.53119800	2.42360500	1.75289700
H	6.69026900	1.87084600	-0.38914600
H	5.36375700	1.05363300	-2.31426000
H	2.05989500	1.38954200	-2.90137500
H	3.49116200	0.42621800	-3.30139200
H	-1.81340000	-3.84183700	-2.36523100
H	-1.72266600	-3.08076500	-0.77163200
H	0.27147900	-2.80514600	-1.94419800
H	-0.46488700	-2.03806400	-3.19433100
H	0.60289200	-1.27104800	0.06813000
H	-0.49000500	0.59088000	-2.56035300
H	1.67082200	-0.97730100	-3.13726400
H	2.53686400	-1.27227200	-1.78793800
C	0.79046000	-4.71840600	-0.03018100
C	1.98452800	-4.10180500	-0.39927500
C	2.68352200	-3.32207800	0.53331600
C	2.19464100	-3.19021500	1.83846400
C	0.99974700	-3.80303700	2.20070000
C	0.28885900	-4.55636700	1.26387700
H	0.24904500	-5.32347300	-0.75104600
H	2.37776200	-4.22390300	-1.40201400
H	2.75509600	-2.59507000	2.55023400
H	0.61411800	-3.68453000	3.20709600
H	-0.65186300	-5.02017000	1.54428700
C	3.94450300	-2.61943200	0.18748900
O	4.67676800	-2.06775200	0.98724600
O	4.20124500	-2.65536400	-1.14434500
C	5.47345500	-2.11566000	-1.55244800
H	6.27555700	-2.80248900	-1.26766500
H	5.42491000	-2.03145400	-2.63809000
H	5.64552300	-1.14267100	-1.09548500
C	0.70541000	3.19974000	-0.36887000
C	0.96141400	4.22358600	0.55509900
C	0.34179800	3.55014300	-1.67469100
C	0.81869200	5.56250300	0.18964300
H	1.26613500	3.97992800	1.56697000
C	0.21199300	4.88899700	-2.04763100
H	0.13868900	2.75751600	-2.38862500
C	0.44120300	5.90012300	-1.11265500
H	1.00629600	6.34315000	0.92172500
H	-0.07666600	5.14038100	-3.06464600
H	0.33133500	6.94313200	-1.39580000
C	0.65871600	1.31061600	1.83228300
C	1.35950400	0.36471900	2.59926700
C	-0.38223700	2.02732900	2.43553400
C	1.04021500	0.15678600	3.93939300
H	2.14619800	-0.22117000	2.13773400
C	-0.71713400	1.80251800	3.77336200
H	-0.94373600	2.75475100	1.86267100
C	-0.00617700	0.87184600	4.53056800
H	1.59753100	-0.57428100	4.51849700
H	-1.53773200	2.35771400	4.21842200
H	-0.26583000	0.70051700	5.57121200
C	-2.78523600	1.41363000	0.19764800
C	-3.49334300	1.60126200	1.39270300
C	-2.55506100	2.52134100	-0.63097700
C	-3.94056200	2.87261400	1.75877700
H	-3.67841500	0.76305400	2.05331700
C	-3.00030900	3.79109200	-0.26677000
H	-2.00293100	2.38164900	-1.55165400
C	-3.68836300	3.97193700	0.93568300
H	-4.47692100	3.00398800	2.69442200

H -2.78982000 4.63901200 -0.91170100

H -4.02402000 4.96251300 1.22918800

C -2.52851600 -1.25829900 1.14467700

C -3.81720000 -1.79900600 1.27752000

C -1.59723100 -1.47062400 2.16688300

C -4.16374300 -2.53584900 2.41129200

H -4.55513000 -1.63718100 0.49861400

C -1.95134400 -2.18400300 3.31292500

H -0.59440500 -1.08200900 2.05555000

C -3.23262200 -2.72442200 3.43613400

H -5.16269500 -2.95425600 2.49820600

H -1.22181000 -2.31976000 4.10594500

H -3.50527900 -3.28874200 4.32354100

2H^{s}-IN1$_{PhCHO}$

Ru 0.55848900 0.80934900 -0.38766000

P -1.08459700 1.95755100 0.70242800

P 2.43320800 1.00219400 0.89171900

N -1.06002600 0.36097700 -1.80045100

H -0.96253900 -0.71681400 -1.79371300

H -0.81178100 0.66005700 -2.74233300

N 1.86313900 -0.44671100 -1.63934000

H 1.95551000 -0.05049200 -2.57348600

C -2.59458700 1.62107400 -0.33925300

C -3.88709000 2.07991800 -0.04424300

H -4.05914800 2.68165500 0.84458300

C -4.96389000 1.77632500 -0.87861100

H -5.95926100 2.13798000 -0.63725800

C -4.75334200 1.00148500 -2.02412000

H -5.58593900 0.75489700 -2.67690300

C -3.47368900 0.53614900 -2.32772200

H -3.30768900 -0.07756000 -3.20945600

C -2.39189000 0.84303500 -1.49120700

C 3.63157800 -0.16185000 0.05600900

C 4.91299500 -0.47139100 0.53676400

H 5.27521700 -0.00820200 1.45110000

C 5.73655700 -1.37102500 -0.14212900

H 6.72530100 -1.60225900 0.24366600

C 5.27935100 -1.97567500 -1.31789200

H 5.90995400 -2.68304800 -1.84891500

C 4.00820600 -1.67544200 -1.80933700

H 3.64352100 -2.14934000 -2.71698500

C 3.18371300 -0.76858300 -1.12990900

H 0.96727400 2.17051400 -1.07634300

C 3.40135500 2.59468800 0.99956900

H 2.82183900 3.32115300 1.57740400

H 4.37486100 2.45076300 1.47851000

H 3.54274700 2.98957100 -0.00892600

C 2.50024000 0.44312800 2.67423000

H 3.52048300 0.43489100 3.07045700

H 1.89340400 1.12613000 3.27672700

H 2.07398000 -0.55998600 2.75160200

C -1.06667600 3.81857200 0.83796300

H -0.25930400 4.11385200 1.51513900

H -0.86267800 4.24146200 -0.14824700

H -2.01440600 4.21234100 1.21820500

C -1.66045400 1.48745600 2.41588800

H -0.87188900 1.73392700 3.13374700

H -2.57541700 2.01600000 2.70158100

H -1.84204500 0.41027500 2.44848300

C -2.74215600 -3.15215100 2.55855600

C -1.59070200 -2.66519300 1.93674500

C -1.47113200 -2.66783200 0.53863700

C -2.52600900 -3.17501400 -0.22664800

C -3.68301000 -3.66256000 0.39224900

C -3.79724000 -3.65174800 1.78475000

H -2.81839200 -3.14489100 3.64316200

H -0.77110300 -2.28203900 2.54357300

H -2.41245100 -3.18650100 -1.30606200

H -4.49504200 -4.05503500 -0.21502700

H -4.69503600 -4.03143300 2.26517700

C -0.20020600 -2.15726300 -0.14014700

O -0.22266900 -2.13193700 -1.50158500

H 1.21416500 -1.29468100 -1.72650100

H -0.02519600 -1.10734400 0.32465200

H 0.65638300 -2.73436100 0.26759200

2H^{s}-IN2$_{PhCHO}$

Ru -0.85940400 -0.60266800 -0.23851900

P -0.42225400 -2.78462000 0.27848600

P -3.03199100 -0.26906400 0.37142300

N 1.20616400 -0.61433300 -0.71811900

H 1.41003700 0.62696600 0.63846200

H 1.59935000 0.04207000 -1.39050500

N -0.97811800 1.55914700 -0.76516000

H -0.57907700 1.70130500 -1.69153300

C 1.34733000 -2.98222400 -0.21041000

C 2.08223800 -4.17187100 -0.15268300

H 1.61936800 -5.07249600 0.24632200

C 3.39786600 -4.23368700 -0.61817500

H 3.95674300 -5.16355500 -0.57563600

C 3.97473100 -3.07407200 -1.15840000

H 4.99238700 -3.10547100 -1.54087200

C 3.26444400 -1.87890100 -1.21249700

H 3.72987500 -0.98752900 -1.62869800

C 1.93487600 -1.79377100 -0.72534100

C -3.36540600 1.49835200 -0.15342600

C -4.60960800 2.14112400 -0.06085000

H -5.46302900 1.60820400 0.35032100

C -4.77710000 3.45576200 -0.49939000

H -5.74945500 3.93453200 -0.42587400

C -3.68954500 4.15102600 -1.03773600

H -3.81039300 5.17333000 -1.38426500

C -2.44273400 3.52979400 -1.12613100

H -1.59077300 4.06818000 -1.53478300

C -2.28054700 2.21111400 -0.68461000

H -1.45917400 -1.12520800 -1.58442400

C -4.46017500 -1.18765900 -0.41239200

H -4.41735700 -2.23302000 -0.09109300

H -5.43363100 -0.77275400 -0.13279000

H -4.34516600 -1.15671000 -1.49830200

C -3.61118400 -0.24137100 2.15486600

H -4.62266800 0.16545000 2.25665900

H -3.60026200 -1.26379500 2.54478800

H -2.91705300 0.36134500 2.74636600

C -1.30453500 -4.18140200 -0.58704400

H -2.35626700 -4.18747300 -0.28454300

H -1.25134900 -4.01272100 -1.66485300

H -0.85745600 -5.15116300 -0.34723800

C -0.46764600 -3.39099900 2.04780200

H -1.50348300 -3.43356300 2.39838900

H -0.01972000 -4.38605300 2.13737900

H 0.08784400 -2.69222800 2.67870300

C 5.52269500 2.30016800 -0.10088900

C 4.54173300 1.71892800 0.70926000

C 3.42214100 2.45566900 1.11955900

C 3.30789600 3.79234900 0.70743200

C 4.28407000 4.37719300 -0.10204600

C	5.39546300	3.63053600	-0.50926000
H	6.38530900	1.71570200	-0.40916800
H	4.64432200	0.68245500	1.02221700
H	2.44698300	4.37612000	1.02564800
H	4.18380100	5.41486900	-0.40906000
H	6.15825600	4.08488600	-1.13548400
C	2.33716600	1.81715100	1.96399800
O	1.19692100	1.42239700	1.19780700
H	-0.32090000	1.97365900	-0.09190100
H	2.74546300	0.95299100	2.50586200
H	1.96824300	2.53218900	2.70679000

$2H^{s}$-IN3$_{PhCHO}$

Ru	-0.98981600	-0.50810800	-0.06451100
P	-0.63602500	-2.75825600	0.20770200
P	-3.25064300	-0.18926400	0.12977100
N	1.13033300	-0.53930500	-0.51390200
H	1.55848800	0.82100100	0.57879200
H	1.44423900	0.04189500	-1.28934700
N	-1.01240300	1.67282600	-0.54095600
H	-0.67668700	1.72152100	-1.50177300
C	1.16803200	-2.94056800	-0.11399200
C	1.87261300	-4.15215900	-0.05800400
H	1.36596900	-5.05542600	0.27726000
C	3.20799600	-4.22905400	-0.45136600
H	3.74480000	-5.17189400	-0.40841300
C	3.83704900	-3.06541500	-0.92656100
H	4.87227100	-3.10931100	-1.25737900
C	3.15978300	-1.85343100	-0.97443900
H	3.66832800	-0.95900900	-1.32786600
C	1.80914300	-1.74629300	-0.54395800
C	-3.45052500	1.65043500	-0.15926500
C	-4.67201300	2.33853400	-0.08863400
H	-5.58757800	1.79288500	0.12398800
C	-4.73368200	3.71955600	-0.28073100
H	-5.68755900	4.23560300	-0.21896500
C	-3.56230200	4.43540100	-0.54880500
H	-3.59932800	5.51107100	-0.69479900
C	-2.34121000	3.76473800	-0.63031400
H	-1.42707000	4.31406400	-0.84193700
C	-2.28457300	2.37829400	-0.44071000
H	-1.26375600	-0.81866200	-1.60833700
C	-4.44168200	-0.92186900	-1.10777100
H	-4.48945900	-2.00355400	-0.94926700
H	-5.44835100	-0.50289600	-1.01198700
H	-4.05864400	-0.73891600	-2.11398800
C	-4.22600100	-0.43780100	1.70827200
H	-5.26017100	-0.08846200	1.62498400
H	-4.23400800	-1.50625800	1.94560300
H	-3.72962300	0.09325200	2.52469400
C	-1.44194000	-3.99143000	-0.93593400
H	-2.51818300	-4.02327400	-0.73818900
H	-1.28308800	-3.66508300	-1.96585600
H	-1.02324500	-4.99363100	-0.80154200
C	-0.91109700	-3.59623900	1.85526600
H	-1.98289400	-3.63383600	2.07383600
H	-0.51376300	-4.61643200	1.85746900
H	-0.41251100	-3.01890800	2.63806000
C	6.05243700	1.59249700	0.03357600
C	4.93261900	1.34129900	0.83408300
C	3.89111200	2.27389300	0.92332000
C	3.99357900	3.46880100	0.19373200
C	5.10749500	3.72373400	-0.60794400
C	6.14230700	2.78423700	-0.68960200

H	6.85153400	0.85834700	-0.02348300
H	4.86542100	0.40958000	1.39077400
H	3.19215100	4.20125800	0.25689300
H	5.17389000	4.65527200	-1.16380400
H	7.01171400	2.98250100	-1.31033900
C	2.66816200	1.99915000	1.77739800
O	1.49705000	1.72110600	1.01403700
H	-0.29204200	2.11261100	0.04237500
H	2.88108300	1.17047500	2.46770600
H	2.43094100	2.88073200	2.38367100
H	-0.30758500	0.12893900	1.64188400
H	-0.87909500	-0.37723700	1.84167400

2H^{s}-IN4$_{PhCHO}$

Ru	0.97681100	0.57371000	-0.32140000
P	0.59564900	2.68319400	0.36277700
P	3.05668700	0.16153600	0.43291600
N	-1.08625100	0.77869500	-1.19068500
H	-1.62282100	-0.03038600	-0.86243800
H	-0.97436700	0.69300300	-2.19896300
N	1.15893200	-1.51352800	-1.13754400
H	1.11033100	-1.41065600	-2.14922100
C	-1.15870300	3.03127300	-0.18859300
C	-1.85881500	4.21935000	0.07226600
H	-1.37281800	5.02306900	0.61906100
C	-3.17703100	4.38851400	-0.35475500
H	-3.70388400	5.31432300	-0.14114400
C	-3.81620600	3.36052900	-1.05547000
H	-4.84298700	3.48092000	-1.38911700
C	-3.13089900	2.17537200	-1.32796300
H	-3.62107800	1.37195000	-1.87272800
C	-1.80758600	2.01216300	-0.89926700
C	3.38180200	-1.61111100	-0.07348900
C	4.54765600	-2.32976100	0.23306600
H	5.34822400	-1.84774300	0.78793200
C	4.69849700	-3.66130800	-0.15785800
H	5.60694900	-4.20235000	0.09169800
C	3.67423100	-4.29574600	-0.86808800
H	3.77979900	-5.33296300	-1.17309700
C	2.51196400	-3.59170000	-1.18700000
H	1.71252600	-4.07751600	-1.74171400
C	2.36742900	-2.25505300	-0.79512500
H	1.59591300	1.13781200	-1.78784400
C	4.56440400	1.04376400	-0.23433500
H	4.53917000	2.08353000	0.10712800
H	5.50170000	0.58489500	0.09743100
H	4.51038300	1.04006200	-1.32504800
C	3.47759400	0.12479300	2.25479700
H	4.47711500	-0.27765400	2.44895800
H	3.42859100	1.14662800	2.64415200
H	2.72542700	-0.47607300	2.77032800
C	1.52665700	4.14673100	-0.33567900
H	2.56094000	4.10256000	0.02020300
H	1.53560500	4.06017700	-1.42423500
H	1.08968700	5.10573700	-0.03817800
C	0.53626900	3.16456900	2.16900700
H	1.55035900	3.10894300	2.57689800
H	0.15099600	4.17787400	2.32235600
H	-0.08785000	2.44241700	2.69947700
C	-5.81619300	-1.71296400	0.44593300
C	-4.51532500	-1.50844500	0.91070700
C	-3.58236000	-2.55679000	0.91789100
C	-3.98377300	-3.81597500	0.45339200
C	-5.28523600	-4.02662500	-0.01372600

C -6.20481300 -2.97452000 -0.01898800
H -6.52905500 -0.89265000 0.45320800
H -4.21832700 -0.52773400 1.27519300
H -3.27111900 -4.63754900 0.45879800
H -5.58052400 -5.01021600 -0.36902300
H -7.21788300 -3.13609600 -0.37689400
C -2.16751800 -2.32029500 1.39512400
O -1.39348700 -1.71240400 0.35944600
H 0.32944400 -2.03077400 -0.83288100
H -2.17414800 -1.66689700 2.27846000
H -1.71272900 -3.27661500 1.69113300
H -0.71312100 -1.07640800 0.75918500
H 0.27168500 -0.07532900 1.15810300

2H^{s}-TS1$_{PhCHO}$

Ru -0.55718300 -0.84753900 -0.35357800
P 1.09275400 -1.92482000 0.79762000
P -2.43060600 -0.95347300 0.94183900
N 1.04744800 -0.46894400 -1.77947400
H 0.93129000 0.68341900 -1.78039900
H 0.80292000 -0.75520300 -2.72675500
N -1.86871900 0.33056200 -1.68362300
H -1.97273800 -0.15061200 -2.57569900
C 2.59031700 -1.65840500 -0.27460600
C 3.88303100 -2.10902000 0.03049900
H 4.06547000 -2.64600300 0.95814400
C 4.94586800 -1.88499900 -0.84573600
H 5.94197800 -2.24049700 -0.59853700
C 4.71671000 -1.19934000 -2.04410400
H 5.53714000 -1.01694300 -2.73273900
C 3.43788600 -0.73803700 -2.35501300
H 3.26216100 -0.18904600 -3.27677800
C 2.36619700 -0.96032800 -1.47562000
C -3.62747600 0.16553200 0.03752800
C -4.90424000 0.51439400 0.50415000
H -5.26644700 0.10861900 1.44526800
C -5.72255200 1.38233300 -0.22085200
H -6.70693200 1.64444400 0.15629600
C -5.26684500 1.91593700 -1.43084900
H -5.89365900 2.59804500 -1.99799400
C -4.00162500 1.57450700 -1.91078800
H -3.63874100 1.99004200 -2.84742000
C -3.18407600 0.70018300 -1.18349600
H -0.97194900 -2.24693500 -0.94613500
C -3.41094200 -2.53007100 1.14231500
H -2.83965200 -3.22192300 1.76891800
H -4.38730100 -2.35225400 1.60365300
H -3.54637100 -2.98680300 0.15942600
C -2.50710200 -0.28908000 2.68855900
H -3.52760800 -0.26622000 3.08385100
H -1.89585800 -0.93104700 3.33030000
H -2.08904200 0.72018100 2.70917900
C 1.06803700 -3.77194800 1.05730400
H 0.26502000 -4.01994500 1.75815900
H 0.85736700 -4.25794900 0.10207600
H 2.01787600 -4.14182400 1.45581600
C 1.67358100 -1.34016400 2.47366100
H 0.89506100 -1.54717200 3.21449500
H 2.59691600 -1.84078600 2.78204500
H 1.84613300 -0.26154500 2.43624600
C 2.62117200 3.42416500 2.47167000
C 1.52061600 2.85697200 1.82595300
C 1.49170300 2.73144700 0.42867300
C 2.58603400 3.18882100 -0.31211900

C	3.69184100	3.75648200	0.33123300
C	3.71522400	3.87563300	1.72325000
H	2.62736000	3.51561600	3.55505600
H	0.67105900	2.51043000	2.41324200
H	2.54653900	3.09557400	-1.39268000
H	4.53579000	4.10813500	-0.25709000
H	4.57374800	4.31698700	2.22232900
C	0.27421500	2.13357400	-0.27506400
O	0.39090900	1.96778200	-1.63088200
H	-1.24181000	1.15576200	-1.85364300
H	0.07348100	1.14123800	0.27083000
H	-0.60921500	2.74522500	-0.00444100

2H^{s}-TS2$_{PhCHO}$

Ru	-0.87746400	-0.61286100	-0.31718800
P	-0.03213300	-2.61614900	0.33181400
P	-3.00267600	-0.61691900	0.47108100
N	1.14918600	-0.36260600	-1.20588400
H	1.44200500	0.53396600	-0.76026700
H	1.06791000	-0.19143200	-2.20634300
N	-1.46733100	1.39401800	-1.08966700
H	-1.34498900	1.42375300	-2.10012000
C	1.73128800	-2.57187500	-0.27595200
C	2.67253900	-3.59202400	-0.06908900
H	2.38834900	-4.48802400	0.47656600
C	3.97520600	-3.47600700	-0.55601000
H	4.69259400	-4.27427500	-0.38796500
C	4.35185300	-2.32633100	-1.25854100
H	5.36531900	-2.22339400	-1.63563600
C	3.42669000	-1.30353700	-1.47015300
H	3.71836500	-0.40197800	-2.00247100
C	2.11814100	-1.42415500	-0.98452300
C	-3.66397000	1.05850900	-0.02083300
C	-4.94351200	1.53688000	0.30012700
H	-5.62741100	0.91243200	0.86935200
C	-5.35637200	2.80809200	-0.10146700
H	-6.34985800	3.16471600	0.15511200
C	-4.48405500	3.62003600	-0.83425400
H	-4.79507100	4.61228700	-1.14842600
C	-3.20838500	3.15714200	-1.15929600
H	-2.52320800	3.78689400	-1.72142300
C	-2.79603800	1.88014300	-0.75601500
H	-1.42849200	-1.33631300	-1.66144700
C	-4.30536900	-1.78227000	-0.18403100
H	-4.06695800	-2.79818800	0.14629600
H	-5.30904500	-1.52070700	0.16560800
H	-4.27587400	-1.75902600	-1.27542700
C	-3.37670900	-0.67601100	2.30016800
H	-4.43336200	-0.49228800	2.51898800
H	-3.10457100	-1.66654900	2.67723600
H	-2.76014700	0.06849800	2.80924400
C	-0.66676800	-4.23132000	-0.35488900
H	-1.67947300	-4.40094500	0.02375300
H	-0.71466300	-4.15218800	-1.44304700
H	-0.03432000	-5.07865400	-0.07153000
C	0.16781000	-3.06417800	2.13491300
H	-0.82578800	-3.20162200	2.57351500
H	0.74688000	-3.98226800	2.27733400
H	0.65975500	-2.23564500	2.64976900
C	5.24802700	1.90116200	1.16524600
C	3.87402600	1.89793600	1.42163100
C	3.03077300	2.86801800	0.85925800
C	3.60261500	3.84685200	0.03376400
C	4.97633600	3.85707800	-0.22799400

C	5.80452100	2.88230700	0.33753500	O	0.87484400	1.98543000	0.19154500
H	5.88494300	1.14361500	1.61511800	H	-0.71710800	1.98190200	-0.66473400
H	3.44655200	1.13454500	2.06832500	H	1.35167200	2.49253000	2.14152000
H	2.96246800	4.60883900	-0.40576100	H	1.13719600	3.85846400	1.03348400
H	5.40091500	4.62745900	-0.86694400	H	0.19670900	0.91801900	0.81918400
H	6.87334600	2.89080800	0.14093700	H	-0.23575400	0.23691900	1.23856700
C	1.52967700	2.82796200	1.10056900				

VII. References

1 Sheldrick, G. M. SHELXS-90, Program for structure solution. *Acta Crystallogr. Sect. A* **46**, 467-473 (1990).

2 Sheldrick, G.. M. SHELXL-97, Program for crystal structure refinement. University of Göttingen: Göttingen, Germany, 1997.
